# Sex-Related Differences in Gene Expression by Porcine Aortic Valvular Interstitial Cells

**DOI:** 10.1371/journal.pone.0039980

**Published:** 2012-07-10

**Authors:** Chloe M. McCoy, Dylan Q. Nicholas, Kristyn S. Masters

**Affiliations:** 1 Department of Biomedical Engineering, University of Wisconsin, Madison, Wisconsin, United States of America; 2 Department of Mechanical Engineering, University of Wisconsin, Madison, Wisconsin, United States of America; Brigham and Women’s Hospital, Harvard Medical School, United States of America

## Abstract

While many large-scale risk factors for calcific aortic valve disease (CAVD) have been identified, the molecular etiology and subsequent pathogenesis of CAVD have yet to be fully understood. Specifically, it is unclear what biological phenomena underlie the significantly higher occurrence of CAVD in the male population. We hypothesized the existence of intrinsic, cellular-scale differences between male and female valvular interstitial cells (VICs) that contribute to male sex being a risk factor for CAVD. Differences in gene expression profiles between healthy male and female porcine VICs were investigated via microarray analysis. Mean expression values of each probe set in the male samples were compared to the female samples, and biological processes were analyzed for overrepresentation using Gene Ontology term enrichment analysis. There were 183 genes identified as significantly (fold change>2; P<0.05) different in male versus female aortic valve leaflets. Within this significant gene list there were 298 overrepresented biological processes, several of which are relevant to pathways identified in CAVD pathogenesis. In particular, pathway analysis indicated that cellular proliferation, apoptosis, migration, ossification, angiogenesis, inflammation, and extracellular matrix reorganization were all significantly represented in the data set. These gene expression findings also translated into functional differences in VIC behavior in the *in vitro* environment, as sex-related differences in proliferation and apoptosis were confirmed in VIC populations cultured *in vitro*. These data suggest that a sex-related propensity for CAVD exists on the cellular level in healthy subjects, a phenomenon that could have significant clinical implications. These findings also strongly support discontinuing the use of mixed-sex VIC cultures, thereby changing the current standard in the field.

## Introduction

Calcific aortic valve disease (CAVD) is defined by non-rheumatic calcification and thickening of the aortic valve leaflets and can range from pre-clinical sclerosis to symptomatic stenosis [1], [2]. The disease affects 26% of Americans over the age of 65 and is associated with a 50% increase risk of death from cardiovascular causes [3], [4]. There are currently no treatments for halting the progression or preventing onset of the disease, and the etiology and pathogenesis of CAVD have yet to be well described. While the etiology of CAVD remains unclear, advanced age, smoking, hyperlipidemia, atherosclerosis, and male sex have all been identified as risk factors for development and progression of the disease [3], [5]–[7]. After age, sex is the most significant risk factor for CAVD. Specifically, male sex is associated with a two-fold increase in the risk of aortic valve disease [3] and a 5% cumulative incidence rate of aortic valve calcium deposition, compared to 3.3% in women [5].

As noted in two recent reports [8], [9], every cell has a sex, and analyzing how sex can affect behavior on the cellular and intracellular scale is an emerging area of research. It is well known that many cardiovascular diseases affect men differently than women, and there is evidence that the sex of research material derived from animals and humans plays a role in downstream experimental outcomes [8], [10]. Aside from these observations, there is relatively little research to explain the pathogenesis behind the sex-related differences in CAVD. CAVD affects both men and women, but the severity of the disease and the progression to advanced disease stages appear to differ between the two sexes [5], [7]; women are also more likely to have preserved myocardial function in response to aortic stenosis [11]. Differences in CAVD have also been identified in mice, with significantly thicker valve leaflets, aortic regurgitation, and vascular lipid deposition in aged male mice compared to aged females, even after controlling for estrogens [12]. Although there are likely numerous anatomic and physiologic factors that influence these sex-related CAVD differences, we propose herein that cellular-scale differences in the behavior of male and female valvular interstitial cells (VICs) also play a role in this disease process. No previous study has examined VICs following separation by sex.

The aortic valve is populated by two main cell types: valvular endothelial cells that line the blood-contacting surfaces, and VICs that comprise the bulk of the valve leaflet and are thought to be the predominant cell type involved in valve calcification [1]. In response to injury or stress, quiescent VICs temporarily become activated to a myofibroblast-like phenotype that is associated with higher levels of proliferation, apoptosis, and extracellular matrix (ECM) remodeling [2], [13]. In CAVD, it is hypothesized that VICs lose their ability to return to a quiescent state after the stressor has been resolved, and instead either remain activated or adopt an osteoblast-like pathological phenotype.

Although much remains to be learned about the etiology of CAVD, analyses of calcified human valve explants have identified several hallmark characteristics of CAVD, such as: presence of inflammatory cells/molecules, angiogenesis, lipid deposition, extracellular matrix disarray, elevated cellular migration, proliferation, and apoptosis, and increased transforming growth factor-β1 (TGF-β1) expression [1], [14]–[16]. Histopathological analyses of diseased human valves have revealed numerous inflammatory cell types present within calcified lesions, including T lymphocytes and macrophages, when compared to non-calcified controls [2], [17]. In addition, aortic valve lesions have also been found to contain higher levels of interleukins and transforming growth factor-beta1 (TGF-β1), which are expressed during inflammation and are suspected contributors to the calcific process [15], [18]–[20]. CAVD can also be associated with deposition and oxidation of low density lipoprotein (LDL) cholesterol [2], [17], [18], and elevated LDL in the blood has also been identified as a CAVD risk factor [3], [21]. Accumulation of oxidized lipids in early valve lesions can enhance inflammation, apoptosis, and MMP expression [19], with each of these cellular processes known to be upregulated in CAVD [22]. Finally, in contrast to the negligible angiogenic activity present in healthy valves, angiogenesis and the expression of vascular endothelial growth factor (VEGF) and VEGF receptor 2 (VEGFR-2, or KDR) are all significantly upregulated in human CAVD explants when compared to healthy controls [16], [22], [23].

Several intracellular signaling differences have also been noted in diseased versus healthy valves. For instance, examination of calcified human valve explants has revealed high levels of phosphorylated ERK compared to healthy valves [24], and subsequent *in vitro* VIC studies have shown the MAPK/ERK pathway to be involved in VIC calcification and osteoblastic differentiation, with inhibition of the pathway resulting in dramatic reduction of calcification *in vitro* [25]. Meanwhile, work with cardiac fibroblasts found that activation of the MAPK pathway was more pronounced and persistent in male cells compared to female cells [26], indicating a possible sex-related difference in basal expression of the pathway between males and females in other tissues.

This microarray study was designed to identify intrinsic differences between male and female VICs in the context of CAVD-related gene expression pathways as well as to probe for molecular pathways that may begin to explain aspects of sex-dependent disease disparities seen in CAVD. We also investigated whether these intrinsic sex-related differences translated to differences in cellular function following *in vitro* culture of VICs. The identification of sex-related, CAVD-relevant differences on the cellular scale has the potential to significantly alter both our pursuit of CAVD treatments and our knowledge of valve biology. This study also aims to investigate the appropriateness of the current standard practice of using mixed-sex porcine VIC cultures to study valve disease. Thus, at a minimum, the findings presented herein provide evidence to influence the manner in which *in vitro* studies of valve biology and CAVD are currently performed.

## Results

### Microarray

Gene expression values from male VICs were compared to those of female VICs obtained from healthy pigs. Out of 20,201 *Sus Scrofa* genes represented on the microarray, 183 were deemed significantly different (fold change>2; P<0.05) in male versus female VICs after preprocessing and statistical analysis (Table S2). From the significant genes list, 298 biological processes were found to be overrepresented in males using GO enrichment analysis (Table S3). Sorting of this list revealed categorical groupings of biological processes that corresponded to cellular functions and phenomena that are known to be altered in CAVD (summarized in Table 1; expanded information found in Table S4). Specifically, these categories included calcification, cell proliferation, angiogenesis, ECM remodeling, cell adhesion, cell migration, and inflammation.

**Table 1:** Significant biological processes determined by GO enrichment analysis and categorized according to disease-related pathway grouping (expanded table available in Table S4).

GO BP ID and PROCESS	P value
**CALCIFICATION**	
GO:0060349: bone morphogenesis	0.039
GO:0001649: osteoblast differentiation	0.012
GO:0045668: neg. regulation of osteoblast differentiation	0.039
GO:0001503: ossification	0.001
GO:0060348: bone development	0.042
GO:0030278: regulation of ossification	0.003
GO:0003416: endochondral bone growth	0.001
GO:0060351: cartilage development involved in endochondral bone morphogenesis	0.002
GO:0060350: endochondral bone morphogenesis	0.012
GO:0045667: regulation of osteoblast differentiation	0.023
GO:0071107: response to parathyroid hormone stimulus	0.026
**PROLIFERATION**	
GO:0042127: regulation of cell proliferation	0.004
GO:0008283: cell proliferation	0.008
GO:0008284: pos. regulation of cell proliferation	0.036
GO:0008285: neg. regulation of cell proliferation	0.012
GO:0033002: muscle cell proliferation	0
GO:0048659: smooth muscle cell proliferation	0.001
GO:0048660: regulation of smooth muscle cell proliferation	0.001
**ECM REMODELING**	
GO:0010716: neg. regulation of ECM disassembly	0.013
GO:0030198: ECM organization	0.023
GO:0022617: ECM disassembly	0.039
**ADHESION**	
GO:0016337: cell-cell adhesion	0.008
GO:0033632: regulation of cell-cell adhesion by integrin	0.013
GO:0007155: cell adhesion	0.019
GO:0022610: biological adhesion	0.019
**MIGRATION**	
GO:0030336: neg. regulation of cell migration	0
GO:0040012: regulation of locomotion	0.002
GO:0030334: regulation of cell migration	0.004
GO:0040011: locomotion	0.007
GO:0016477: cell migration	0.009
GO:0048870: cell motility	0.01
GO:0051451: myoblast migration	0.026
GO:0014812: muscle cell migration	0.039
GO:0050921: pos. regulation of chemotaxis	0.039
GO:0050920: regulation of chemotaxis	0.048
**INFLAMMATION**	
GO:0071105: response to interleukin-11	0.013
GO:0032604: GMCF production	0.026
GO:0032645: regulation of GMCF production	0.026
GO:0032672: regulation of interleukin-3 production	0.026
GO:0042223: interleukin-3 biosynthetic process	0.026
GO:0042253: GMCF biosynthetic process	0.026
GO:0045399: regulation of interleukin-3 biosynthetic process	0.026
GO:0045401: pos. regulation of interleukin-3 biosynthetic process	0.026
GO:0045423: regulation of GMCF biosynthetic process	0.026
GO:0045425: pos. regulation of GMCF biosynthetic process	0.026
**ANGIOGENESIS**	
GO:0001944: vasculature development	0.003
GO:0001568: blood vessel development	0.01
GO:0048514: blood vessel morphogenesis	0.004
GO:0001525: angiogenesis	0.021
GO:0048844: artery morphogenesis	0.01
GO:0060840: artery development	0.012
GO:0016525: neg. regulation of angiogenesis	0.012

**Figure 1:**
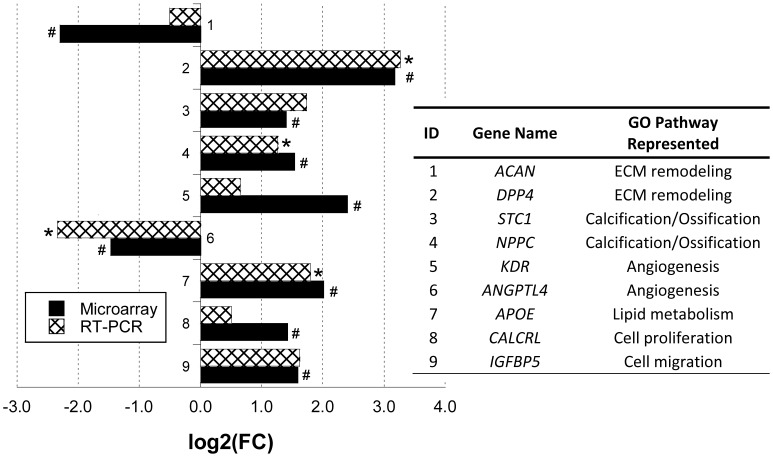
qRT-PCR validation of microarray data. Data are displayed as mean log2(fold-change) in gene expression in male versus female samples. Gene abbreviations: aggrecan (*ACAN*), dipeptidyl-peptidase 4 (*DPP4*), stanniocalcin 1 precursor (*STC1*), natriuretic peptide precursor C (*NPPC*), kinase insert domain receptor (*KDR*), angiopoietin-like 4 (*ANGPTL4*), apolipoprotein E (*APOE*), calcitonin receptor-like (*CALCRL*), and insulin-like growth factor binding protein 5 (*IGFBP5*). *P<0.05 compared to female VIC RT-PCR results, ^#^P<0.05 compared to female VIC microarray results.

Representative genes were selected from each of these main CAVD-relevant GO biological process categories for validation via qRT-PCR, using the same tissue samples used in the microarray (Figure 1), as well as samples from 16 additional animals (Figure S2). For these nine genes, the fold changes obtained via microarray analysis demonstrated substantial agreement with the fold change values determined via qRT-PCR, thereby confirming the microarray data (Figure 1). Moreover, for the majority of these genes, the expression relative to female samples was consistent with an increased genetic propensity for male VICs to adopt a diseased phenotype. Specifically, relative to female valves, males exhibited increased expression of genes related to CAVD-relevant biological processes, namely increased proliferation (*CALCRL*), increased calcification (*STC1, NPPC*), enhanced lipid metabolism (*APOE*), increased ECM remodeling (*DPP4, ACAN*), increased migration (*IGFBP5*), and decreased inhibition of angiogenesis (*ANGPTL4*).

### Functional Pathway and Network Analysis

**Figure 2:**
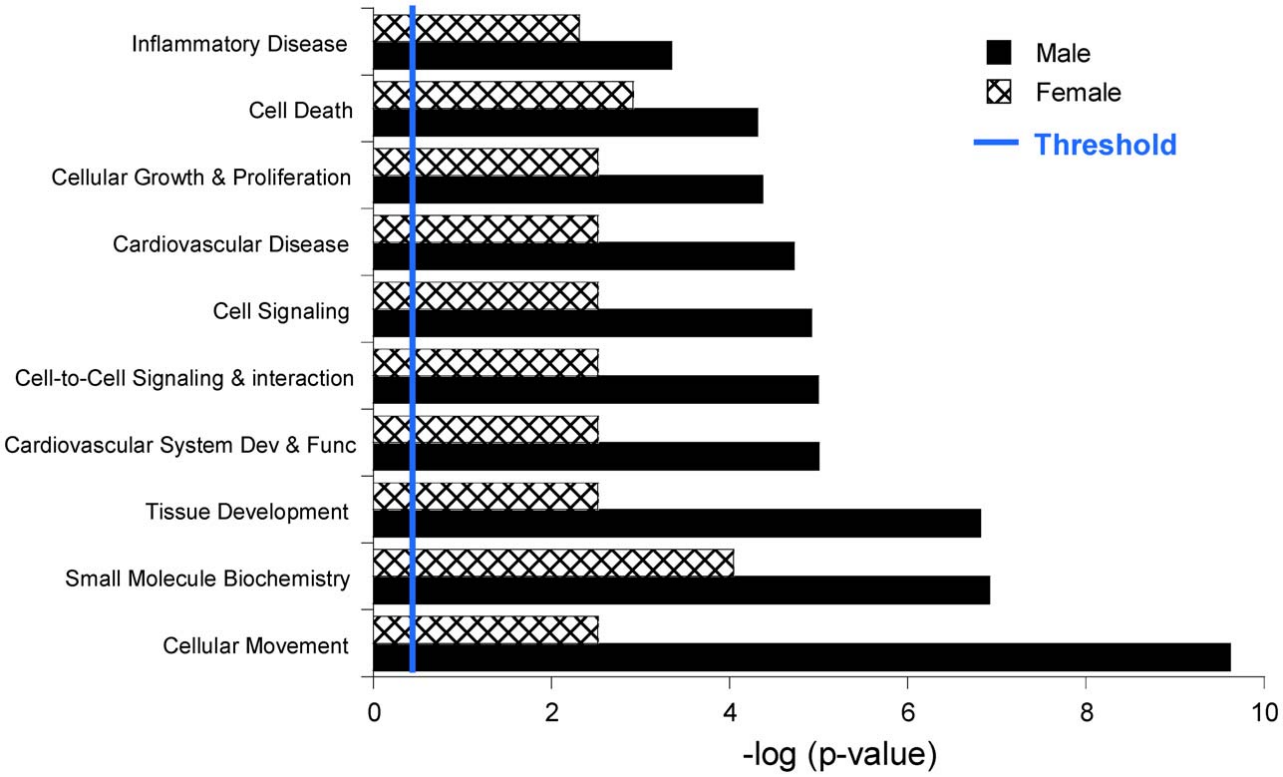
Functional categorization analysis of most significant pathways and diseases represented by microarray data. A functional categorization analysis of the most significant pathways and diseases represented in the microarray-generated list of significantly different genes was generated using Ingenuity software. The p-value was calculated using the right-tailed Fisher’s exact test. Each bar represents the highest level of function for each category, each of which includes many sub-level functions and is represented by the number of genes in the male and female samples (denoted in parentheses, respectively). The bar was determined by the lowest p-value among sub-level functions for each category. Threshold indicates p-value cutoff of 0.05.

IPA functional analysis for pathways and diseases revealed many CAVD-relevant pathways overrepresented in male samples compared to female samples, including cell death, cell proliferation, cell movement, and cell-to-cell signaling and interaction (Figure 2). In addition, cardiovascular and inflammatory disease categories were also identified to be significantly overrepresented in male versus female samples (Figure 2). Each functional pathway or disease category defined by IPA includes sub-level functions, thus the data presented in Figure 2 represent the most significant sub-level function in each category. Cellular movement, small molecular biochemistry, and tissue development were the most significantly represented in male versus female samples out of the dataset.

To investigate the interaction of these differentially regulated genes with other gene products, pathways, and biological processes, molecular networks were formed in IPA using functional relationships between gene products based on known connections in peer-reviewed literature. These networks include genes from the significant genes list and their interactions with genes that are biologically relevant to the pathway but not identified in the microarray. The top 5 networks among the significant genes upregulated in male VICs represent many pathways suspected to be involved in CAVD pathogenesis (Table 2). Each network was identified based on a numerical rank score according to the degree of relevance of the network to the molecules in the significant genes list and based on the hypergeometric distribution calculated as –log(Fisher’s exact test result). A score greater than two indicates a ≥99% confidence that a focus gene network was not generated by chance alone [27]. In these network illustrations, genes or gene products are represented as nodes, and the biological relationship between two nodes is represented as an edge (line). All edges are supported by at least one published reference.

**Table 2:** IPA-generated molecular networks assembled from differentially expressed genes (bold) in males versus females in the significant genes list.

ID	Genes/Molecules in Network	Score‡	Focus Genes	Top Functions Represented
1	Alp, **APOE**, **CD55,** **CD93,** Collagen Alpha1, **DR2**, **ENC1**, **ENTPD1**,**FCER1A**, FSH, hCG, HDL, **IGFBP5**, IL1, **IL17D**, Immunoglobulin, Lh, **LIF**,NFkB (complex), **NPPB**, **NPPC**, **NPR1**, P38 MAPK, **PHLDA1**, **PODXL**, **PPAP2A**,Pro inflammatory Cytokine, **PTPRR**, **RGS5**, **RSAD2**, Serine Protease,**SERPINB2**, **STC1** *, Vegf, **VSNL1**	47	22	Tissue Development, Nucleic Acid Metabolism, Small Molecule Biochemistry
2	Actin, Akt, Alpha Actinin, **CALCRL**, CaMKII, **CD24**, **CDH13**, **CLIC5**,**DPP4**, **ELTD1**, ERK, ERK1/2, **F2RL1**, Gpcr, **HTR2B**, **ICAM2**, Insulin,Integrin, **ITGA6** *, Jnk, **KDR**, Mapk, **MYCN**, Nfat (family), **NPY**, **NRXN1**,PI3K (complex), Ras, **RRAD**, **SFRP2**, **SORBS2**, **TES** *, **TM6SF1**, TSH, **ZNF512B**	38	20	Cardiovascular System Development and Function, Cell Morphology, Organismal Development
3	AFM, APP, **ARHGEF3** *, CAPS4, Caveolin, **CDA**, Ck2, DDX3X, EIF2B1,EIF2S1, FUS, **GABRA1**, GABRA3, **GABRB2**, GABRG1, **GNS**, HNRNPK, HNRNPU,IgG, IL6, **IL17D**, **KHDRBS3**, LDHA, LDLR, **LMO2**, **MALL**, MYC, OSM,**PDCD11**, **ROR1**, **RTN1**, **SERPINB7**, STAT1, TGFB1, **TPBG**	25	14	Gene Expression, Neurological Disease, Organismal Injury and Abnormalities
4	**ABI3**, ABI3BP, **ARHGAP27**, **ARHGEF3** *, **ARHGEF15**, CDH1, **CDH11**, DCC,DLG4, Eif2, EIF2S1, **EIF2S3**, FZD4, GAB2, GDP, GRB2, **ITIH4**, LDHA,**LRRTM4**, **NDP**, NDRG1, PAK1, PIK3R2, PRKAB1, RAC1, RHOA, **RIMS2** *, RPL5,RPL13, **SLC9A3R2**, **SOX13**, TAC1, **TPPP3**, TSPAN12, WNT1	24	13	Cellular Movement, Cell Signaling, Molecular Transport
5	**C2orf40**, CCND1, CTNNβ-LEF1, **DDX3Y**, DIO2, DOOCK7, **ESAM**, **ETF1**,**FRY**, Groucho, **KLHL13**, L-triiodothyronine, LDLR, **LEF1**, **MARK1**, **MFAP5**,NOTCH1, PTH, RAB3B, RBX1, SHBG, SNCA, TCF/LEF, TLE1, TLE2, TLE4, **TPPP**,UCP3, **UTY**, VENTX, WNT1, YWHAG, ZEB2	19	11	Cellular Assembly and Organization, Tissue Development, Cell Cycle

‡ The score is a numerical rank of the degree of relevance of the network to the molecules in the significant genes list and is based on the hypergeometric distribution calculated as –log(Fisher’s exact test result).

* indicates multiple identifiers in the significant genes dataset map to a single gene in the IPA Global Molecular Network.

**Figure 3:**
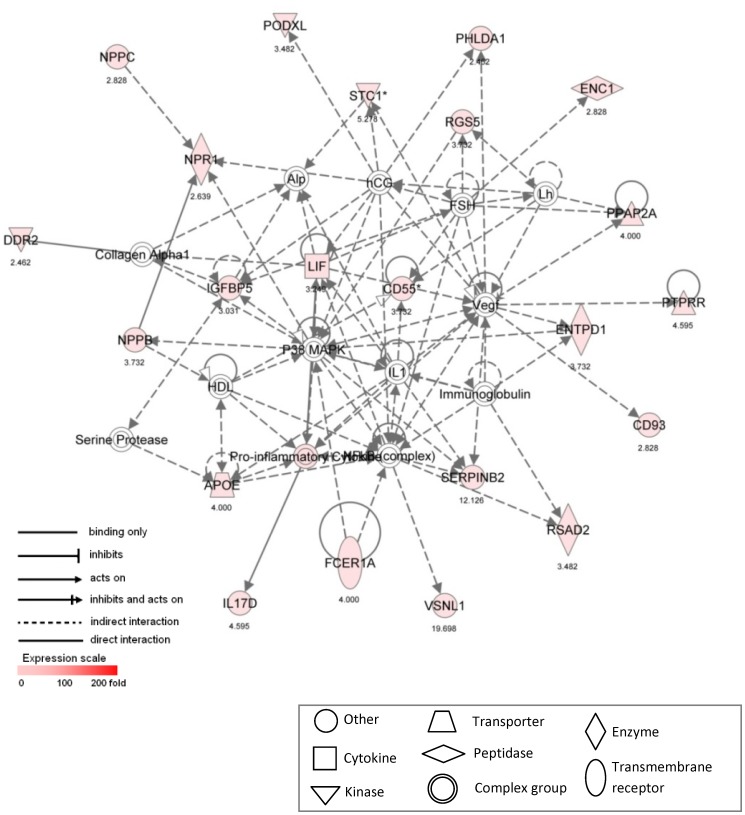
Ingenuity pathway analysis Network #1. Ingenuity pathway analysis was used to assemble a network based upon 22 differentially expressed focus genes that were upregulated in male VICs compared to female VICs. Genes are represented as nodes, with node shape representing the functional class of the gene product (seen in legend). Node color depicts degree of overrepresentation in male samples; uncolored nodes are depicted based upon evidence in the IPA Knowledge Base indicating a strong biological relevance to the network. *indicates multiple identifiers in the dataset file map to a single gene in the IPA Global Molecular Network. Corresponding fold change values are listed beneath each gene label. Focus gene names are defined in Table S1.

Network #1 (Figure 3) received the highest score (47) and is assembled around 22 focus genes that were upregulated in male VICs. This network is not centered upon any single gene or process, but includes several genes and gene products that are highly relevant to multiple CAVD-related phenomena. In particular, Network #1 contains genes and molecules known to be involved in inflammation (IL1, IL17D, CD55, CD93, FCER1A, Immunoglobulin, NF-κB complex), ossification (DDR2, RSAD2, STC1), angiogenesis (VEGF), valve cell differentiation (NPPC), and lipid metabolism (APOE, PPAP2A) [16], [28]–[30]. Vascular endothelial growth factor (VEGF), which plays a central role in angiogenic processes, acts as one of the hubs in this network, indirectly interacting with genes and gene products whose GO biological process functions are primarily associated with apoptosis (PHLDA1), lipid metabolism (PPAP2A), ossification (STC1), and inflammation (CD55, IL1, CD93, Immunoglobulin, NF-κB complex).

**Figure 4:**
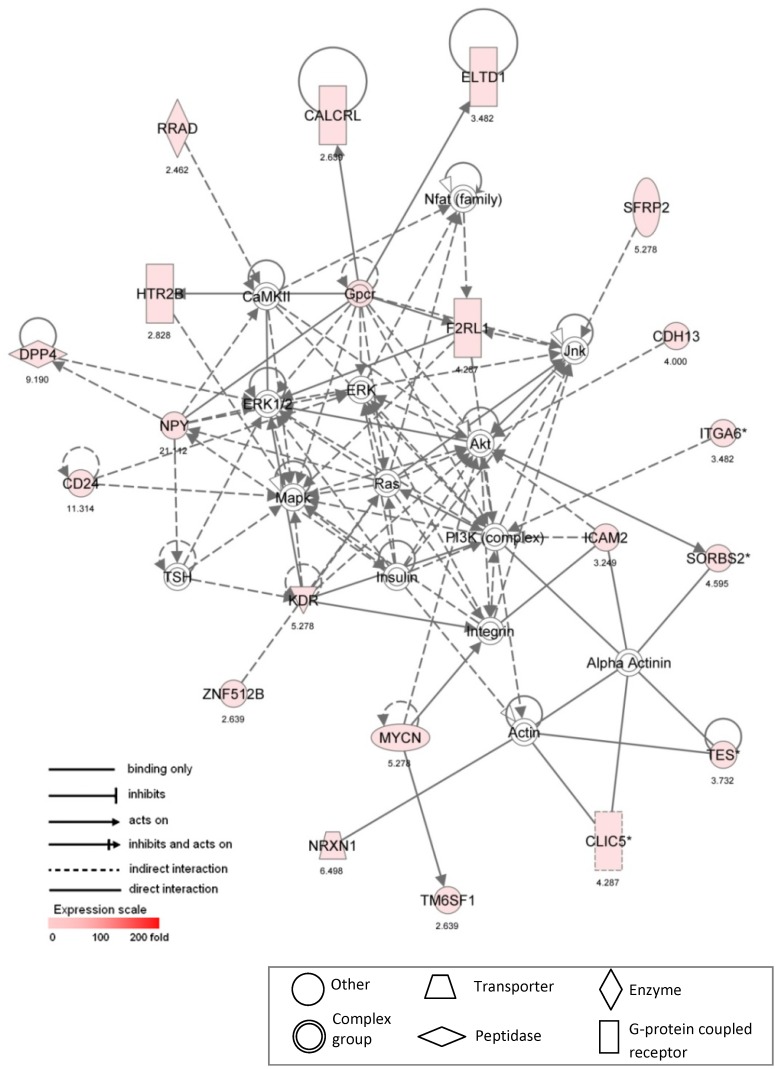
Ingenuity pathway analysis Network #2. Ingenuity pathway analysis was used to assemble a network based upon 20 differentially expressed focus genes that were upregulated in male VICs compared to female VICs. * indicates multiple identifiers in the dataset file map to a single gene in the IPA Global Molecular Network. Corresponding fold change values are listed beneath each gene label. Focus gene names are defined in Table S1.

Network #2 (Figure 4, score 38) was assembled from 20 focus genes that were upregulated in male VICs and is centered on the mitogen-activated protein kinase and extracellular-regulated kinase (MAPK/ERK) system, a known pathway involved in CAVD [25]. This network also includes several receptors involved in G-protein coupled signaling, and notably contains HTR2B (5-hydroxytryptamine (serotonin) receptor 2B); increased 5-HT levels and 5-HT receptor signaling are known to induce thickening and fibrosis of the valve and ECM reorganization [31]–[33], with these effects mediated by 5-HT-induced increases in TGF-β1 production [31]. The signaling pathways represented in Network #2 also connect to several genes whose primary biological process functions are associated with angiogenesis (KDR, SFRP2, NRXN1).

**Figure 5:**
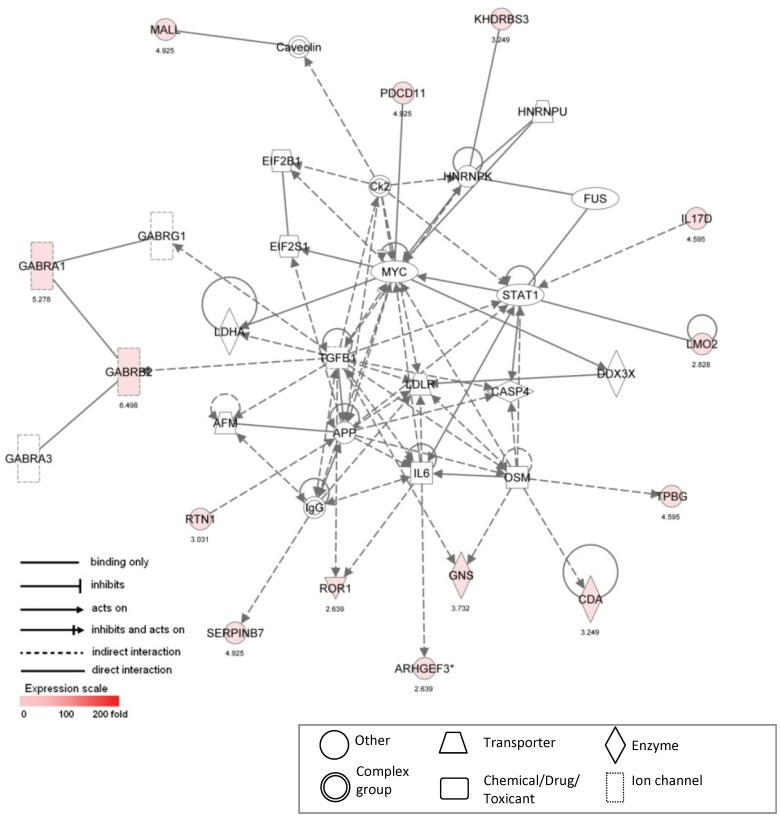
Ingenuity pathway analysis Network #3. Ingenuity pathway analysis was used to assemble a network based upon 14 differentially expressed focus genes that were upregulated in male VICs compared to female VICs. Corresponding fold change values are listed beneath each gene label. Focus gene names are defined in Table S1.

Network #3 (Figure 5, score 25) was assembled from 14 genes that were upregulated in male VICs and centers on TGF-β1, a cytokine well known to be involved in CAVD [13], [15], [34], and low density lipoprotein receptor (LDLR), which is involved in lipid metabolism. This network also contains several genes related to apoptosis (CASP4, DSM, ARHGEF3), a process which is also upregulated in CAVD and *in vitro* VIC calcification [15], [25], [35]. Finally, networks #4 and #5 (Table 2) reveal disease-related molecules associated with the dataset that include Rac1/RhoA (Figure S3, score 24, 13 genes) which is involved in valvular cell contractility and activation [35], and Notch1 and cyclin D (CCND1) (Figure S4, score 19, 11 genes) which are involved in proliferation processes and thus logical players in CAVD pathogenesis [36].

### In vitro Culture of Sex-separated VIC Populations

**Figure 6:**
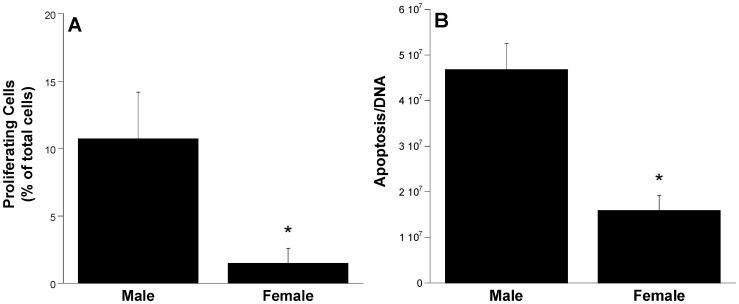
Translation of findings to *in vitro* VIC function. Male and female VICs were cultured for 5 days in serum-free media and evaluated for (A) proliferation, using the Click-iT EdU Alexa Fluor 488 Imaging Assay, and (B) apoptosis, using the Promega Caspase-Glo 3/7 Assay System. Values are means plus standard deviation, n = 4 per condition. *P<0.05 compared to male VICs.

In order to evaluate whether the gene expression differences identified in male and female explanted leaflets translated into measurable differences in VIC function upon *in vitro* culture, a preliminary validation experiment focused upon quantification of proliferation and apoptosis in VIC cultures. As noted earlier, increased proliferation and apoptosis are both observed in diseased valves [15], [34]. After separating excised leaflets by sex and culturing the harvested VICs for 5 days *in vitro*, male VICs were found to have significantly higher levels of proliferation (Figure 6A, P<0.05) and apoptosis (Figure 6B, P<0.05) than female VICs.

## Discussion

While the pathogenesis of CAVD remains incompletely defined, there is evidence that it is a multifaceted process involving a combination of genetic and systemic factors that combine to initiate disease [18], [19]. A previous analysis of human diseased valve explants compared to healthy controls revealed many upregulated pathological pathways that separate CAVD from a non-diseased state on the cellular scale [17], [18], [22]. Because male sex remains a relatively unexplained, but significant, risk factor for CAVD, the present work investigated the existence of intrinsic, cellular-scale differences that may play a role in genetically predisposing male valves to CAVD. As noted in a subsequent section, this investigation primarily examined sex-related differences that are not likely to be due to differences in sex hormone profiles.

This study provides the first comparison of global expression profiles between healthy male and female VICs and reveals several findings that suggest many CAVD-related molecules and processes may be intrinsically active or primed to be activated in male VICs compared to female VICs. Interestingly, many of the same expression patterns and characteristics found in human CAVD explants were also identified in our dataset analysis of healthy male and female pigs. In particular, some of the pathways and processes that were previously identified as different between healthy and diseased human valves [22] include angiogenesis, lipid management and deposition, CNP, MAPK/ERK-1/2 pathways, and inflammation; these pathways and processes were also identified in the current work as being different between healthy male and female porcine valves, and are discussed in further detail below. Overall, this pathway analysis enabled the visualization of potentially important connections and interactions that may coordinate to regulate sex-dependent CAVD processes and provides a molecular framework from which to further study these phenomena.

Importantly, this work also finds that these differences in gene expression have significant functional consequences with respect to cell behavior. We chose to evaluate two basic, CAVD-relevant cell functions – proliferation and apoptosis – in a preliminary *in vitro* comparison between male and female VICs in culture. These findings not only illustrated the existence of significant differences in the behavior of male versus female VICs, but also confirmed microarray results that suggested greater expression of disease-related behaviors in male VIC populations. This result has direct implications for *in vitro* studies of CAVD, which are commonly performed using mixed-sex cell populations. The authors are not aware of any other studies that have cultured VICs following separation by sex.

### Angiogenesis

In valve leaflets, VICs can activate endothelial cells to produce VEGF by increasing expression of VEGF receptors [16]. Specifically, kinase insert domain receptor (*KDR*), also referred to as vascular endothelial growth factor receptor 2 (*VEGFR-2*), was identified in previous studies to be upregulated in human CAVD explants when compared to healthy control valves [22], [23]. In the present work, *KDR*, along with two other genes whose products are known to promote angiogenesis (*SFRP2* and *NRXN1*), were identified as being upregulated by over 5-fold in male samples compared to female samples (Figure 4 and Table S2). Increased angiogenesis potential in male valves is further supported by the identification of *VEGF* in Network #1 (Table 2 and Figure 3), and the distinct downregulation of the angiogenesis modulator angiopoietin-related protein 4 (*ANGPTL4*) in male samples compared to female samples (Figure 1) [37]. These findings suggest that factors associated with initiating angiogenesis in CAVD may be more prominent in healthy male samples when compared to female samples.

### Lipid Deposition

In the significant genes list presented in this study (Table S2) *APOE,* a key player in blood cholesterol management, was upregulated by 2-fold in healthy male samples compared to female samples (Figure 1). Our findings suggest that male valves have a higher baseline *APOE* expression level that may assist in the initiation of inflammation cascade events, including MMP and TGF-β1 expression [2], [19]. These results are again consistent with observations of healthy vs. diseased human valves, where APOE expression is upregulated in diseased valve leaflets [2], [22]. Additionally, the LDL receptor (*LDLR*) gene was identified in Networks #3 and #5 as being a molecule involved in pathways overrepresented in the male VIC population (Table 2). This finding may signify heightened involvement of LDL/LDLR signaling within male valves versus female valves, indicating that healthy male VICs may be more active in lipid management and deposition when compared to female VICs.

### Mitogen-activated Protein Kinase Pathway

As discussed earlier, ERK phosphorylation is significantly increased in diseased human valves and calcifying porcine VIC cultures when compared against healthy valves and cultures [24], [25], and inhibition of ERK is sufficient to inhibit numerous disease-related behaviors in porcine VIC cultures [25]. Thus, it is interesting to find that a gene network centered around MAPK/ERK was found to be overrepresented in male VICs compared to female VICs (Network #2, Table 2, Figure 4). This finding may indicate an increased propensity for MAPK/ERK activation within male VICs, which may compound with other disease-inducing factors revealed in this study to produce an overall increased susceptibility in male VICs to becoming activated to diseased phenotypes and participating in calcification.

### Inflammation

Several interleukins and pro-inflammatory cytokines that are related to overrepresented genes in male versus female VICs were identified in this study (Table 2). These include interleukins -1, -6, and -17D, and TGF-β1 (Network #3, Table 2, and Figure 5). Diseased leaflets are known to contain increased levels of both IL-1 and TGF-β1, and studies have found that IL-1-receptor antagonist-mediated mechanisms of anti-inflammation are dysfunctional in diseased valves [15], [38]. As an inflammatory cytokine, TGF-β1 is normally released in response to injury as part of the inflammatory cascade, and is a major inducer of the calcification and differentiation of VICs into myofibroblast-like cells [13], [15]. TGF-β1 is also associated with increased ECM production and remodeling and induces proliferation of VICs at locations of injury [13], [39]. Thus, these inflammation gene expression findings are again consistent with male sex being associated with increased expression of CAVD-related genes. In combination with increased molecular activity involving pro-inflammatory cytokines IL-17D and IL-6, it is possible that the genes significantly expressed in male VICs compared to female VICs may render the male valves more prone to involvement in interleukin-mediated inflammatory processes and disease-inducing inflammation. Such differences in inflammation can also significantly influence other cellular processes such as proliferation, migration, and recruitment of additional inflammatory cells [16], all basic functions that are known to be upregulated in diseased valves compared to non-calcified valves [22].

### Limitations

Sex-related disparities in the incidence and progression of CAVD are likely due to a complex, intertwined network of factors, with cellular scale differences comprising only one part of this picture. The results presented here indicate that many gene expression pathways that correlate with known pathological mechanisms in CAVD are overrepresented in healthy male versus female aortic valve leaflets. Because the samples used in this study were taken from healthy valves, it can be hypothesized that the clinical risk factor of male sex may originate from an increased genetic propensity to developing CAVD on the cellular scale. More studies are warranted to better understand the importance of these basal gene expression differences, but it appears that the pathogenesis of the disease may not be identical between males and females. Although confirmation in human valves will be needed to fully realize the clinical implications of this work, our findings do motivate an immediate and significant change in the manner in which *in vitro* VIC studies are executed. Specifically, our results strongly support the use of sex-separated VIC cultures for *in vitro* research in valve biology and CAVD, representing a significant shift of the standard techniques employed in this field.

This work does use tissues from castrated male pigs, as all male pigs obtained from U.S. slaughterhouses are routinely castrated at a young age to preserve meat quality, thus creating a limitation of this work with respect to fully describing the human condition. However, while the hormonal profile of a castrated male does not resemble that of male humans, the use of castrated pigs actually has several positive consequences as well. First, the use of castrated pigs makes this work directly applicable to all researchers currently using porcine tissues for their *in vivo* or *in vitro* research, as all male pigs used by these researchers thus far have been castrated. As noted above, one of the major implications of this work is that we recommend ceasing the standard practice of using mixed-sex VIC cultures; it is the fact that we are using the same type of animals as other VIC researchers that enables us to make this recommendation. Unpublished work from our lab has also found that VICs do not appear to be directly responsive to sex hormones, or express common sex hormone receptors. Lastly, and quite importantly, use of castrated males actually provides us with a unique opportunity to separate out the intrinsic differences in VIC behavior from the sex hormone-induced differences, as castrated males are similar to females in hormone profiles at the time of slaughter. Our data suggest the existence of sex-related CAVD differences that are unlikely to be directly tied to sex hormone profiles, a result which is quite intriguing, and likely to be highly useful in the eventual interpretation of human valve gene data.

Finally, as noted above, confirmation of these differences, as well as investigation of sex hormone-related phenomena in human samples, will be needed to verify cellular-scale sex-related differences in human CAVD. The consistency of our findings with existing analyses of diseased human valves, however, is suggestive of the porcine model serving as an appropriate representative of human valve biology. Moreover, if these porcine findings are not borne out by an analysis of human valves, then the field may be facing a much more significant problem, as pigs are the most common source of cells used to perform CAVD research.

## Materials and Methods

All chemicals were obtained from Sigma-Aldrich, St. Louis, MO unless otherwise noted.

### Ethics Statement

All tissues used in this study were acquired post-mortem from a commercial slaughterhouse, and were therefore not subject to institutional animal protocol approval. The slaughterhouse follows USDA and Humane Slaughter Act guidelines for care and slaughter of the swine.

### RNA Isolation

Aortic valve leaflets were surgically removed using sterile techniques from hearts obtained from 5 month old female and castrated male pigs (Hoesly’s Meats, New Glarus, WI) weighing approximately 90 kg, and the endothelial cells were manually denuded with a scalpel (Figure S1 and methods). After homogenization of the leaflets, total RNA was isolated using the RNeasy fibrous tissue spin-column kit (Qiagen, Valencia, CA) according to the manufacturer’s instructions.

### Microarray Hybridization

RNA samples from three male and three female pigs were selected for microarray hybridization analysis based on sample purity and quality control analysis. Affymetrix GeneChip Porcine Genome Arrays (Affymetrix, Santa Clara, CA) containing 23,937 probesets that interrogate 23,256 transcripts from 20,201 *Sus scrofa* genes were selected for use in this study. The arrays were processed at the University of Wisconsin-Madison Gene Expression Center (Madison, WI) following all appropriate manufacturer’s instructions. Post-processing of the Porcine GeneChips was performed on the AFX 450 Fluidics Station according to all AFX protocols and procedures defined for the porcine array and scanned on a GC3000 G7 scanner. Data were extracted from images using the AFX Expression Console v1.1 software.

### Microarray Data Analysis

The microarray data were processed using the open source statistical language R v2.12.0 and the libraries included in the Bioconductor Project [40]. Raw expression values from *.CEL files were background-corrected and normalized using the Robust Multi-array Analysis (RMA) method [41], and filtered based on the CV (standard deviation divided by the mean). The mean expression of each probe set in the male samples was then compared with that of the female samples. The differential gene expression between male and female samples was determined and a significant genes list was generated using the Empirical Bayes *t*-test statistic from the *limma* package [42], where the significance threshold was set to a false discovery rate (FDR) of 0.05 and a minimum fold change of 2. Gene ontology (GO) enrichment analysis was performed on the significant genes list using the GOstats package [43]. The significant genes list was annotated using the chip annotations provided by both Affymetrix (NetAffx) and Tsai et al. [44]. The data discussed in this publication comply with MIAME standards and have been deposited in NCBI’s Gene Expression Omnibus, accessible through GEO Series accession number GSE33654 (http://www.ncbi.nlm.nih.gov/geo/query/acc.cgi?acc=GSE33654).

Extended pathway analysis was performed on the significant genes list using Ingenuity Pathway Analysis (IPA) Software v9.0 and services (Ingenuity Systems, Redwood City, CA). The significant genes list was divided into genes upregulated in either male or female samples and functional analysis was performed on each list in IPA. A p value was calculated for each gene group mapped to certain functions using the Fisher’s exact right-tailed test to determine the probability that the association between the genes and each biological function was not explained by chance alone. In addition, significant genes from the dataset that met filter criteria and had at least one published instance linking it to one other molecule in the Ingenuity Knowledge Base were named focus genes to form a global molecular network. Other genes that were not from the dataset but were necessary for network formation were identified by IPA and supplemented based on their connectivity as defined by literature references. Networks were graphically depicted for visualization of gene relationships.

### Validation of Microarray Via qRT-PCR

The differential expression of a group of genes identified by microarray analysis was validated by quantitative real-time quantitative polymerase chain reaction (qRT-PCR) using the same six animal samples used in the microarray, and with an additional 8 samples per sex (total of n = 11 for each sex) to test for consistency in a larger animal pool. Genes that were selected for validation appeared prominently in several biological processes within the larger process categories. The corresponding GO Pathways for each gene were selected according to what is believed to be the major role of the gene in the dataset. RNA was reversed transcribed using a High Capacity cDNA Reverse Transcription kit (Applied Biosystems, Carlsbad, CA). qRT-PCR amplification was performed using TaqMan Gene Expression Assays (Applied Biosystems). Target genes were normalized to GAPDH, and Ct values were analyzed using the comparative ΔΔCt method relative to female samples [45]. Statistical analysis of qRT-PCR data was performed using one-way analysis of variance (ANOVA) with Tukey’s HSD post-test. P-values less than or equal to 0.05 were considered statistically significant.

### In vitro Culture and Analysis of Sex-separated VIC Populations

VICs were isolated from female and male porcine aortic valves within 5 hours of death using the collagenase digestion protocol described previously [46]. Passage 2 VICs were seeded in 24-well tissue culture polystyrene (TCPS) plates at a density of 50,000 cells/cm^2^ and refed every 48 hours. Male and female VIC populations were verified using Taqman-based polymerase chain reaction (PCR) for the *SRY* gene present on Y-chromosomes following methods described in an earlier section.

Proliferation was quantified using the Click-iT EdU Alexa Fluor 488 Imaging Assay (Invitrogen, Carlsbad, CA) and normalized to 4′,6-diamidino-2-phenylindole (DAPI) fluorescence. Briefly, cells were seeded in media supplemented with 1% charcoal/dextran treated FBS and then refed thereafter in serum-free medium. The EdU incubation and Click-iT reaction were carried out according to manufacturer instructions. Positively-stained (proliferating) cells were counted using ImageJ software (NIH) with fluorescent microscope images (Olympus IX51, Olympus, Center Valley, PA) and normalized to the number of total cells indicated by DAPI fluorescence.

Apoptosis was quantified on day 5 of culture using the Caspase-Glo 3/7 Assay System (Promega, Madison, WI) per manufacturer’s instructions and normalized to DNA measured using the PicoGreen dsDNA assay (Invitrogen, Carlsbad, CA) in each culture well.

An expanded Methods section is available in Data Supplement Text S1.

## Supporting Information

Figure S1 **Verification of valvular endothelial denuding method via immunohistochemical detection of von Willebrand factor, a marker for endothelial cells.** (A) Native leaflet section without endothelial cell denuding, (B) Leaflet section after endothelial cell scalpel denuding. Von Willebrand factor (green), DAPI (blue), 4× magnification, scale bar  = 100 µm.
(TIF)

Figure S2 **Additional** **qRT-PCR validation of microarray data.** Data are displayed as mean log2(fold-change) in gene expression in male versus female samples for microarray results and for RT-PCR of additional animal samples (N = 11 per sex). Gene abbreviations: aggrecan (*ACAN*), dipeptidyl-peptidase 4 (*DPP4*), stanniocalcin 1 precursor (*STC1*), natriuretic peptide precursor C (*NPPC*), kinase insert domain receptor (*KDR*), angiopoietin-like 4 (*ANGPTL4*), apolipoprotein E (*APOE*), calcitonin receptor-like (*CALCRL*), and insulin-like growth factor binding protein 5 (*IGFBP5*). *P<0.05 compared to female VIC RT-PCR results, ^#^P<0.05 compared to female VIC microarray results.
(TIF)

Figure S3 **Ingenuity pathway analysis Network #4.** Ingenuity pathway analysis was used to assemble a network based upon 13 differentially expressed focus genes and gene products that were upregulated in male VICs compared to female VICs. IPA categorizes the main functions of this network as cellular movement, cell signaling, and molecular transport. Corresponding fold change values are listed beneath each label.
(TIF)

Figure S4 **Ingenuity pathway analysis Network #5.** Ingenuity pathway analysis was used to assemble a network based upon 11 differentially expressed focus genes and gene products that were upregulated in male VICs compared to female VICs. IPA categorizes the main functions of this network as cellular assembly and organization, tissue development, and cell cycle. Corresponding fold change values are listed beneath each gene label.
(TIF)

Table S1 **Summary of (standard) gene abbreviations used within the manuscript.**
(DOC)

Table S2 **Microarray genes found to be significantly different in male versus female samples.** LogFC refers to the log2-based fold change between the sexes. t is the moderated t-statistic from the empirical Bayes methods. P value is the raw p value and the adj. P value is the p value adjusted for multiple comparisons using the Benjamini and Hochberg’s false discovery rate. B is log-odds that the gene is differentially expressed.
(DOC)

Table S3 **Significant biological processes determined by GO enrichment analysis.** Expected count (Exp Count) refers to the number of differentially expressed genes that are predicted within the GO term tested, Count refers to the number of differentially expressed genes that were found within the GO term tested, and Size refers to the number of genes in the microarray that are listed within the GO term tested.
(DOC)

Table S4 **Summary of significant biological processes determined by GO enrichment analysis, categorized according to disease-related pathway grouping.** Expected count (Exp Count) refers to the number of differentially expressed genes that are predicted within the GO term tested, Count refers to the number of differentially expressed genes that were found within the GO term tested, and Size refers to the number of genes in the microarray that are listed within the GO term tested.
(DOC)

Data Supplement Text S1 **Expanded description of Materials and Methods.**
(DOC)
