# Supplementary figures and images for: Sex-Related Differences in Gene Expression by Porcine Aortic Valvular Interstitial Cells
Source: PLoS One. 2012 Jul 10;7(7):e39980. doi: 10.1371/journal.pone.0039980 (PMC3393722; doi:10.1371/journal.pone.0039980)

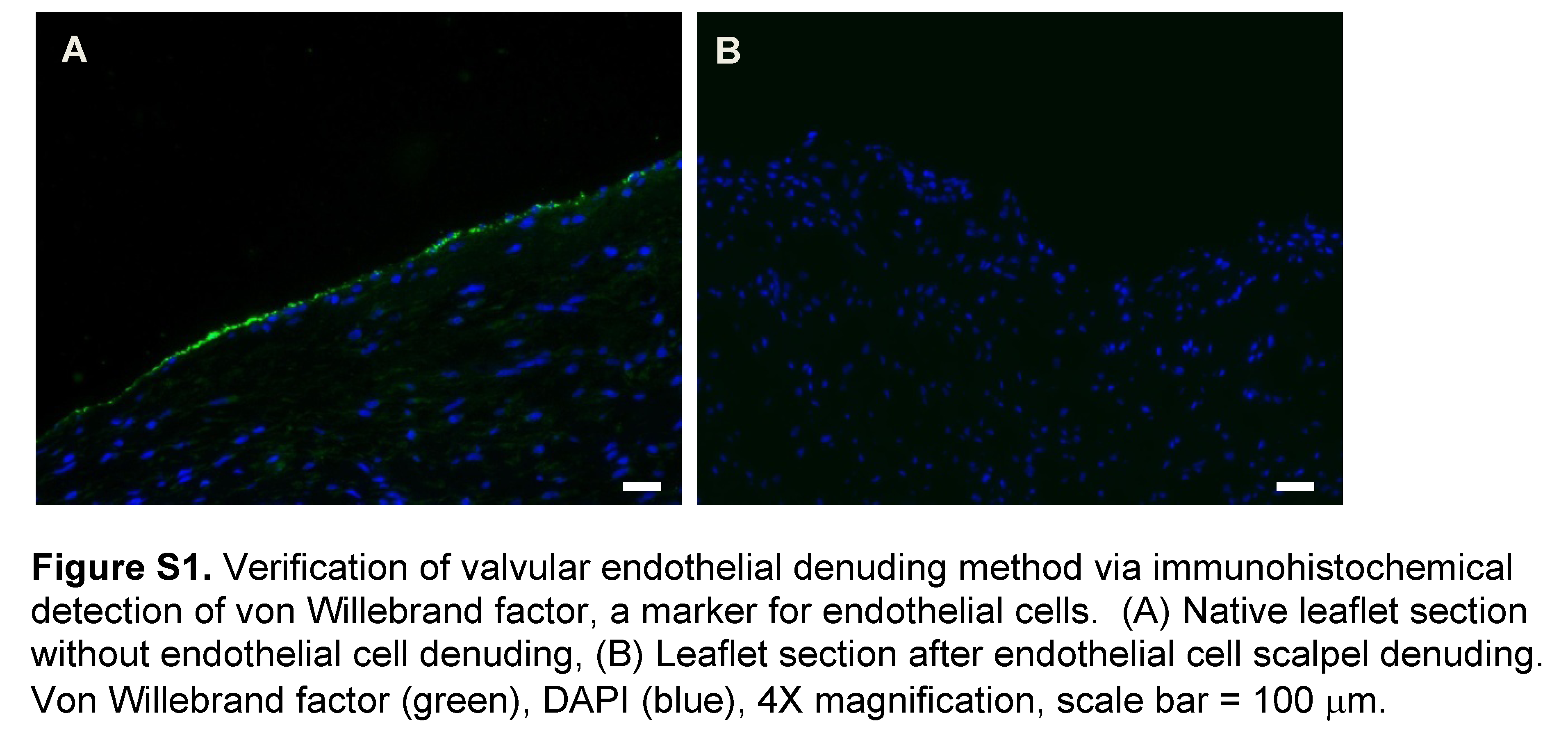

Supplement: Figure S1 — Verification of valvular endothelial denuding method via immunohistochemical detection of von Willebrand factor, a marker for endothelial cells. (A) Native leaflet section without endothelial cell denuding, (B) Leaflet section after endothelial cell scalpel denuding. Von Willebrand factor (green), DAPI (blue), 4× magnification, scale bar = 100 µm. (TIF) [file pone.0039980.s001.tif]

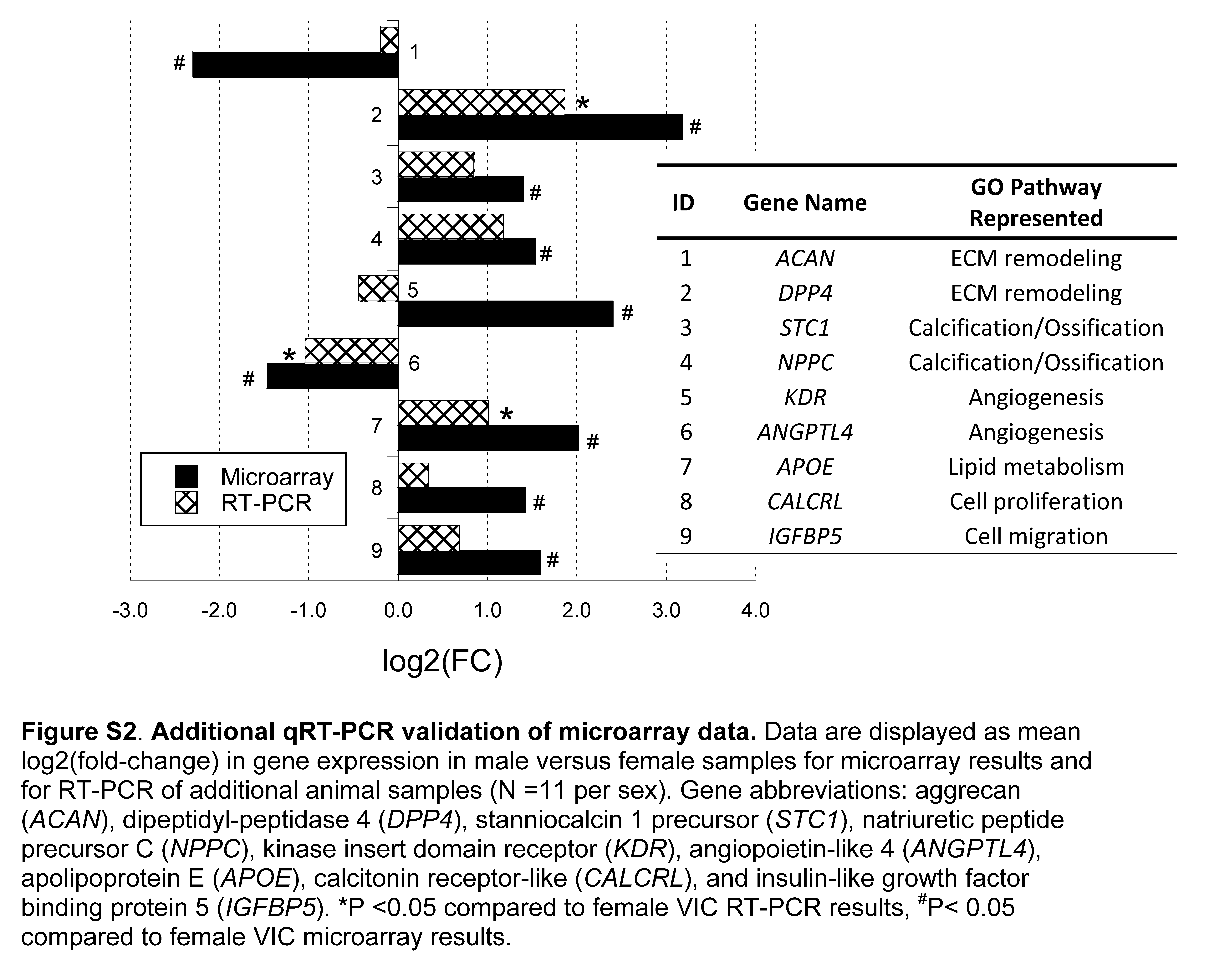

Supplement: Figure S2 — Additional qRT-PCR validation of microarray data. Data are displayed as mean log2(fold-change) in gene expression in male versus female samples for microarray results and for RT-PCR of additional animal samples (N = 11 per sex). Gene abbreviations: aggrecan (ACAN), dipeptidyl-peptidase 4 (DPP4), stanniocalcin 1 precursor (STC1), natriuretic peptide precursor C (NPPC), kinase insert domain receptor (KDR), angiopoietin-like 4 (ANGPTL4), apolipoprotein E (APOE), calcitonin receptor-like (CALCRL), and insulin-like growth factor binding protein 5 (IGFBP5). *P<0.05 compared to female VIC RT-PCR results, #P<0.05 compared to female VIC microarray results. (TIF) [file pone.0039980.s002.tif]

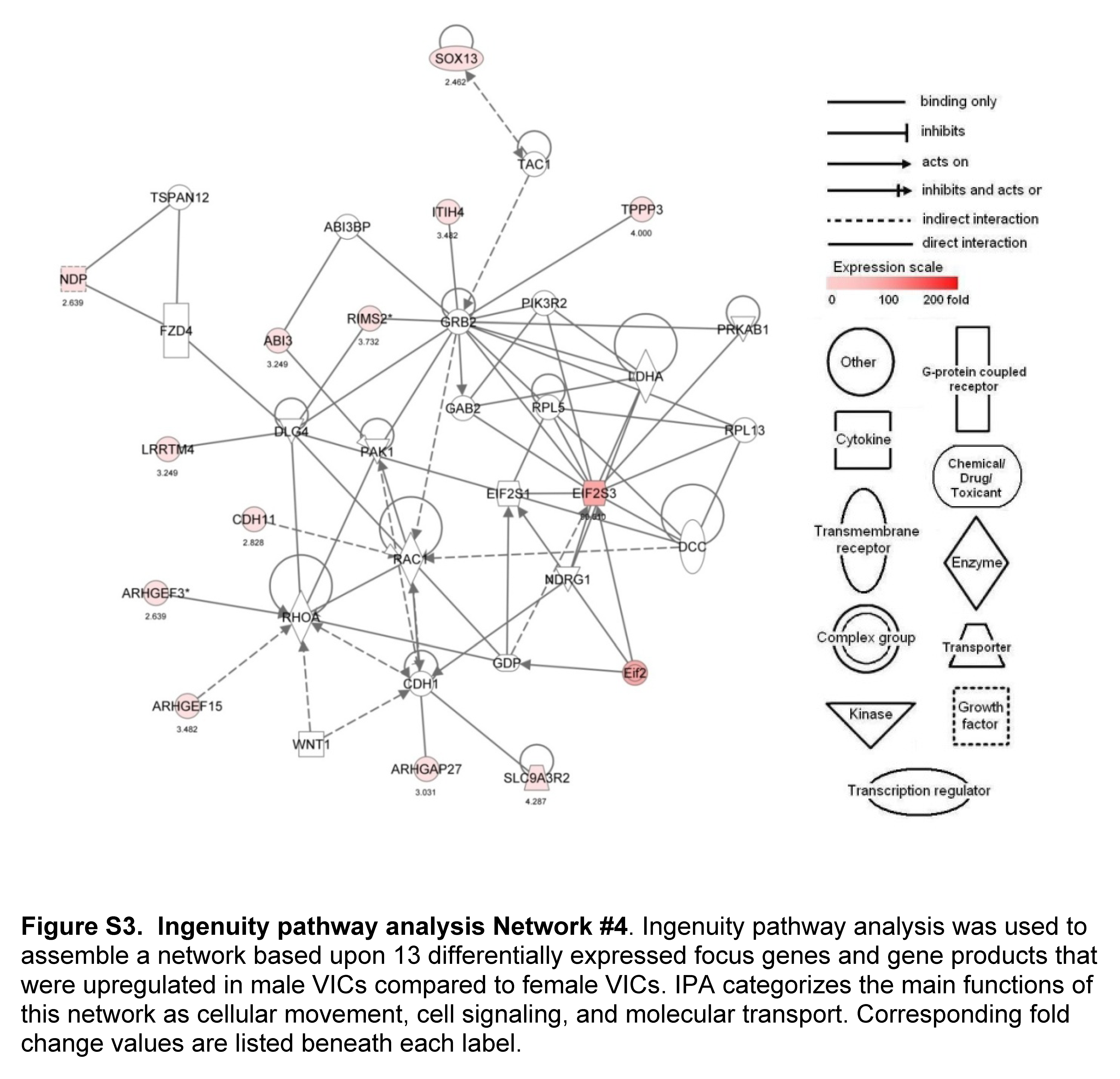

Supplement: Figure S3 — Ingenuity pathway analysis Network #4. Ingenuity pathway analysis was used to assemble a network based upon 13 differentially expressed focus genes and gene products that were upregulated in male VICs compared to female VICs. IPA categorizes the main functions of this network as cellular movement, cell signaling, and molecular transport. Corresponding fold change values are listed beneath each label. (TIF) [file pone.0039980.s003.tif]

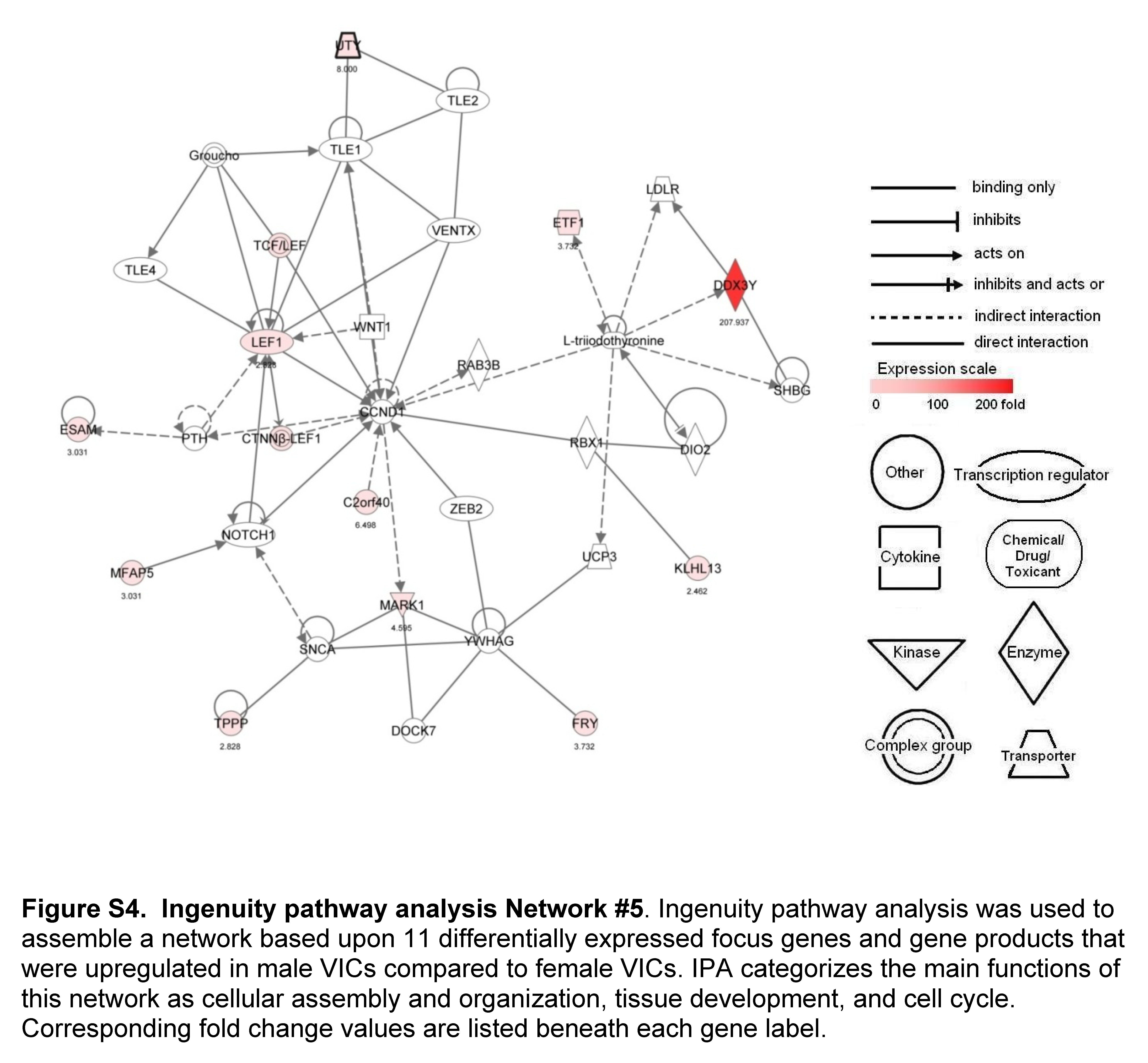

Supplement: Figure S4 — Ingenuity pathway analysis Network #5. Ingenuity pathway analysis was used to assemble a network based upon 11 differentially expressed focus genes and gene products that were upregulated in male VICs compared to female VICs. IPA categorizes the main functions of this network as cellular assembly and organization, tissue development, and cell cycle. Corresponding fold change values are listed beneath each gene label. (TIF) [file pone.0039980.s004.tif]
