# Supplementary material for: Sex-Related Differences in Gene Expression by Porcine Aortic Valvular Interstitial Cells
Source: PLoS One. 2012 Jul 10;7(7):e39980. doi: 10.1371/journal.pone.0039980 (PMC3393722; doi:10.1371/journal.pone.0039980)
Supplement: Table S1 — Summary of (standard) gene abbreviations used within the manuscript. (DOC) [file pone.0039980.s005.doc]

**Table S1.** Summary of (standard) gene abbreviations used within the manuscript.

| Abbreviation | | Gene |
| --- | --- | --- |
| ***ANGPTL4*** | angiopoietin-related protein 4 | |
| ***APOE*** | apolipoprotein E | |
| ***ARHGEF15*** | Rho guanine nucleotide exchange factor (GEF) 15 | |
| ***ARHGEF3*** | Rho guanine nucleotide exchange factor 3 | |
| ***CALCRL*** | calcitonin receptor-like | |
| ***C2orf40*** | chromosome 2 open reading frame 40 | |
| ***CCND1*** | cyclin D1 | |
| ***CD24*** | cluster of differentiation 24 | |
| ***CD55*** | decay accelerating factor for complement | |
| ***CD93*** | cluster of differentiation 93 | |
| ***CDA*** | cytidine deaminase | |
| ***CDH11*** | cadherin 11, type 2, OB-cadherin (osteoblast) | |
| ***CDH13*** | cadherin 13, H-cadherin | |
| ***CLIC5*** | chloride intracellular channel 5 | |
| ***CNP*** | C-type natriuretic peptide | |
| ***DAPI*** | 4',6-diamidino-2-phenylindole | |
| ***DDR2*** | discoidin domain receptor tyrosine kinase 2 | |
| ***DDX3Y*** | DEAD (Asp-Glu-Ala-Asp) box polypeptide 3, Y-linked | |
| ***DPP4*** | dipeptidyl-peptidase 4 | |
| ***EIF2S3*** | eukaryotic translation initiation factor 2, subunit 3 gamma, 52kDa | |
| ***ELTD1*** | EGF, latrophilin and seven transmembrane domain containing 1 | |
| ***ENC1*** | ectodermal-neural cortex 1 | |
| ***ENTPD1*** | ectonucleoside triphosphate diphosphohydrolase 1 | |
| ***ERK*** | extracellular-regulated kinase | |
| ***ESAM*** | endothelial cell adhesion molecule | |
| ***ETF1*** | eukaryotic translation termination factor 1 | |
| ***F2RL1*** | coagulation factor II (thrombin) receptor-like 1 | |
| ***FCER1A*** | Fc fragment of IgE, high affinity I, receptor for; alpha polypeptide | |
| ***FRY*** | furry homolog (Drosophila) | |
| ***GABRA1*** | gamma-aminobutyric acid A receptor, alpha 1 | |
| ***GABRB2*** | gamma-aminobutyric acid A receptor, beta 2 | |
| ***GNS*** | glucosamine (N-acetyl)-6-sulfatase | |
| ***HTR2B*** | 5-hydroxytryptamine (serotonin) receptor 2B | |
| ***ICAM2*** | intercellular adhesion molecule 2 | |
| ***IGFBP5*** | insulin-like growth factor binding protein 5 | |
| ***IL-1*** | interleukin-1 | |
| ***IL-17D*** | interleukin-17D | |
| ***IL-6*** | interleukin-6 | |
| ***ITGA6*** | integrin, alpha 6 | |
| ***ITIH4*** | inter-alpha (globulin) inhibitor H4 (plasma Kallikrein-sensitive glycoprotein) | |
| ***KDR*** | Kinase insert domain receptor | |
| ***KHDRBS3*** | KH domain containing, RNA binding, signal transduction associated 3 | |
| ***KLHL13*** | kelch-like 13 (Drosophila) | |
| ***LDL*** | low density lipoprotein | |
| ***LDL-R*** | low density lipoprotein receptor | |
| ***LEF1*** | lymphoid enhancer-binding factor 1 | |
| ***LIF*** | leukemia inhibitory factor | |
| ***LMO2*** | LIM domain only 2 (rhombotin-like 1 | |
| ***LRRTM4*** | leucine rich repeat transmembrane neuronal 4 | |
| ***MALL*** | mal, T-cell differentiation protein-like | |
| ***MAPK*** | mitogen-activated protein kinase | |
| ***MARK1*** | MAP/microtubule affinity-regulating kinase 1 | |
| ***MFAP5*** | microfibrillar associated protein 5 | |
| ***MMP*** | matrix metalloproteinase | |
| ***MYCN*** | v-myc myelocytomatosis viral related oncogene, neuroblastoma derived | |
| ***NDP*** | Norrie disease (pseudoglioma) | |
| ***NPPB*** | natriuretic peptide B | |
| ***NPPC*** | natriuretic peptide precursor C | |
| ***NPR1*** | natriuretic peptide receptor A | |
| ***NPY*** | neuropeptide Y | |
| ***NRXN1*** | neurexin 1 | |
| ***PDCD11*** | programmed cell death 11 | |
| ***PHLDA1*** | pleckstrin homology-like domain, family A, member 1 | |
| ***PODXL*** | podocalyxin-like | |
| ***PPAP2A*** | phosphatidic acid phosphatase type 2A | |
| ***PTPRR*** | protein tyrosine phosphatase, receptor type, R | |
| ***RGS5*** | regulator of G-protein signaling 5 | |
| ***RIMS2*** | regulating synaptic membrane exocytosis 2 | |
| ***ROR1*** | receptor tyrosine kinase-like orphan receptor 1 | |
| ***RRAD*** | Ras-related associated with diabetes | |
| ***RSAD2*** | radical S-adenosyl methionine domain containing 2 | |
| ***RTN1*** | reticulon 1 | |
| ***SERPINB2*** | serpin peptidase inhibitor, clade B, member 2 | |
| ***SERPINB7*** | serpin peptidase inhibitor, clade B, member 7 | |
| ***SFRP2*** | secreted frizzled-related protein 2 | |
| ***SLC9A3R2*** | solute carrier family 9 (sodium/hydrogen exchanger), member 3 regulator 2 | |
| ***SORBS2*** | sorbin and SH3 domain containing 2 | |
| ***SOX13*** | SRY (sex determining region Y)-box 13 | |
| ***STC1*** | stanniocalcin 1 | |
| ***TES*** | testis derived transcript | |
| ***TGF-β1*** | Transforming Growth Factor Beta-1 | |
| ***TM6SF1*** | transmembrane 6 superfamily member 1 | |
| ***TPBG*** | trophoblast glycoprotein | |
| ***TPPP*** | tubulin polymerization promoting protein | |
| ***TPPP3*** | tubulin polymerization-promoting protein family member 3 | |
| ***UTY*** | ubiquitously transcribed tetratricopeptide repeat gene, Y-linked | |
| ***VEGF*** | vascular endothelial growth factor | |
| ***VEGFR-2*** | vascular endothelial growth factor receptor 2 | |
| ***VSNL1*** | visinin-like 1 | |
| ***ZNF512B*** | zinc finger protein 512B | |
