# Supplementary material for: Sex-Related Differences in Gene Expression by Porcine Aortic Valvular Interstitial Cells
Source: PLoS One. 2012 Jul 10;7(7):e39980. doi: 10.1371/journal.pone.0039980 (PMC3393722; doi:10.1371/journal.pone.0039980)
Supplement: Table S2 — Microarray genes found to be significantly different in male versus female samples. LogFC refers to the log2-based fold change between the sexes. t is the moderated t-statistic from the empirical Bayes methods. P value is the raw p value and the adj. P value is the p value adjusted for multiple comparisons using the Benjamini and Hochberg’s false discovery rate. B is log-odds that the gene is differentially expressed. (DOC) [file pone.0039980.s006.doc]

**Table S2**. Microarray genes found to be significantly different in male versus female samples. LogFC refers to the log2-based fold change between the sexes. t is the moderated t-statistic from the empirical Bayes methods. P value is the raw p value and the adj. P value is the p value adjusted for multiple comparisons using the Benjamini and Hochberg’s false discovery rate. B is log-odds that the gene is differentially expressed.

| **Probeset ID** | **Gene Symbol** | **Gene Name** | **UniGene SscID** | **logFC** | **t** | **P value** | **adj.**  **P value** | **B** |
| --- | --- | --- | --- | --- | --- | --- | --- | --- |
| Ssc.6667.1.A1_at | FAM5C | DBCCR1-like [Homo sapiens] | [73770](http://www.ncbi.nlm.nih.gov/UniGene/clust.cgi?ORG=Ssc&CID=73770) | -8.2 | -27 | 1.68E-11 | 4.10E-08 | 15.78 |
| Ssc.31029.1.A1_at | XIST | Receptor-type protein-tyrosine phosphatase mu precursor | [52384](http://www.ncbi.nlm.nih.gov/UniGene/clust.cgi?ORG=Ssc&CID=52384) | -7.3 | -23 | 7.96E-11 | 7.40E-08 | 14.65 |
| Ssc.7473.1.A1_at | DDX3Y | DEAD-box protein 3, Y-chromosomal | [9426](http://www.ncbi.nlm.nih.gov/UniGene/clust.cgi?ORG=Ssc&CID=9426) | 7.7 | 23 | 9.15E-11 | 7.40E-08 | 14.54 |
| Ssc.26799.1.S1_at | EIF1AY | Eukaryotic translation initiation factor 1A, Y-chromosomal | [26799](http://www.ncbi.nlm.nih.gov/UniGene/clust.cgi?ORG=Ssc&CID=26799) | 6.7 | 22 | 1.63E-10 | 9.80E-08 | 14.09 |
| Ssc.27304.1.S1_at | TMSB4X | thymosin-like 1 [Homo sapiens] | [54363](http://www.ncbi.nlm.nih.gov/UniGene/clust.cgi?ORG=Ssc&CID=54363) | 6.5 | 21 | 2.39E-10 | 1.20E-07 | 13.79 |
| Ssc.13426.1.A1_at | XIST | c-myc promoter binding protein; c-myc promoter-binding protein | [13426](http://www.ncbi.nlm.nih.gov/UniGene/clust.cgi?ORG=Ssc&CID=13426) | -6.2 | -20 | 3.90E-10 | 1.50E-07 | 13.40 |
| Ssc.16426.1.S1_at | EIF2S3 | Eukaryotic translation initiation factor 2 subunit 3 | [16426](http://www.ncbi.nlm.nih.gov/UniGene/clust.cgi?ORG=Ssc&CID=16426) | 6.5 | 20 | 4.27E-10 | 1.50E-07 | 13.32 |
| Ssc.4897.1.A1_at | XIST | Circadian locomoter output cycles kaput protein (hCLOCK) | [73691](http://www.ncbi.nlm.nih.gov/UniGene/clust.cgi?ORG=Ssc&CID=73691) | -6.5 | -20 | 5.56E-10 | 1.70E-07 | 13.10 |
| Ssc.21512.1.A1_at | LPHN2 | Latrophilin 2 precursor | [21512](http://www.ncbi.nlm.nih.gov/UniGene/clust.cgi?ORG=Ssc&CID=21512) | -5.9 | -16 | 3.48E-09 | 9.30E-07 | 11.52 |
| Ssc.16169.1.S1_x_at | HLA-DRB4 | HLA class II histocompatibility antigen, DRB1-4 beta chain protein | [---](http://www.ncbi.nlm.nih.gov/UniGene/clust.cgi?ORG=Ssc&CID=---) | 5.9 | 15 | 9.81E-09 | 2.40E-06 | 10.59 |
| Ssc.21814.1.S1_at | JARID1C | Jumonji/ARID domain-containing protein 1C (SmcX protein) | [21814](http://www.ncbi.nlm.nih.gov/UniGene/clust.cgi?ORG=Ssc&CID=21814) | 4.4 | 13 | 5.34E-08 | 1.20E-05 | 9.00 |
| Ssc.6186.1.A1_at | VSNL1 | visinin-like 1 [Homo sapiens] | [56137](http://www.ncbi.nlm.nih.gov/UniGene/clust.cgi?ORG=Ssc&CID=56137) | 4.3 | 9.3 | 1.38E-06 | 2.80E-04 | 5.81 |
| Ssc.2434.1.A1_at | XIST | Homeodomain-interacting protein kinase 2 | [4897](http://www.ncbi.nlm.nih.gov/UniGene/clust.cgi?ORG=Ssc&CID=4897) | -3.9 | -9.1 | 1.76E-06 | 3.30E-04 | 5.56 |
| Ssc.15981.1.S1_at | NPY | Neuropeptide Y precursor | [15981](http://www.ncbi.nlm.nih.gov/UniGene/clust.cgi?ORG=Ssc&CID=15981) | 4.4 | 8.3 | 4.03E-06 | 7.00E-04 | 4.73 |
| Ssc.22487.1.S1_at | C2orf40 | esophageal cancer related gene 4 protein [Homo sapiens] | [---](http://www.ncbi.nlm.nih.gov/UniGene/clust.cgi?ORG=Ssc&CID=---) | 2.7 | 8.1 | 4.98E-06 | 8.00E-04 | 4.51 |
| Ssc.18418.1.A1_at | Q9H4Z9 | DJ846F13.1 (Phosphatidic acid phosphatase type 2c) | [---](http://www.ncbi.nlm.nih.gov/UniGene/clust.cgi?ORG=Ssc&CID=---) | 3 | 8.1 | 5.41E-06 | 8.00E-04 | 4.43 |
| Ssc.27236.1.S1_at | UTY | Ubiquitously transcribed Y chromosome tetratricopeptide repeat | [27236](http://www.ncbi.nlm.nih.gov/UniGene/clust.cgi?ORG=Ssc&CID=27236) | 3 | 8 | 5.65E-06 | 8.00E-04 | 4.39 |
| Ssc.17325.1.S1_at | CD24L4 | Signal transducer CD24 precursor | [17325](http://www.ncbi.nlm.nih.gov/UniGene/clust.cgi?ORG=Ssc&CID=17325) | 3.5 | 7.9 | 6.88E-06 | 9.20E-04 | 4.19 |
| Ssc.13293.1.A1_at | GABRB2 |  | [13293](http://www.ncbi.nlm.nih.gov/UniGene/clust.cgi?ORG=Ssc&CID=13293) | 2.7 | 7.2 | 1.61E-05 | 2.00E-03 | 3.32 |
| Ssc.8291.1.A1_at | TMEM16C | Transmembrane protein 16C. | [8291](http://www.ncbi.nlm.nih.gov/UniGene/clust.cgi?ORG=Ssc&CID=8291) | 2.6 | 7 | 2.12E-05 | 2.60E-03 | 3.04 |
| Ssc.17428.1.S1_at | GABRA1 | Gamma-aminobutyric-acid receptor alpha-1 subunit precursor | [17428](http://www.ncbi.nlm.nih.gov/UniGene/clust.cgi?ORG=Ssc&CID=17428) | 2.4 | 6.9 | 2.40E-05 | 2.80E-03 | 2.91 |
| Ssc.13820.1.A1_at | PTPRK | Receptor-type protein-tyrosine phosphatase kappa precursor | [13820](http://www.ncbi.nlm.nih.gov/UniGene/clust.cgi?ORG=Ssc&CID=13820) | -2.4 | -6.8 | 2.58E-05 | 2.80E-03 | 2.84 |
| Ssc.19564.1.S1_at | PCDH9 | Protocadherin 9 precursor | [19564](http://www.ncbi.nlm.nih.gov/UniGene/clust.cgi?ORG=Ssc&CID=19564) | -2.4 | -6.8 | 2.83E-05 | 3.00E-03 | 2.74 |
| Ssc.24783.1.A1_at | IL17D | Interleukin-17D precursor | [24783](http://www.ncbi.nlm.nih.gov/UniGene/clust.cgi?ORG=Ssc&CID=24783) | 2.2 | 6.4 | 4.61E-05 | 4.60E-03 | 2.24 |
| Ssc.10174.1.A1_at | DNAH14 | Novel protein (Fragment) | [10174](http://www.ncbi.nlm.nih.gov/UniGene/clust.cgi?ORG=Ssc&CID=10174) | 2.2 | 6.3 | 5.57E-05 | 5.30E-03 | 2.05 |
| Ssc.2464.1.S1_at | STC1 | Stanniocalcin 1 precursor (STC-1) | [15105](http://www.ncbi.nlm.nih.gov/UniGene/clust.cgi?ORG=Ssc&CID=15105) | 2.4 | 6.3 | 5.71E-05 | 5.30E-03 | 2.02 |
| Ssc.22413.1.A1_at | TPBG | 5T4 oncofetal trophoblast glycoprotein; 5T4-antigen | [22413](http://www.ncbi.nlm.nih.gov/UniGene/clust.cgi?ORG=Ssc&CID=22413) | 2.2 | 6.2 | 6.36E-05 | 5.70E-03 | 1.91 |
| Ssc.21194.1.S1_at | PTPRR | Receptor-type protein-tyrosine phosphatase R precursor | [55667](http://www.ncbi.nlm.nih.gov/UniGene/clust.cgi?ORG=Ssc&CID=55667) | 2.2 | 6.1 | 6.83E-05 | 5.90E-03 | 1.84 |
| Ssc.13650.1.A1_at | RIMS2 | Regulating synaptic membrane exocytosis protein 2 | [48396](http://www.ncbi.nlm.nih.gov/UniGene/clust.cgi?ORG=Ssc&CID=48396) | 1.9 | 6.1 | 7.45E-05 | 6.20E-03 | 1.75 |
| Ssc.30334.1.A1_at | CYP3A4 | Cytochrome P450 3A4 | [70525](http://www.ncbi.nlm.nih.gov/UniGene/clust.cgi?ORG=Ssc&CID=70525) | -2.9 | -6 | 8.03E-05 | 6.50E-03 | 1.67 |
| Ssc.25045.1.S1_at | KDR | Vascular endothelial growth factor receptor 2 precursor | [70737](http://www.ncbi.nlm.nih.gov/UniGene/clust.cgi?ORG=Ssc&CID=70737) | 2.4 | 6 | 8.80E-05 | 6.80E-03 | 1.58 |
| Ssc.7847.1.S1_at | KCNIP4 | potassium channel-interacting protein 4 isoform 2; calsenili | [7847](http://www.ncbi.nlm.nih.gov/UniGene/clust.cgi?ORG=Ssc&CID=7847) | -1.8 | -5.9 | 9.58E-05 | 7.20E-03 | 1.49 |
| Ssc.11281.1.A1_at | PENK | Proenkephalin A precursor | [---](http://www.ncbi.nlm.nih.gov/UniGene/clust.cgi?ORG=Ssc&CID=---) | -2.7 | -5.8 | 1.05E-04 | 7.70E-03 | 1.40 |
| Ssc.27410.1.S1_at | MYCN | N-myc proto-oncogene protein | [55549](http://www.ncbi.nlm.nih.gov/UniGene/clust.cgi?ORG=Ssc&CID=55549) | 2.4 | 5.8 | 1.17E-04 | 8.30E-03 | 1.29 |
| Ssc.21139.2.S1_at | CLIC5 | Chloride intracellular channel protein 5 | [42906](http://www.ncbi.nlm.nih.gov/UniGene/clust.cgi?ORG=Ssc&CID=42906) | 2.1 | 5.6 | 1.49E-04 | 9.90E-03 | 1.04 |
| Ssc.18011.1.S1_at | POPDC3 | Popeye domain containing protein 3 (Popeye protein 3) | [18011](http://www.ncbi.nlm.nih.gov/UniGene/clust.cgi?ORG=Ssc&CID=18011) | 1.9 | 5.6 | 1.51E-04 | 9.90E-03 | 1.02 |
| Ssc.15327.1.S1_at | CR2 | Complement receptor type 2 precursor (Cr2) | [70456](http://www.ncbi.nlm.nih.gov/UniGene/clust.cgi?ORG=Ssc&CID=70456) | -2.7 | -5.6 | 1.60E-04 | 9.90E-03 | 0.97 |
| Ssc.6357.1.S1_at | KCNK1 | Potassium channel subfamily K member 1 | [6357](http://www.ncbi.nlm.nih.gov/UniGene/clust.cgi?ORG=Ssc&CID=6357) | -2.3 | -5.6 | 1.62E-04 | 9.90E-03 | 0.95 |
| Ssc.10582.1.A1_at | STOX1 |  | [10582](http://www.ncbi.nlm.nih.gov/UniGene/clust.cgi?ORG=Ssc&CID=10582) | 1.8 | 5.5 | 1.67E-04 | 9.90E-03 | 0.92 |
| Ssc.29330.1.S1_at | LRRTM4 | leucine rich repeat transmembrane neuronal 4 | [40821](http://www.ncbi.nlm.nih.gov/UniGene/clust.cgi?ORG=Ssc&CID=40821) | 1.7 | 5.5 | 1.70E-04 | 9.90E-03 | 0.90 |
| Ssc.4945.1.S1_at | SLC9A3R2 | Na(+)/H(+) exchange regulatory cofactor NHE-RF2 (NHERF-2) | [4945](http://www.ncbi.nlm.nih.gov/UniGene/clust.cgi?ORG=Ssc&CID=4945) | 2.1 | 5.5 | 1.74E-04 | 9.90E-03 | 0.88 |
| Ssc.15907.1.S1_at | ACAN | Aggrecan core protein precursor | [15907](http://www.ncbi.nlm.nih.gov/UniGene/clust.cgi?ORG=Ssc&CID=15907) | -2.3 | -5.5 | 1.76E-04 | 9.90E-03 | 0.87 |
| Ssc.2001.1.A1_at | LHX6 | LIM/homeobox protein Lhx6.1 (Lhx6) | [2001](http://www.ncbi.nlm.nih.gov/UniGene/clust.cgi?ORG=Ssc&CID=2001) | 1.8 | 5.5 | 1.80E-04 | 9.90E-03 | 0.84 |
| Ssc.3850.1.S1_at | NNAT | Neuronatin | [3850](http://www.ncbi.nlm.nih.gov/UniGene/clust.cgi?ORG=Ssc&CID=3850) | -2 | -5.5 | 1.80E-04 | 9.90E-03 | 0.84 |
| Ssc.1342.1.S1_at | APOE | Apolipoprotein E precursor (Apo-E) | [1342](http://www.ncbi.nlm.nih.gov/UniGene/clust.cgi?ORG=Ssc&CID=1342) | 2 | 5.4 | 2.18E-04 | 1.10E-02 | 0.65 |
| Ssc.272.1.S1_a_at | CD55 | Complement decay-accelerating factor precursor | [272](http://www.ncbi.nlm.nih.gov/UniGene/clust.cgi?ORG=Ssc&CID=272) | 1.9 | 5.4 | 2.18E-04 | 1.10E-02 | 0.65 |
| Ssc.23390.1.A1_at | GNS | N-acetylglucosamine-6-sulfatase precursor | [23390](http://www.ncbi.nlm.nih.gov/UniGene/clust.cgi?ORG=Ssc&CID=23390) | 1.9 | 5.4 | 2.21E-04 | 1.10E-02 | 0.63 |
| Ssc.6788.1.A1_at | SORBS2 | Arg/Abl-interacting protein 2 isoform 1; Arg binding protein | [6788](http://www.ncbi.nlm.nih.gov/UniGene/clust.cgi?ORG=Ssc&CID=6788) | 2.2 | 5.3 | 2.32E-04 | 1.20E-02 | 0.58 |
| Ssc.9434.1.A1_at | MARK1 | MAP/microtubule affinity-regulating kinase 1 [Homo sapiens] | [43683](http://www.ncbi.nlm.nih.gov/UniGene/clust.cgi?ORG=Ssc&CID=43683) | 2.2 | 5.3 | 2.36E-04 | 1.20E-02 | 0.57 |
| Ssc.19540.1.S1_at | TPPP3 | Protein CGI-38 | [19540](http://www.ncbi.nlm.nih.gov/UniGene/clust.cgi?ORG=Ssc&CID=19540) | 2 | 5.3 | 2.55E-04 | 1.20E-02 | 0.49 |
| Ssc.16236.1.S1_at | DPP4 | Dipeptidyl peptidase IV | [16236](http://www.ncbi.nlm.nih.gov/UniGene/clust.cgi?ORG=Ssc&CID=16236) | 3.2 | 5.2 | 2.67E-04 | 1.30E-02 | 0.44 |
| Ssc.4958.1.A1_at | Q6ZV50 |  | [4958](http://www.ncbi.nlm.nih.gov/UniGene/clust.cgi?ORG=Ssc&CID=4958) | 1.8 | 5.2 | 2.82E-04 | 1.30E-02 | 0.38 |
| Ssc.21139.1.S1_at | CLIC5 | Chloride intracellular channel protein 5 | [42906](http://www.ncbi.nlm.nih.gov/UniGene/clust.cgi?ORG=Ssc&CID=42906) | 1.9 | 5.2 | 2.94E-04 | 1.30E-02 | 0.34 |
| Ssc.1609.1.A1_at | Q8N675 |  | [73618](http://www.ncbi.nlm.nih.gov/UniGene/clust.cgi?ORG=Ssc&CID=73618) | 1.9 | 5.1 | 3.04E-04 | 1.40E-02 | 0.31 |
| Ssc.13640.1.A1_at | STOX1 |  | [13640](http://www.ncbi.nlm.nih.gov/UniGene/clust.cgi?ORG=Ssc&CID=13640) | 1.9 | 5.1 | 3.38E-04 | 1.50E-02 | 0.20 |
| Ssc.31164.1.S1_at | MYO1B | Myosin Ib (Myosin I alpha) (MMI-alpha) (MMIa) (MYH-1c) | [48562](http://www.ncbi.nlm.nih.gov/UniGene/clust.cgi?ORG=Ssc&CID=48562) | -2 | -5.1 | 3.52E-04 | 1.50E-02 | 0.15 |
| Ssc.26250.1.S1_at | PDCD11 | RRP5 protein homolog (Programmed cell death protein 11) | [26250](http://www.ncbi.nlm.nih.gov/UniGene/clust.cgi?ORG=Ssc&CID=26250) | 2.3 | 5 | 3.58E-04 | 1.50E-02 | 0.14 |
| Ssc.26633.1.A1_at | SLC16A10 | solute carrier family 16, member 10; T-type amino acid trans | [26633](http://www.ncbi.nlm.nih.gov/UniGene/clust.cgi?ORG=Ssc&CID=26633) | -1.6 | -5 | 3.77E-04 | 1.60E-02 | 0.08 |
| Ssc.5051.1.S1_at | ETF1 | eukaryotic translation termination factor 1; polypeptide cha | [5051](http://www.ncbi.nlm.nih.gov/UniGene/clust.cgi?ORG=Ssc&CID=5051) | 1.9 | 5 | 4.06E-04 | 1.70E-02 | 0.01 |
| Ssc.6258.1.A1_a_at | ITGA6 | Integrin alpha-6 precursor (VLA-6) (CD49f) | [6258](http://www.ncbi.nlm.nih.gov/UniGene/clust.cgi?ORG=Ssc&CID=6258) | 1.8 | 4.9 | 4.77E-04 | 1.90E-02 | -0.16 |
| Ssc.23468.2.S1_at | PPAP2A | Lipid phosphate phosphohydrolase 1 | [26067](http://www.ncbi.nlm.nih.gov/UniGene/clust.cgi?ORG=Ssc&CID=26067) | 2 | 4.8 | 5.29E-04 | 2.10E-02 | -0.26 |
| Ssc.13346.1.A1_at | FRMD5 |  | [42486](http://www.ncbi.nlm.nih.gov/UniGene/clust.cgi?ORG=Ssc&CID=42486) | -1.8 | -4.8 | 5.32E-04 | 2.10E-02 | -0.27 |
| Ssc.9684.1.S1_at | SLC16A10 | solute carrier family 16, member 10; T-type amino acid trans | [44813](http://www.ncbi.nlm.nih.gov/UniGene/clust.cgi?ORG=Ssc&CID=44813) | -1.7 | -4.8 | 5.56E-04 | 2.10E-02 | -0.32 |
| Ssc.6510.1.A1_at | FBLN7 |  | [---](http://www.ncbi.nlm.nih.gov/UniGene/clust.cgi?ORG=Ssc&CID=---) | -1.5 | -4.8 | 5.73E-04 | 2.20E-02 | -0.35 |
| Ssc.19640.1.A1_at | FCER1A | High affinity immunoglobulin epsilon receptor alpha-subunit | [19640](http://www.ncbi.nlm.nih.gov/UniGene/clust.cgi?ORG=Ssc&CID=19640) | 2 | 4.7 | 6.37E-04 | 2.40E-02 | -0.45 |
| Ssc.25348.1.S1_at | TEX12 | Testis expressed protein 12 | [25348](http://www.ncbi.nlm.nih.gov/UniGene/clust.cgi?ORG=Ssc&CID=25348) | -1.4 | -4.6 | 7.02E-04 | 2.50E-02 | -0.55 |
| Ssc.13458.1.A1_at | RASSF8 | Protein C12orf2 (Carcinoma associated protein HOJ-1) | [13458](http://www.ncbi.nlm.nih.gov/UniGene/clust.cgi?ORG=Ssc&CID=13458) | -1.5 | -4.6 | 7.08E-04 | 2.50E-02 | -0.56 |
| Ssc.14467.2.S1_a_at | AREG | Amphiregulin precursor (AR) | [14467](http://www.ncbi.nlm.nih.gov/UniGene/clust.cgi?ORG=Ssc&CID=14467) | 2.3 | 4.6 | 7.28E-04 | 2.60E-02 | -0.59 |
| Ssc.19722.1.S1_at | ELTD1 | EGF, latrophilin and seven transmembrane domain containing p | [19722](http://www.ncbi.nlm.nih.gov/UniGene/clust.cgi?ORG=Ssc&CID=19722) | 1.8 | 4.6 | 7.39E-04 | 2.60E-02 | -0.61 |
| Ssc.12145.1.A1_at | MALL | BENE protein | [12145](http://www.ncbi.nlm.nih.gov/UniGene/clust.cgi?ORG=Ssc&CID=12145) | 2.3 | 4.6 | 7.55E-04 | 2.60E-02 | -0.63 |
| Ssc.26337.1.S1_at | ICAM2 | Intercellular adhesion molecule-2 precursor | [26337](http://www.ncbi.nlm.nih.gov/UniGene/clust.cgi?ORG=Ssc&CID=26337) | 1.7 | 4.6 | 7.64E-04 | 2.60E-02 | -0.64 |
| Ssc.7946.1.S1_at | PCDH9 | Protocadherin 9 precursor | [7946](http://www.ncbi.nlm.nih.gov/UniGene/clust.cgi?ORG=Ssc&CID=7946) | -1.5 | -4.5 | 8.40E-04 | 2.80E-02 | -0.74 |
| Ssc.24926.1.S1_at | NPR1 | Atrial natriuretic peptide receptor A precursor | [24926](http://www.ncbi.nlm.nih.gov/UniGene/clust.cgi?ORG=Ssc&CID=24926) | 1.4 | 4.5 | 8.40E-04 | 2.80E-02 | -0.74 |
| Ssc.5799.1.S1_at | WIF1 | Wnt inhibitory factor 1 precursor (WIF-1) | [5799](http://www.ncbi.nlm.nih.gov/UniGene/clust.cgi?ORG=Ssc&CID=5799) | -2 | -4.5 | 8.49E-04 | 2.80E-02 | -0.75 |
| Ssc.11342.1.A1_at | CNIH4 | Protein HSPC163 | [11342](http://www.ncbi.nlm.nih.gov/UniGene/clust.cgi?ORG=Ssc&CID=11342) | -1.5 | -4.5 | 8.76E-04 | 2.80E-02 | -0.78 |
| Ssc.6060.1.S1_at | ARHGEF15 | Rho guanine nucleotide exchange factor 15 (Vsm-RhoGEF) | [6060](http://www.ncbi.nlm.nih.gov/UniGene/clust.cgi?ORG=Ssc&CID=6060) | 1.8 | 4.5 | 8.82E-04 | 2.80E-02 | -0.79 |
| Ssc.6930.2.A1_at | FRY |  | [28167](http://www.ncbi.nlm.nih.gov/UniGene/clust.cgi?ORG=Ssc&CID=28167) | 1.9 | 4.5 | 9.04E-04 | 2.80E-02 | -0.81 |
| Ssc.27794.2.S1_at | BRPF1 | ARP2/3 complex 20 kDa subunit (p20-ARC) (Actin-related prote | [10726](http://www.ncbi.nlm.nih.gov/UniGene/clust.cgi?ORG=Ssc&CID=10726) | -1.7 | -4.5 | 9.24E-04 | 2.80E-02 | -0.84 |
| Ssc.15105.1.S1_at | STC1 | Stanniocalcin 1 precursor (STC-1) | [15105](http://www.ncbi.nlm.nih.gov/UniGene/clust.cgi?ORG=Ssc&CID=15105) | 1.4 | 4.4 | 9.52E-04 | 2.80E-02 | -0.87 |
| Ssc.25207.1.A1_at | ITGA6 | Integrin alpha-6 precursor (VLA-6) (CD49f) | [25207](http://www.ncbi.nlm.nih.gov/UniGene/clust.cgi?ORG=Ssc&CID=25207) | 1.6 | 4.4 | 9.53E-04 | 2.80E-02 | -0.87 |
| Ssc.29681.1.A1_at | ROR1 | Tyrosine-protein kinase transmembrane receptor ROR1 precurso | [40517](http://www.ncbi.nlm.nih.gov/UniGene/clust.cgi?ORG=Ssc&CID=40517) | 1.4 | 4.4 | 9.53E-04 | 2.80E-02 | -0.87 |
| Ssc.15973.1.S1_at | ENTPD1 | Ectonucleoside triphosphate diphosphohydrolase 1 | [52334](http://www.ncbi.nlm.nih.gov/UniGene/clust.cgi?ORG=Ssc&CID=52334) | 1.9 | 4.4 | 9.73E-04 | 2.90E-02 | -0.89 |
| Ssc.3326.1.S1_at | CITED1 | Cbp/p300-interacting transactivator 1 | [3326](http://www.ncbi.nlm.nih.gov/UniGene/clust.cgi?ORG=Ssc&CID=3326) | -1.7 | -4.4 | 9.94E-04 | 2.90E-02 | -0.91 |
| Ssc.6978.1.A1_at | MAX | Max protein | [6978](http://www.ncbi.nlm.nih.gov/UniGene/clust.cgi?ORG=Ssc&CID=6978) | -1.4 | -4.4 | 1.01E-03 | 2.90E-02 | -0.93 |
| Ssc.28701.1.S1_at | SORBS2 | Arg/Abl-interacting protein 2 isoform 1; Arg binding protein | [55611](http://www.ncbi.nlm.nih.gov/UniGene/clust.cgi?ORG=Ssc&CID=55611) | 2 | 4.4 | 1.05E-03 | 2.90E-02 | -0.96 |
| Ssc.12939.1.S1_at | CDH13 | Cadherin-13 precursor (Truncated-cadherin) (T-cadherin) | [12939](http://www.ncbi.nlm.nih.gov/UniGene/clust.cgi?ORG=Ssc&CID=12939) | 2 | 4.4 | 1.05E-03 | 2.90E-02 | -0.97 |
| Ssc.6305.1.S1_at | KHDRBS3 | KH domain containing, RNA binding, signal transduction assoc | [6305](http://www.ncbi.nlm.nih.gov/UniGene/clust.cgi?ORG=Ssc&CID=6305) | 1.7 | 4.4 | 1.06E-03 | 2.90E-02 | -0.98 |
| Ssc.19452.1.S1_at | TMEM45B |  | [19452](http://www.ncbi.nlm.nih.gov/UniGene/clust.cgi?ORG=Ssc&CID=19452) | 1.4 | 4.4 | 1.08E-03 | 3.00E-02 | -0.99 |
| Ssc.15378.1.A1_at | MCTP1 | multiple C2-domains with two transmembrane regions 1 isoform | [15378](http://www.ncbi.nlm.nih.gov/UniGene/clust.cgi?ORG=Ssc&CID=15378) | 1.5 | 4.4 | 1.09E-03 | 3.00E-02 | -1.00 |
| Ssc.4632.2.A1_at | ARHGEF3 | Rho guanine nucleotide exchange factor 3; exchange factor fo | [4632](http://www.ncbi.nlm.nih.gov/UniGene/clust.cgi?ORG=Ssc&CID=4632) | 1.4 | 4.3 | 1.13E-03 | 3.00E-02 | -1.04 |
| Ssc.15285.1.S1_at | TPPP | 25 kDa brain-specific protein (p25-alpha) | [21481](http://www.ncbi.nlm.nih.gov/UniGene/clust.cgi?ORG=Ssc&CID=21481) | 1.5 | 4.3 | 1.15E-03 | 3.00E-02 | -1.06 |
| Ssc.20051.1.S1_at | NDP | Norrin (Norrie disease protein) | [20051](http://www.ncbi.nlm.nih.gov/UniGene/clust.cgi?ORG=Ssc&CID=20051) | 1.4 | 4.3 | 1.15E-03 | 3.00E-02 | -1.06 |
| Ssc.29380.1.A1_at | PRDM6 | PR-domain zinc finger protein 6 (Fragment) | [---](http://www.ncbi.nlm.nih.gov/UniGene/clust.cgi?ORG=Ssc&CID=---) | -1.4 | -4.3 | 1.18E-03 | 3.10E-02 | -1.09 |
| Ssc.18466.2.S1_at | CDH11 | Cadherin-11 precursor (Osteoblast-cadherin) (OB-cadherin) | [18466](http://www.ncbi.nlm.nih.gov/UniGene/clust.cgi?ORG=Ssc&CID=18466) | 1.5 | 4.3 | 1.21E-03 | 3.10E-02 | -1.11 |
| Ssc.7552.1.A1_at | TES | Testin (TESS) | [46797](http://www.ncbi.nlm.nih.gov/UniGene/clust.cgi?ORG=Ssc&CID=46797) | 1.9 | 4.2 | 1.34E-03 | 3.30E-02 | -1.21 |
| Ssc.31166.1.S1_at | ABCA6 | ATP-binding cassette, sub-family A, member 6 isoform a; ATP- | [59202](http://www.ncbi.nlm.nih.gov/UniGene/clust.cgi?ORG=Ssc&CID=59202) | -1.4 | -4.2 | 1.34E-03 | 3.30E-02 | -1.21 |
| Ssc.12452.1.S1_at | MYO1B | Myosin Ib (Myosin I alpha) (MMI-alpha) (MMIa) (MYH-1c) | [12452](http://www.ncbi.nlm.nih.gov/UniGene/clust.cgi?ORG=Ssc&CID=12452) | -1.6 | -4.2 | 1.38E-03 | 3.30E-02 | -1.25 |
| Ssc.12966.1.S1_at | LMO2 | Rhombotin-2 (Cysteine-rich protein TTG-2) (T-cell translocat | [---](http://www.ncbi.nlm.nih.gov/UniGene/clust.cgi?ORG=Ssc&CID=---) | 1.5 | 4.2 | 1.40E-03 | 3.30E-02 | -1.26 |
| Ssc.4681.1.S1_at | CD93 | Complement component C1q receptor precursor (Complement comp | [74967](http://www.ncbi.nlm.nih.gov/UniGene/clust.cgi?ORG=Ssc&CID=74967) | 1.5 | 4.2 | 1.41E-03 | 3.30E-02 | -1.27 |
| Ssc.18554.1.S1_x_at | HLA-B | HLA class I histocompatibility antigen, B-7 alpha chain prec | [18554](http://www.ncbi.nlm.nih.gov/UniGene/clust.cgi?ORG=Ssc&CID=18554) | 1.6 | 4.2 | 1.41E-03 | 3.30E-02 | -1.27 |
| Ssc.29677.1.A1_at | ABI3 | ABI gene family member 3 (New molecule including SH3) (Nesh) | [---](http://www.ncbi.nlm.nih.gov/UniGene/clust.cgi?ORG=Ssc&CID=---) | 1.7 | 4.2 | 1.41E-03 | 3.30E-02 | -1.27 |
| Ssc.23867.1.A1_at | NPPC | C-type natriuretic peptide precursor [Contains: CNP-22; CNP- | [23867](http://www.ncbi.nlm.nih.gov/UniGene/clust.cgi?ORG=Ssc&CID=23867) | 1.5 | 4.2 | 1.42E-03 | 3.30E-02 | -1.28 |
| Ssc.5204.1.S1_at | CDA | Cytidine deaminase (EC 3.5.4.5) (Cytidine aminohydrolase). [ | [---](http://www.ncbi.nlm.nih.gov/UniGene/clust.cgi?ORG=Ssc&CID=---) | 1.7 | 4.2 | 1.43E-03 | 3.30E-02 | -1.28 |
| Ssc.2104.1.S1_at | Q8TAL6 | similar to RIKEN cDNA 1110018M03 | [---](http://www.ncbi.nlm.nih.gov/UniGene/clust.cgi?ORG=Ssc&CID=---) | -2 | -4.2 | 1.44E-03 | 3.30E-02 | -1.29 |
| Ssc.15783.1.S1_at | CALCRL | Calcitonin gene-related peptide type 1 receptor precursor (C | [2695](http://www.ncbi.nlm.nih.gov/UniGene/clust.cgi?ORG=Ssc&CID=2695) | 1.4 | 4.2 | 1.48E-03 | 3.40E-02 | -1.32 |
| Ssc.286.1.S1_s_at | RSAD2 | viperin; similar to inflammatory response protein 6 [Homo sa | [286](http://www.ncbi.nlm.nih.gov/UniGene/clust.cgi?ORG=Ssc&CID=286) | 1.8 | 4.2 | 1.49E-03 | 3.40E-02 | -1.32 |
| Ssc.26552.1.A1_at | ADAMTS17 |  | [26552](http://www.ncbi.nlm.nih.gov/UniGene/clust.cgi?ORG=Ssc&CID=26552) | -1.3 | -4.1 | 1.64E-03 | 3.70E-02 | -1.42 |
| Ssc.4217.1.S1_at | ITIH4 | Inter-alpha-trypsin inhibitor heavy chain H4 precursor (ITI | [4217](http://www.ncbi.nlm.nih.gov/UniGene/clust.cgi?ORG=Ssc&CID=4217) | 1.8 | 4.1 | 1.65E-03 | 3.70E-02 | -1.43 |
| Ssc.26067.1.S1_at | PODXL | Podocalyxin-like protein 1 precursor. | [26067](http://www.ncbi.nlm.nih.gov/UniGene/clust.cgi?ORG=Ssc&CID=26067) | 1.8 | 4.1 | 1.68E-03 | 3.70E-02 | -1.45 |
| Ssc.11310.1.A1_at | ANK3 | Ankyrin 3 (ANK-3) (Ankyrin G) | [57668](http://www.ncbi.nlm.nih.gov/UniGene/clust.cgi?ORG=Ssc&CID=57668) | -1.6 | -4.1 | 1.69E-03 | 3.70E-02 | -1.45 |
| Ssc.16145.1.S1_at | HTR2B | 5-hydroxytryptamine 2B receptor (5-HT-2B) (Serotonin recepto | [16145](http://www.ncbi.nlm.nih.gov/UniGene/clust.cgi?ORG=Ssc&CID=16145) | 1.5 | 4.1 | 1.76E-03 | 3.80E-02 | -1.49 |
| Ssc.11729.1.A1_at | SNF1LK2 | salt-inducible kinase 2 [Homo sapiens] | [11729](http://www.ncbi.nlm.nih.gov/UniGene/clust.cgi?ORG=Ssc&CID=11729) | -1.3 | -4.1 | 1.77E-03 | 3.80E-02 | -1.50 |
| Ssc.26326.1.S1_at | CYP3A4 | Cytochrome P450 3A4 (EC 1.14.13.67) (Quinine 3-monooxygenase | [70525](http://www.ncbi.nlm.nih.gov/UniGene/clust.cgi?ORG=Ssc&CID=70525) | -1.7 | -4 | 1.88E-03 | 3.90E-02 | -1.56 |
| Ssc.3107.2.A1_at | CLEC14A | Protein C14orf27 precursor | [---](http://www.ncbi.nlm.nih.gov/UniGene/clust.cgi?ORG=Ssc&CID=---) | 1.5 | 4 | 1.89E-03 | 3.90E-02 | -1.56 |
| Ssc.24173.1.S1_at | DDX4 | DEAD-box protein 4 (VASA homolog) | [24173](http://www.ncbi.nlm.nih.gov/UniGene/clust.cgi?ORG=Ssc&CID=24173) | -1.6 | -4 | 1.90E-03 | 3.90E-02 | -1.57 |
| Ssc.1557.1.S1_at | ESAM | endothelial cell adhesion molecule; 2310008D05Rik; HUEL (C4o | [---](http://www.ncbi.nlm.nih.gov/UniGene/clust.cgi?ORG=Ssc&CID=---) | 1.6 | 4 | 1.91E-03 | 3.90E-02 | -1.57 |
| Ssc.4632.1.A1_at | ARHGEF3 | Rho guanine nucleotide exchange factor 3; exchange factor fo | [4632](http://www.ncbi.nlm.nih.gov/UniGene/clust.cgi?ORG=Ssc&CID=4632) | 1.3 | 4 | 1.92E-03 | 3.90E-02 | -1.58 |
| Ssc.15800.1.S1_at | IGFBP5 | Insulin-like growth factor binding protein 5 precursor (IGFB | [15800](http://www.ncbi.nlm.nih.gov/UniGene/clust.cgi?ORG=Ssc&CID=15800) | 1.6 | 4 | 1.93E-03 | 3.90E-02 | -1.59 |
| Ssc.14279.1.A1_at | EZH2 | Enhancer of zeste homolog 2 (ENX-1) | [14279](http://www.ncbi.nlm.nih.gov/UniGene/clust.cgi?ORG=Ssc&CID=14279) | -2.1 | -4 | 2.02E-03 | 4.10E-02 | -1.63 |
| Ssc.3693.1.S1_at | SERPINB7 | Megsin (TP55) (Serpin B7) | [45517](http://www.ncbi.nlm.nih.gov/UniGene/clust.cgi?ORG=Ssc&CID=45517) | 2.3 | 4 | 2.15E-03 | 4.20E-02 | -1.69 |
| Ssc.19959.1.S1_at | KIAA1324L |  | [19959](http://www.ncbi.nlm.nih.gov/UniGene/clust.cgi?ORG=Ssc&CID=19959) | -1.4 | -4 | 2.16E-03 | 4.20E-02 | -1.70 |
| Ssc.14043.1.A1_at | TMCC2 |  | [14043](http://www.ncbi.nlm.nih.gov/UniGene/clust.cgi?ORG=Ssc&CID=14043) | -1.3 | -4 | 2.16E-03 | 4.20E-02 | -1.70 |
| Ssc.21207.1.S1_at | GRPEL2 | GrpE protein homolog 2, mitochondrial precursor (Mt-GrpE#2). | [---](http://www.ncbi.nlm.nih.gov/UniGene/clust.cgi?ORG=Ssc&CID=---) | -1.9 | -4 | 2.16E-03 | 4.20E-02 | -1.70 |
| Ssc.19608.1.S1_at | RXRG | Retinoic acid receptor RXR-gamma | [19608](http://www.ncbi.nlm.nih.gov/UniGene/clust.cgi?ORG=Ssc&CID=19608) | 1.3 | 4 | 2.16E-03 | 4.20E-02 | -1.70 |
| Ssc.13916.1.S1_a_at | RTN1 | Reticulon 1 (Neuroendocrine-specific protein) | [13916](http://www.ncbi.nlm.nih.gov/UniGene/clust.cgi?ORG=Ssc&CID=13916) | 1.6 | 3.9 | 2.22E-03 | 4.30E-02 | -1.73 |
| Ssc.14025.1.A1_at | LEF1 | Lymphoid enhancer binding factor 1 (LEF-1) (T cell-specific | [14025](http://www.ncbi.nlm.nih.gov/UniGene/clust.cgi?ORG=Ssc&CID=14025) | 1.5 | 3.9 | 2.26E-03 | 4.30E-02 | -1.75 |
| Ssc.17635.2.S1_at | TM6SF1 | Transmembrane 6 superfamily member 1 | [50528](http://www.ncbi.nlm.nih.gov/UniGene/clust.cgi?ORG=Ssc&CID=50528) | 1.4 | 3.9 | 2.27E-03 | 4.30E-02 | -1.75 |
| Ssc.7996.1.A1_at | CRABP1 | Retinoic acid-binding protein I, cellular (CRABP-I) | [7996](http://www.ncbi.nlm.nih.gov/UniGene/clust.cgi?ORG=Ssc&CID=7996) | -1.6 | -3.9 | 2.28E-03 | 4.30E-02 | -1.75 |
| Ssc.8411.1.A1_at | ARHGAP26 | Rho-GTPase-activating protein 26 (Oligophrenin-1 like protei | [8411](http://www.ncbi.nlm.nih.gov/UniGene/clust.cgi?ORG=Ssc&CID=8411) | -2.1 | -3.9 | 2.33E-03 | 4.30E-02 | -1.78 |
| Ssc.15466.1.S1_at | RRAGD | Ras-related GTP binding D; Rag D protein [Homo sapiens] | [15466](http://www.ncbi.nlm.nih.gov/UniGene/clust.cgi?ORG=Ssc&CID=15466) | -1.2 | -3.9 | 2.35E-03 | 4.30E-02 | -1.79 |
| Ssc.26269.1.S1_at | ELL2 |  | [26269](http://www.ncbi.nlm.nih.gov/UniGene/clust.cgi?ORG=Ssc&CID=26269) | -1.7 | -3.9 | 2.36E-03 | 4.30E-02 | -1.79 |
| Ssc.27277.1.S1_at | TES | Testin (TESS) | [46797](http://www.ncbi.nlm.nih.gov/UniGene/clust.cgi?ORG=Ssc&CID=46797) | 1.5 | 3.9 | 2.40E-03 | 4.30E-02 | -1.81 |
| Ssc.10285.1.A1_at | C10orf76 |  | [10285](http://www.ncbi.nlm.nih.gov/UniGene/clust.cgi?ORG=Ssc&CID=10285) | -1.8 | -3.9 | 2.42E-03 | 4.30E-02 | -1.82 |
| Ssc.24539.1.A1_at | PCDH9 | Protocadherin 9 precursor | [24539](http://www.ncbi.nlm.nih.gov/UniGene/clust.cgi?ORG=Ssc&CID=24539) | -1.4 | -3.9 | 2.42E-03 | 4.30E-02 | -1.82 |
| Ssc.7272.1.A1_at | SERPINB2 | Plasminogen activator inhibitor-2 precursor (PAI-2) (Placent | [36723](http://www.ncbi.nlm.nih.gov/UniGene/clust.cgi?ORG=Ssc&CID=36723) | 3.6 | 3.9 | 2.45E-03 | 4.30E-02 | -1.83 |
| Ssc.21219.1.S1_at | PABPN1 | Polyadenylate-binding protein 2 (Poly(A)-binding protein 2) | [16011 /// 72354](http://www.ncbi.nlm.nih.gov/UniGene/clust.cgi?ORG=Ssc&CID=16011) | -1.3 | -3.9 | 2.46E-03 | 4.30E-02 | -1.83 |
| Ssc.8854.1.A1_at | ARHGAP27 | Rho GTPase activating protein 27 [Homo sapiens] | [8854](http://www.ncbi.nlm.nih.gov/UniGene/clust.cgi?ORG=Ssc&CID=8854) | 1.6 | 3.9 | 2.47E-03 | 4.30E-02 | -1.84 |
| Ssc.1086.1.A1_at | MFAP5 | Microfibrillar-associated protein 5 precursor (MFAP-5) (Micr | [1086](http://www.ncbi.nlm.nih.gov/UniGene/clust.cgi?ORG=Ssc&CID=1086) | 1.6 | 3.9 | 2.51E-03 | 4.40E-02 | -1.85 |
| Ssc.23827.1.A1_at | AGL | Glycogen debranching enzyme (Glycogen debrancher) [Includes: | [23827](http://www.ncbi.nlm.nih.gov/UniGene/clust.cgi?ORG=Ssc&CID=23827) | -1.3 | -3.9 | 2.54E-03 | 4.40E-02 | -1.86 |
| Ssc.12615.1.A1_at | ENDOD1 | Probable endonuclease KIAA0830 precursor | [1757](http://www.ncbi.nlm.nih.gov/UniGene/clust.cgi?ORG=Ssc&CID=1757) | 1.4 | 3.9 | 2.55E-03 | 4.40E-02 | -1.87 |
| Ssc.9311.1.A1_at | PHLDA1 | pleckstrin homology-like domain, family A, member 1; PQ-rich | [9311](http://www.ncbi.nlm.nih.gov/UniGene/clust.cgi?ORG=Ssc&CID=9311) | 1.3 | 3.8 | 2.65E-03 | 4.50E-02 | -1.91 |
| Ssc.204.1.S1_at | CYP3A4 | Cytochrome P450 3A4 (EC 1.14.13.67) (Quinine 3-monooxygenase | [204](http://www.ncbi.nlm.nih.gov/UniGene/clust.cgi?ORG=Ssc&CID=204) | -2.1 | -3.8 | 2.66E-03 | 4.50E-02 | -1.91 |
| Ssc.5227.1.S2_at | 0 | 2 kDa protein | [5227](http://www.ncbi.nlm.nih.gov/UniGene/clust.cgi?ORG=Ssc&CID=5227) | 1.8 | 3.8 | 2.74E-03 | 4.60E-02 | -1.94 |
| Ssc.8389.1.A1_at | FBXL2 | F-box/LRR-repeat protein 2 (F-box and leucine-rich repeat pr | [8389](http://www.ncbi.nlm.nih.gov/UniGene/clust.cgi?ORG=Ssc&CID=8389) | -1.5 | -3.8 | 2.75E-03 | 4.60E-02 | -1.95 |
| Ssc.23288.1.S1_at | 0 | 61 kDa protein | [---](http://www.ncbi.nlm.nih.gov/UniGene/clust.cgi?ORG=Ssc&CID=---) | -1.4 | -3.8 | 2.76E-03 | 4.60E-02 | -1.95 |
| Ssc.12329.2.S1_at | RGS5 | Regulator of G-protein signaling 5 (RGS5). | [44156](http://www.ncbi.nlm.nih.gov/UniGene/clust.cgi?ORG=Ssc&CID=44156) | 1.9 | 3.8 | 2.77E-03 | 4.60E-02 | -1.95 |
| Ssc.8980.1.A1_at | ANGPTL4 | Angiopoietin-related protein 4 precursor (Angiopoietin-like | [17345](http://www.ncbi.nlm.nih.gov/UniGene/clust.cgi?ORG=Ssc&CID=17345) | -1.5 | -3.8 | 2.79E-03 | 4.60E-02 | -1.96 |
| Ssc.30965.1.A1_at | DNAH14 | Novel protein (Fragment) | [44269](http://www.ncbi.nlm.nih.gov/UniGene/clust.cgi?ORG=Ssc&CID=44269) | -1.3 | -3.8 | 2.81E-03 | 4.60E-02 | -1.97 |
| Ssc.21256.1.S2_at | LIF | Leukemia inhibitory factor precursor (LIF) (Differentiation- | [52878](http://www.ncbi.nlm.nih.gov/UniGene/clust.cgi?ORG=Ssc&CID=52878) | 1.7 | 3.8 | 2.87E-03 | 4.60E-02 | -1.99 |
| Ssc.11421.2.A1_at | F2RL1 | Proteinase activated receptor 2 precursor (PAR-2) (Thrombin | [11421](http://www.ncbi.nlm.nih.gov/UniGene/clust.cgi?ORG=Ssc&CID=11421) | 2.1 | 3.8 | 2.90E-03 | 4.70E-02 | -2.00 |
| Ssc.629.1.S1_at | NPPB | Natriuretic peptides B precursor [Contains: Gamma-brain natr | [629](http://www.ncbi.nlm.nih.gov/UniGene/clust.cgi?ORG=Ssc&CID=629) | 1.9 | 3.8 | 2.92E-03 | 4.70E-02 | -2.01 |
| Ssc.22795.1.A1_at | RHOBTB3 | Rho-related BTB domain-containing protein 3 | [22795](http://www.ncbi.nlm.nih.gov/UniGene/clust.cgi?ORG=Ssc&CID=22795) | -1.4 | -3.8 | 2.98E-03 | 4.70E-02 | -2.03 |
| Ssc.2064.1.A1_at | GPR137C | G protein-coupled receptor TM7SF1L2 (Fragment) | [2064](http://www.ncbi.nlm.nih.gov/UniGene/clust.cgi?ORG=Ssc&CID=2064) | -1.4 | -3.8 | 3.00E-03 | 4.70E-02 | -2.04 |
| Ssc.5978.3.S1_a_at | RHBDD2 | rhomboid, veinlet-like 7; NPD007 protein [Homo sapiens] | [44112](http://www.ncbi.nlm.nih.gov/UniGene/clust.cgi?ORG=Ssc&CID=44112) | -1.3 | -3.8 | 3.00E-03 | 4.70E-02 | -2.04 |
| Ssc.16819.1.A1_at | ENC1 | Ectoderm-neural cortex-1 protein (ENC-1) (P53-induced protei | [74863](http://www.ncbi.nlm.nih.gov/UniGene/clust.cgi?ORG=Ssc&CID=74863) | 1.5 | 3.8 | 3.05E-03 | 4.70E-02 | -2.05 |
| Ssc.10642.1.S1_at | PPP1R9A |  | [10642](http://www.ncbi.nlm.nih.gov/UniGene/clust.cgi?ORG=Ssc&CID=10642) | 1.4 | 3.8 | 3.06E-03 | 4.70E-02 | -2.05 |
| Ssc.30914.1.S1_at | DDR2 | Discoidin domain receptor 2 precursor (EC 2.7.1.112) (Recept | [---](http://www.ncbi.nlm.nih.gov/UniGene/clust.cgi?ORG=Ssc&CID=---) | 1.3 | 3.8 | 3.08E-03 | 4.70E-02 | -2.06 |
| Ssc.24958.1.A1_at | CAV1 | Caveolin-1 | [57375](http://www.ncbi.nlm.nih.gov/UniGene/clust.cgi?ORG=Ssc&CID=57375) | -1.3 | -3.7 | 3.17E-03 | 4.80E-02 | -2.09 |
| Ssc.22398.1.A1_at | ETV6 | Transcription factor ETV6 (ETS-related protein Tel1) (Tel) | [22398](http://www.ncbi.nlm.nih.gov/UniGene/clust.cgi?ORG=Ssc&CID=22398) | -1.4 | -3.7 | 3.20E-03 | 4.80E-02 | -2.10 |
| Ssc.26230.1.S1_at | RRAD | GTP-binding protein RAD (RAS associated with diabetes) (RAD1 | [26230](http://www.ncbi.nlm.nih.gov/UniGene/clust.cgi?ORG=Ssc&CID=26230) | 1.3 | 3.7 | 3.22E-03 | 4.80E-02 | -2.11 |
| Ssc.18347.1.A1_s_at | Q9H5L9 |  | [18347](http://www.ncbi.nlm.nih.gov/UniGene/clust.cgi?ORG=Ssc&CID=18347) | 1.4 | 3.7 | 3.27E-03 | 4.80E-02 | -2.12 |
| Ssc.8004.1.A1_at | SLC16A12 | similar monocarboxylate transporter [Homo sapiens] | [8004](http://www.ncbi.nlm.nih.gov/UniGene/clust.cgi?ORG=Ssc&CID=8004) | 1.2 | 3.7 | 3.30E-03 | 4.80E-02 | -2.13 |
| Ssc.25009.1.A1_at | COL2A1 | Collagen alpha 1(II) chain precursor | [51118](http://www.ncbi.nlm.nih.gov/UniGene/clust.cgi?ORG=Ssc&CID=51118) | -1.6 | -3.7 | 3.32E-03 | 4.80E-02 | -2.14 |
| Ssc.15360.1.A1_a_at | ZNF512B | Transcription factor SOX-18. | [15360](http://www.ncbi.nlm.nih.gov/UniGene/clust.cgi?ORG=Ssc&CID=15360) | 1.4 | 3.7 | 3.32E-03 | 4.80E-02 | -2.14 |
| Ssc.9714.1.S1_at | LMO4 | LIM domain transcription factor LMO4 (LIM-only protein 4) (L | [9714](http://www.ncbi.nlm.nih.gov/UniGene/clust.cgi?ORG=Ssc&CID=9714) | -1.4 | -3.7 | 3.36E-03 | 4.80E-02 | -2.15 |
| Ssc.16927.1.S1_at | HTRA2 | Serine protease HTRA2, mitochondrial precursor (EC 3.4.21.-) | [55341](http://www.ncbi.nlm.nih.gov/UniGene/clust.cgi?ORG=Ssc&CID=55341) | -1.5 | -3.7 | 3.37E-03 | 4.80E-02 | -2.15 |
| Ssc.3804.3.S1_at | CYP27A1 | Cytochrome P450 27, mitochondrial precursor (EC 1.14.-.-) (C | [54372](http://www.ncbi.nlm.nih.gov/UniGene/clust.cgi?ORG=Ssc&CID=54372) | -1.3 | -3.7 | 3.38E-03 | 4.80E-02 | -2.16 |
| Ssc.12005.1.A1_at | BAT1 | Vacuolar ATP synthase subunit G 2 (EC 3.6.3.14) (V-ATPase G | [57219](http://www.ncbi.nlm.nih.gov/UniGene/clust.cgi?ORG=Ssc&CID=57219) | -1.7 | -3.7 | 3.38E-03 | 4.80E-02 | -2.16 |
| Ssc.27181.1.S1_at | AASDHPPT | aminoadipate-semialdehyde dehydrogenase-phosphopantetheinyl | [27181](http://www.ncbi.nlm.nih.gov/UniGene/clust.cgi?ORG=Ssc&CID=27181) | -1.5 | -3.7 | 3.39E-03 | 4.80E-02 | -2.16 |
| Ssc.12263.1.A1_at | SOX13 | SOX-13 protein (Type 1 diabetes autoantigen ICA12) (Islet ce | [12263](http://www.ncbi.nlm.nih.gov/UniGene/clust.cgi?ORG=Ssc&CID=12263) | 1.3 | 3.7 | 3.40E-03 | 4.80E-02 | -2.16 |
| Ssc.29302.1.A1_at | SLIT3 | Slit homolog 3 protein precursor (Slit-3) (Multiple epiderma | [40848](http://www.ncbi.nlm.nih.gov/UniGene/clust.cgi?ORG=Ssc&CID=40848) | -1.3 | -3.7 | 3.43E-03 | 4.80E-02 | -2.17 |
| Ssc.13714.1.A1_at | PTAR1 | PREDICTED: similar to PTAR1 protein | [13714](http://www.ncbi.nlm.nih.gov/UniGene/clust.cgi?ORG=Ssc&CID=13714) | -1.4 | -3.7 | 3.50E-03 | 4.90E-02 | -2.19 |
| Ssc.14477.1.S1_at | CILP | cartilage intermediate layer protein [Homo sapiens] | [14477](http://www.ncbi.nlm.nih.gov/UniGene/clust.cgi?ORG=Ssc&CID=14477) | -2 | -3.7 | 3.50E-03 | 4.90E-02 | -2.19 |
| Ssc.29799.1.A1_at | FAM22D | family with sequence similarity 22, member A | [29221](http://www.ncbi.nlm.nih.gov/UniGene/clust.cgi?ORG=Ssc&CID=29221) | 1.2 | 3.7 | 3.54E-03 | 4.90E-02 | -2.20 |
| Ssc.13801.1.A1_at | Q96GV6 |  | [13801](http://www.ncbi.nlm.nih.gov/UniGene/clust.cgi?ORG=Ssc&CID=13801) | -2.2 | -3.7 | 3.61E-03 | 5.00E-02 | -2.22 |
| Ssc.5995.1.A1_at | CRISPLD1 | CocoaCrisp [Homo sapiens] | [5995](http://www.ncbi.nlm.nih.gov/UniGene/clust.cgi?ORG=Ssc&CID=5995) | -1.2 | -3.7 | 3.62E-03 | 5.00E-02 | -2.22 |
| Ssc.17506.1.S1_at | NRXN1 |  | [17506](http://www.ncbi.nlm.nih.gov/UniGene/clust.cgi?ORG=Ssc&CID=17506) | 2.7 | 3.7 | 3.66E-03 | 5.00E-02 | -2.24 |
| Ssc.271.1.A1_at | CD55 | Complement decay-accelerating factor precursor (CD55 antigen | [272](http://www.ncbi.nlm.nih.gov/UniGene/clust.cgi?ORG=Ssc&CID=272) | 1.5 | 3.7 | 3.66E-03 | 5.00E-02 | -2.24 |
| Ssc.10300.1.S1_at | KLHL13 | Kelch-like protein 13 (BTB and kelch domain containing prote | [10300](http://www.ncbi.nlm.nih.gov/UniGene/clust.cgi?ORG=Ssc&CID=10300) | 1.3 | 3.7 | 3.70E-03 | 5.00E-02 | -2.25 |
| Ssc.19808.1.S1_at | SPINK5 | Serine protease inhibitor Kazal-type 5 precursor (Lympho-epi | [---](http://www.ncbi.nlm.nih.gov/UniGene/clust.cgi?ORG=Ssc&CID=---) | 1.6 | 3.7 | 3.71E-03 | 5.00E-02 | -2.25 |
| Ssc.16356.1.S1_at | COMP | Cartilage oligomeric matrix protein precursor (COMP) | [16356](http://www.ncbi.nlm.nih.gov/UniGene/clust.cgi?ORG=Ssc&CID=16356) | -1.9 | -3.7 | 3.72E-03 | 5.00E-02 | -2.25 |
| Ssc.18504.1.S1_at | RCAN2 | Calcipressin 2 (Thyroid hormone-responsive protein ZAKI-4) ( | [18504](http://www.ncbi.nlm.nih.gov/UniGene/clust.cgi?ORG=Ssc&CID=18504) | -1.1 | -3.6 | 3.74E-03 | 5.00E-02 | -2.26 |
| Ssc.3232.1.S1_at | SFRP2 | secreted frizzled-related protein 2 precursor; secreted apop | [---](http://www.ncbi.nlm.nih.gov/UniGene/clust.cgi?ORG=Ssc&CID=---) | 2.4 | 3.6 | 3.78E-03 | 5.00E-02 | -2.27 |
