# Supplementary material for: Sex-Related Differences in Gene Expression by Porcine Aortic Valvular Interstitial Cells
Source: PLoS One. 2012 Jul 10;7(7):e39980. doi: 10.1371/journal.pone.0039980 (PMC3393722; doi:10.1371/journal.pone.0039980)
Supplement: Table S3 — Significant biological processes determined by GO enrichment analysis. Expected count (Exp Count) refers to the number of differentially expressed genes that are predicted within the GO term tested, Count refers to the number of differentially expressed genes that were found within the GO term tested, and Size refers to the number of genes in the microarray that are listed within the GO term tested. (DOC) [file pone.0039980.s007.doc]

**Table S3**. Significant biological processes determined by GO enrichment analysis. Expected count (Exp Count) refers to the number of differentially expressed genes that are predicted within the GO term tested, Count refers to the number of differentially expressed genes that were found within the GO term tested, and Size refers to the number of genes in the microarray that are listed within the GO term tested.

| **GOBPID** | **P value** | **OddsRatio** | **Exp**  **Count** | **Count** | **Size** | **Term** |
| --- | --- | --- | --- | --- | --- | --- |
| GO:0030825 | 0 | 241.46 | 0 | 3 | 4 | [positive regulation of cGMP metabolic process](http://www.godatabase.org/cgi-bin/amigo/go.cgi?view=details&search_constraint=terms&depth=0&query=GO:0030825) |
| GO:0030828 | 0 | 241.46 | 0 | 3 | 4 | [positive regulation of cGMP biosynthetic process](http://www.godatabase.org/cgi-bin/amigo/go.cgi?view=details&search_constraint=terms&depth=0&query=GO:0030828) |
| GO:0040013 | 0 | 16.80 | 0 | 5 | 30 | [negative regulation of locomotion](http://www.godatabase.org/cgi-bin/amigo/go.cgi?view=details&search_constraint=terms&depth=0&query=GO:0040013) |
| GO:0030823 | 0 | 80.44 | 0 | 3 | 6 | [regulation of cGMP metabolic process](http://www.godatabase.org/cgi-bin/amigo/go.cgi?view=details&search_constraint=terms&depth=0&query=GO:0030823) |
| GO:0030826 | 0 | 80.44 | 0 | 3 | 6 | [regulation of cGMP biosynthetic process](http://www.godatabase.org/cgi-bin/amigo/go.cgi?view=details&search_constraint=terms&depth=0&query=GO:0030826) |
| GO:0019932 | 0 | 8.14 | 1 | 7 | 82 | [second-messenger-mediated signaling](http://www.godatabase.org/cgi-bin/amigo/go.cgi?view=details&search_constraint=terms&depth=0&query=GO:0019932) |
| GO:0001890 | 0 | 9.87 | 1 | 6 | 58 | [placenta development](http://www.godatabase.org/cgi-bin/amigo/go.cgi?view=details&search_constraint=terms&depth=0&query=GO:0001890) |
| GO:0048519 | 0 | 3.30 | 11 | 23 | 846 | [negative regulation of biological process](http://www.godatabase.org/cgi-bin/amigo/go.cgi?view=details&search_constraint=terms&depth=0&query=GO:0048519) |
| GO:0043569 | 0 | Inf | 0 | 2 | 2 | [negative regulation of insulin-like growth factor receptor signaling pathway](http://www.godatabase.org/cgi-bin/amigo/go.cgi?view=details&search_constraint=terms&depth=0&query=GO:0043569) |
| GO:0006182 | 0 | 34.43 | 0 | 3 | 10 | [cGMP biosynthetic process](http://www.godatabase.org/cgi-bin/amigo/go.cgi?view=details&search_constraint=terms&depth=0&query=GO:0006182) |
| GO:0030336 | 0 | 15.62 | 0 | 4 | 25 | [negative regulation of cell migration](http://www.godatabase.org/cgi-bin/amigo/go.cgi?view=details&search_constraint=terms&depth=0&query=GO:0030336) |
| GO:0048523 | 0 | 3.07 | 10 | 20 | 724 | [negative regulation of cellular process](http://www.godatabase.org/cgi-bin/amigo/go.cgi?view=details&search_constraint=terms&depth=0&query=GO:0048523) |
| GO:0033002 | 0 | 7.51 | 1 | 6 | 74 | [muscle cell proliferation](http://www.godatabase.org/cgi-bin/amigo/go.cgi?view=details&search_constraint=terms&depth=0&query=GO:0033002) |
| GO:0051271 | 0 | 14.25 | 0 | 4 | 27 | [negative regulation of cellular component movement](http://www.godatabase.org/cgi-bin/amigo/go.cgi?view=details&search_constraint=terms&depth=0&query=GO:0051271) |
| GO:0003416 | 0.001 | 157.14 | 0 | 2 | 3 | [endochondral bone growth](http://www.godatabase.org/cgi-bin/amigo/go.cgi?view=details&search_constraint=terms&depth=0&query=GO:0003416) |
| GO:0003417 | 0.001 | 157.14 | 0 | 2 | 3 | [growth plate cartilage development](http://www.godatabase.org/cgi-bin/amigo/go.cgi?view=details&search_constraint=terms&depth=0&query=GO:0003417) |
| GO:0019935 | 0.001 | 8.69 | 1 | 5 | 53 | [cyclic-nucleotide-mediated signaling](http://www.godatabase.org/cgi-bin/amigo/go.cgi?view=details&search_constraint=terms&depth=0&query=GO:0019935) |
| GO:0001503 | 0.001 | 5.65 | 1 | 7 | 114 | [ossification](http://www.godatabase.org/cgi-bin/amigo/go.cgi?view=details&search_constraint=terms&depth=0&query=GO:0001503) |
| GO:0030182 | 0.001 | 3.64 | 4 | 12 | 320 | [neuron differentiation](http://www.godatabase.org/cgi-bin/amigo/go.cgi?view=details&search_constraint=terms&depth=0&query=GO:0030182) |
| GO:0030801 | 0.001 | 21.89 | 0 | 3 | 14 | [positive regulation of cyclic nucleotide metabolic process](http://www.godatabase.org/cgi-bin/amigo/go.cgi?view=details&search_constraint=terms&depth=0&query=GO:0030801) |
| GO:0030804 | 0.001 | 21.89 | 0 | 3 | 14 | [positive regulation of cyclic nucleotide biosynthetic process](http://www.godatabase.org/cgi-bin/amigo/go.cgi?view=details&search_constraint=terms&depth=0&query=GO:0030804) |
| GO:0030810 | 0.001 | 21.89 | 0 | 3 | 14 | [positive regulation of nucleotide biosynthetic process](http://www.godatabase.org/cgi-bin/amigo/go.cgi?view=details&search_constraint=terms&depth=0&query=GO:0030810) |
| GO:0045981 | 0.001 | 21.89 | 0 | 3 | 14 | [positive regulation of nucleotide metabolic process](http://www.godatabase.org/cgi-bin/amigo/go.cgi?view=details&search_constraint=terms&depth=0&query=GO:0045981) |
| GO:0007166 | 0.001 | 3.16 | 6 | 15 | 479 | [cell surface receptor linked signaling pathway](http://www.godatabase.org/cgi-bin/amigo/go.cgi?view=details&search_constraint=terms&depth=0&query=GO:0007166) |
| GO:0048659 | 0.001 | 8.01 | 1 | 5 | 57 | [smooth muscle cell proliferation](http://www.godatabase.org/cgi-bin/amigo/go.cgi?view=details&search_constraint=terms&depth=0&query=GO:0048659) |
| GO:0048660 | 0.001 | 8.01 | 1 | 5 | 57 | [regulation of smooth muscle cell proliferation](http://www.godatabase.org/cgi-bin/amigo/go.cgi?view=details&search_constraint=terms&depth=0&query=GO:0048660) |
| GO:0006706 | 0.001 | 20.06 | 0 | 3 | 15 | [steroid catabolic process](http://www.godatabase.org/cgi-bin/amigo/go.cgi?view=details&search_constraint=terms&depth=0&query=GO:0006706) |
| GO:0046068 | 0.001 | 20.06 | 0 | 3 | 15 | [cGMP metabolic process](http://www.godatabase.org/cgi-bin/amigo/go.cgi?view=details&search_constraint=terms&depth=0&query=GO:0046068) |
| GO:0030855 | 0.001 | 6.05 | 1 | 6 | 90 | [epithelial cell differentiation](http://www.godatabase.org/cgi-bin/amigo/go.cgi?view=details&search_constraint=terms&depth=0&query=GO:0030855) |
| GO:0007168 | 0.001 | 78.55 | 0 | 2 | 4 | [receptor guanylyl cyclase signaling pathway](http://www.godatabase.org/cgi-bin/amigo/go.cgi?view=details&search_constraint=terms&depth=0&query=GO:0007168) |
| GO:0009822 | 0.001 | 78.55 | 0 | 2 | 4 | [alkaloid catabolic process](http://www.godatabase.org/cgi-bin/amigo/go.cgi?view=details&search_constraint=terms&depth=0&query=GO:0009822) |
| GO:0009888 | 0.001 | 3.04 | 7 | 15 | 495 | [tissue development](http://www.godatabase.org/cgi-bin/amigo/go.cgi?view=details&search_constraint=terms&depth=0&query=GO:0009888) |
| GO:0048699 | 0.001 | 3.28 | 5 | 12 | 351 | [generation of neurons](http://www.godatabase.org/cgi-bin/amigo/go.cgi?view=details&search_constraint=terms&depth=0&query=GO:0048699) |
| GO:0019748 | 0.002 | 9.33 | 1 | 4 | 39 | [secondary metabolic process](http://www.godatabase.org/cgi-bin/amigo/go.cgi?view=details&search_constraint=terms&depth=0&query=GO:0019748) |
| GO:0030802 | 0.002 | 6.81 | 1 | 5 | 66 | [regulation of cyclic nucleotide biosynthetic process](http://www.godatabase.org/cgi-bin/amigo/go.cgi?view=details&search_constraint=terms&depth=0&query=GO:0030802) |
| GO:0030808 | 0.002 | 6.81 | 1 | 5 | 66 | [regulation of nucleotide biosynthetic process](http://www.godatabase.org/cgi-bin/amigo/go.cgi?view=details&search_constraint=terms&depth=0&query=GO:0030808) |
| GO:0040012 | 0.002 | 4.69 | 2 | 7 | 135 | [regulation of locomotion](http://www.godatabase.org/cgi-bin/amigo/go.cgi?view=details&search_constraint=terms&depth=0&query=GO:0040012) |
| GO:0030799 | 0.002 | 6.70 | 1 | 5 | 67 | [regulation of cyclic nucleotide metabolic process](http://www.godatabase.org/cgi-bin/amigo/go.cgi?view=details&search_constraint=terms&depth=0&query=GO:0030799) |
| GO:0060712 | 0.002 | 52.35 | 0 | 2 | 5 | [spongiotrophoblast layer development](http://www.godatabase.org/cgi-bin/amigo/go.cgi?view=details&search_constraint=terms&depth=0&query=GO:0060712) |
| GO:0048513 | 0.002 | 2.57 | 14 | 24 | 1076 | [organ development](http://www.godatabase.org/cgi-bin/amigo/go.cgi?view=details&search_constraint=terms&depth=0&query=GO:0048513) |
| GO:0051239 | 0.002 | 2.75 | 8 | 17 | 632 | [regulation of multicellular organismal process](http://www.godatabase.org/cgi-bin/amigo/go.cgi?view=details&search_constraint=terms&depth=0&query=GO:0051239) |
| GO:0009190 | 0.002 | 6.28 | 1 | 5 | 71 | [cyclic nucleotide biosynthetic process](http://www.godatabase.org/cgi-bin/amigo/go.cgi?view=details&search_constraint=terms&depth=0&query=GO:0009190) |
| GO:0048731 | 0.002 | 2.49 | 16 | 26 | 1238 | [system development](http://www.godatabase.org/cgi-bin/amigo/go.cgi?view=details&search_constraint=terms&depth=0&query=GO:0048731) |
| GO:0015870 | 0.002 | 39.25 | 0 | 2 | 6 | [acetylcholine transport](http://www.godatabase.org/cgi-bin/amigo/go.cgi?view=details&search_constraint=terms&depth=0&query=GO:0015870) |
| GO:0060351 | 0.002 | 39.25 | 0 | 2 | 6 | [cartilage development involved in endochondral bone morphogenesis](http://www.godatabase.org/cgi-bin/amigo/go.cgi?view=details&search_constraint=terms&depth=0&query=GO:0060351) |
| GO:0031328 | 0.003 | 3.15 | 4 | 11 | 327 | [positive regulation of cellular biosynthetic process](http://www.godatabase.org/cgi-bin/amigo/go.cgi?view=details&search_constraint=terms&depth=0&query=GO:0031328) |
| GO:0022008 | 0.003 | 3.01 | 5 | 12 | 378 | [neurogenesis](http://www.godatabase.org/cgi-bin/amigo/go.cgi?view=details&search_constraint=terms&depth=0&query=GO:0022008) |
| GO:0030278 | 0.003 | 7.76 | 1 | 4 | 46 | [regulation of ossification](http://www.godatabase.org/cgi-bin/amigo/go.cgi?view=details&search_constraint=terms&depth=0&query=GO:0030278) |
| GO:0009124 | 0.003 | 5.75 | 1 | 5 | 77 | [nucleoside monophosphate biosynthetic process](http://www.godatabase.org/cgi-bin/amigo/go.cgi?view=details&search_constraint=terms&depth=0&query=GO:0009124) |
| GO:0006721 | 0.003 | 12.00 | 0 | 3 | 23 | [terpenoid metabolic process](http://www.godatabase.org/cgi-bin/amigo/go.cgi?view=details&search_constraint=terms&depth=0&query=GO:0006721) |
| GO:0001944 | 0.003 | 3.72 | 3 | 8 | 194 | [vasculature development](http://www.godatabase.org/cgi-bin/amigo/go.cgi?view=details&search_constraint=terms&depth=0&query=GO:0001944) |
| GO:0048856 | 0.003 | 2.39 | 18 | 27 | 1344 | [anatomical structure development](http://www.godatabase.org/cgi-bin/amigo/go.cgi?view=details&search_constraint=terms&depth=0&query=GO:0048856) |
| GO:0009891 | 0.003 | 3.02 | 4 | 11 | 339 | [positive regulation of biosynthetic process](http://www.godatabase.org/cgi-bin/amigo/go.cgi?view=details&search_constraint=terms&depth=0&query=GO:0009891) |
| GO:0030334 | 0.004 | 4.62 | 2 | 6 | 115 | [regulation of cell migration](http://www.godatabase.org/cgi-bin/amigo/go.cgi?view=details&search_constraint=terms&depth=0&query=GO:0030334) |
| GO:0007167 | 0.004 | 3.66 | 3 | 8 | 197 | [enzyme linked receptor protein signaling pathway](http://www.godatabase.org/cgi-bin/amigo/go.cgi?view=details&search_constraint=terms&depth=0&query=GO:0007167) |
| GO:0042127 | 0.004 | 2.87 | 5 | 12 | 393 | [regulation of cell proliferation](http://www.godatabase.org/cgi-bin/amigo/go.cgi?view=details&search_constraint=terms&depth=0&query=GO:0042127) |
| GO:0009187 | 0.004 | 5.44 | 1 | 5 | 81 | [cyclic nucleotide metabolic process](http://www.godatabase.org/cgi-bin/amigo/go.cgi?view=details&search_constraint=terms&depth=0&query=GO:0009187) |
| GO:0032501 | 0.004 | 2.54 | 24 | 33 | 1822 | [multicellular organismal process](http://www.godatabase.org/cgi-bin/amigo/go.cgi?view=details&search_constraint=terms&depth=0&query=GO:0032501) |
| GO:0031103 | 0.004 | 10.91 | 0 | 3 | 25 | [axon regeneration](http://www.godatabase.org/cgi-bin/amigo/go.cgi?view=details&search_constraint=terms&depth=0&query=GO:0031103) |
| GO:0060560 | 0.004 | 10.91 | 0 | 3 | 25 | [developmental growth involved in morphogenesis](http://www.godatabase.org/cgi-bin/amigo/go.cgi?view=details&search_constraint=terms&depth=0&query=GO:0060560) |
| GO:0045935 | 0.004 | 3.11 | 4 | 10 | 295 | [positive regulation of nucleobase, nucleoside, nucleotide and nucleic acid metabolic process](http://www.godatabase.org/cgi-bin/amigo/go.cgi?view=details&search_constraint=terms&depth=0&query=GO:0045935) |
| GO:0048514 | 0.004 | 3.89 | 2 | 7 | 160 | [blood vessel morphogenesis](http://www.godatabase.org/cgi-bin/amigo/go.cgi?view=details&search_constraint=terms&depth=0&query=GO:0048514) |
| GO:0031099 | 0.005 | 4.30 | 2 | 6 | 123 | [regeneration](http://www.godatabase.org/cgi-bin/amigo/go.cgi?view=details&search_constraint=terms&depth=0&query=GO:0031099) |
| GO:0031102 | 0.005 | 9.99 | 0 | 3 | 27 | [neuron projection regeneration](http://www.godatabase.org/cgi-bin/amigo/go.cgi?view=details&search_constraint=terms&depth=0&query=GO:0031102) |
| GO:0032502 | 0.005 | 2.36 | 21 | 30 | 1602 | [developmental process](http://www.godatabase.org/cgi-bin/amigo/go.cgi?view=details&search_constraint=terms&depth=0&query=GO:0032502) |
| GO:0051270 | 0.005 | 4.26 | 2 | 6 | 124 | [regulation of cellular component movement](http://www.godatabase.org/cgi-bin/amigo/go.cgi?view=details&search_constraint=terms&depth=0&query=GO:0051270) |
| GO:0051173 | 0.005 | 2.98 | 4 | 10 | 307 | [positive regulation of nitrogen compound metabolic process](http://www.godatabase.org/cgi-bin/amigo/go.cgi?view=details&search_constraint=terms&depth=0&query=GO:0051173) |
| GO:0023033 | 0.006 | 2.36 | 10 | 18 | 766 | [signaling pathway](http://www.godatabase.org/cgi-bin/amigo/go.cgi?view=details&search_constraint=terms&depth=0&query=GO:0023033) |
| GO:0009123 | 0.006 | 4.91 | 1 | 5 | 89 | [nucleoside monophosphate metabolic process](http://www.godatabase.org/cgi-bin/amigo/go.cgi?view=details&search_constraint=terms&depth=0&query=GO:0009123) |
| GO:0009820 | 0.006 | 22.41 | 0 | 2 | 9 | [alkaloid metabolic process](http://www.godatabase.org/cgi-bin/amigo/go.cgi?view=details&search_constraint=terms&depth=0&query=GO:0009820) |
| GO:0016098 | 0.006 | 22.41 | 0 | 2 | 9 | [monoterpenoid metabolic process](http://www.godatabase.org/cgi-bin/amigo/go.cgi?view=details&search_constraint=terms&depth=0&query=GO:0016098) |
| GO:0070989 | 0.006 | 22.41 | 0 | 2 | 9 | [oxidative demethylation](http://www.godatabase.org/cgi-bin/amigo/go.cgi?view=details&search_constraint=terms&depth=0&query=GO:0070989) |
| GO:0009893 | 0.006 | 2.58 | 6 | 13 | 474 | [positive regulation of metabolic process](http://www.godatabase.org/cgi-bin/amigo/go.cgi?view=details&search_constraint=terms&depth=0&query=GO:0009893) |
| GO:0023052 | 0.006 | 2.27 | 13 | 21 | 969 | [signaling](http://www.godatabase.org/cgi-bin/amigo/go.cgi?view=details&search_constraint=terms&depth=0&query=GO:0023052) |
| GO:0007275 | 0.006 | 2.26 | 19 | 28 | 1468 | [multicellular organismal development](http://www.godatabase.org/cgi-bin/amigo/go.cgi?view=details&search_constraint=terms&depth=0&query=GO:0007275) |
| GO:0032101 | 0.006 | 4.05 | 2 | 6 | 130 | [regulation of response to external stimulus](http://www.godatabase.org/cgi-bin/amigo/go.cgi?view=details&search_constraint=terms&depth=0&query=GO:0032101) |
| GO:0043542 | 0.007 | 8.87 | 0 | 3 | 30 | [endothelial cell migration](http://www.godatabase.org/cgi-bin/amigo/go.cgi?view=details&search_constraint=terms&depth=0&query=GO:0043542) |
| GO:0050731 | 0.007 | 8.87 | 0 | 3 | 30 | [positive regulation of peptidyl-tyrosine phosphorylation](http://www.godatabase.org/cgi-bin/amigo/go.cgi?view=details&search_constraint=terms&depth=0&query=GO:0050731) |
| GO:0042738 | 0.007 | 19.60 | 0 | 2 | 10 | [exogenous drug catabolic process](http://www.godatabase.org/cgi-bin/amigo/go.cgi?view=details&search_constraint=terms&depth=0&query=GO:0042738) |
| GO:0043567 | 0.007 | 19.60 | 0 | 2 | 10 | [regulation of insulin-like growth factor receptor signaling pathway](http://www.godatabase.org/cgi-bin/amigo/go.cgi?view=details&search_constraint=terms&depth=0&query=GO:0043567) |
| GO:0040011 | 0.007 | 2.72 | 5 | 11 | 372 | [locomotion](http://www.godatabase.org/cgi-bin/amigo/go.cgi?view=details&search_constraint=terms&depth=0&query=GO:0040011) |
| GO:0016337 | 0.008 | 4.57 | 1 | 5 | 95 | [cell-cell adhesion](http://www.godatabase.org/cgi-bin/amigo/go.cgi?view=details&search_constraint=terms&depth=0&query=GO:0016337) |
| GO:0007399 | 0.008 | 2.44 | 7 | 14 | 544 | [nervous system development](http://www.godatabase.org/cgi-bin/amigo/go.cgi?view=details&search_constraint=terms&depth=0&query=GO:0007399) |
| GO:0006140 | 0.008 | 4.52 | 1 | 5 | 96 | [regulation of nucleotide metabolic process](http://www.godatabase.org/cgi-bin/amigo/go.cgi?view=details&search_constraint=terms&depth=0&query=GO:0006140) |
| GO:0008283 | 0.008 | 2.50 | 6 | 13 | 488 | [cell proliferation](http://www.godatabase.org/cgi-bin/amigo/go.cgi?view=details&search_constraint=terms&depth=0&query=GO:0008283) |
| GO:0051093 | 0.008 | 3.85 | 2 | 6 | 136 | [negative regulation of developmental process](http://www.godatabase.org/cgi-bin/amigo/go.cgi?view=details&search_constraint=terms&depth=0&query=GO:0051093) |
| GO:0010038 | 0.008 | 3.40 | 2 | 7 | 181 | [response to metal ion](http://www.godatabase.org/cgi-bin/amigo/go.cgi?view=details&search_constraint=terms&depth=0&query=GO:0010038) |
| GO:0042737 | 0.009 | 17.42 | 0 | 2 | 11 | [drug catabolic process](http://www.godatabase.org/cgi-bin/amigo/go.cgi?view=details&search_constraint=terms&depth=0&query=GO:0042737) |
| GO:0016477 | 0.009 | 2.90 | 4 | 9 | 278 | [cell migration](http://www.godatabase.org/cgi-bin/amigo/go.cgi?view=details&search_constraint=terms&depth=0&query=GO:0016477) |
| GO:0003013 | 0.009 | 3.36 | 2 | 7 | 183 | [circulatory system process](http://www.godatabase.org/cgi-bin/amigo/go.cgi?view=details&search_constraint=terms&depth=0&query=GO:0003013) |
| GO:0008015 | 0.009 | 3.36 | 2 | 7 | 183 | [blood circulation](http://www.godatabase.org/cgi-bin/amigo/go.cgi?view=details&search_constraint=terms&depth=0&query=GO:0008015) |
| GO:0046483 | 0.009 | 3.08 | 3 | 8 | 230 | [heterocycle metabolic process](http://www.godatabase.org/cgi-bin/amigo/go.cgi?view=details&search_constraint=terms&depth=0&query=GO:0046483) |
| GO:0001892 | 0.01 | 7.72 | 0 | 3 | 34 | [embryonic placenta development](http://www.godatabase.org/cgi-bin/amigo/go.cgi?view=details&search_constraint=terms&depth=0&query=GO:0001892) |
| GO:0006720 | 0.01 | 7.72 | 0 | 3 | 34 | [isoprenoid metabolic process](http://www.godatabase.org/cgi-bin/amigo/go.cgi?view=details&search_constraint=terms&depth=0&query=GO:0006720) |
| GO:0001568 | 0.01 | 3.30 | 2 | 7 | 186 | [blood vessel development](http://www.godatabase.org/cgi-bin/amigo/go.cgi?view=details&search_constraint=terms&depth=0&query=GO:0001568) |
| GO:0048870 | 0.01 | 2.83 | 4 | 9 | 284 | [cell motility](http://www.godatabase.org/cgi-bin/amigo/go.cgi?view=details&search_constraint=terms&depth=0&query=GO:0048870) |
| GO:0048844 | 0.01 | 15.67 | 0 | 2 | 12 | [artery morphogenesis](http://www.godatabase.org/cgi-bin/amigo/go.cgi?view=details&search_constraint=terms&depth=0&query=GO:0048844) |
| GO:0051674 | 0.01 | 2.82 | 4 | 9 | 285 | [localization of cell](http://www.godatabase.org/cgi-bin/amigo/go.cgi?view=details&search_constraint=terms&depth=0&query=GO:0051674) |
| GO:0031325 | 0.011 | 2.46 | 6 | 12 | 448 | [positive regulation of cellular metabolic process](http://www.godatabase.org/cgi-bin/amigo/go.cgi?view=details&search_constraint=terms&depth=0&query=GO:0031325) |
| GO:0032103 | 0.011 | 5.14 | 1 | 4 | 67 | [positive regulation of response to external stimulus](http://www.godatabase.org/cgi-bin/amigo/go.cgi?view=details&search_constraint=terms&depth=0&query=GO:0032103) |
| GO:0030154 | 0.011 | 2.16 | 11 | 18 | 818 | [cell differentiation](http://www.godatabase.org/cgi-bin/amigo/go.cgi?view=details&search_constraint=terms&depth=0&query=GO:0030154) |
| GO:0044270 | 0.012 | 5.06 | 1 | 4 | 68 | [cellular nitrogen compound catabolic process](http://www.godatabase.org/cgi-bin/amigo/go.cgi?view=details&search_constraint=terms&depth=0&query=GO:0044270) |
| GO:0009605 | 0.012 | 2.42 | 6 | 12 | 455 | [response to external stimulus](http://www.godatabase.org/cgi-bin/amigo/go.cgi?view=details&search_constraint=terms&depth=0&query=GO:0009605) |
| GO:0016525 | 0.012 | 14.24 | 0 | 2 | 13 | [negative regulation of angiogenesis](http://www.godatabase.org/cgi-bin/amigo/go.cgi?view=details&search_constraint=terms&depth=0&query=GO:0016525) |
| GO:0046697 | 0.012 | 14.24 | 0 | 2 | 13 | [decidualization](http://www.godatabase.org/cgi-bin/amigo/go.cgi?view=details&search_constraint=terms&depth=0&query=GO:0046697) |
| GO:0048662 | 0.012 | 14.24 | 0 | 2 | 13 | [negative regulation of smooth muscle cell proliferation](http://www.godatabase.org/cgi-bin/amigo/go.cgi?view=details&search_constraint=terms&depth=0&query=GO:0048662) |
| GO:0060350 | 0.012 | 14.24 | 0 | 2 | 13 | [endochondral bone morphogenesis](http://www.godatabase.org/cgi-bin/amigo/go.cgi?view=details&search_constraint=terms&depth=0&query=GO:0060350) |
| GO:0060840 | 0.012 | 14.24 | 0 | 2 | 13 | [artery development](http://www.godatabase.org/cgi-bin/amigo/go.cgi?view=details&search_constraint=terms&depth=0&query=GO:0060840) |
| GO:0001649 | 0.012 | 7.03 | 0 | 3 | 37 | [osteoblast differentiation](http://www.godatabase.org/cgi-bin/amigo/go.cgi?view=details&search_constraint=terms&depth=0&query=GO:0001649) |
| GO:0060191 | 0.012 | 7.03 | 0 | 3 | 37 | [regulation of lipase activity](http://www.godatabase.org/cgi-bin/amigo/go.cgi?view=details&search_constraint=terms&depth=0&query=GO:0060191) |
| GO:0003157 | 0.013 | Inf | 0 | 1 | 1 | [endocardium development](http://www.godatabase.org/cgi-bin/amigo/go.cgi?view=details&search_constraint=terms&depth=0&query=GO:0003157) |
| GO:0003160 | 0.013 | Inf | 0 | 1 | 1 | [endocardium morphogenesis](http://www.godatabase.org/cgi-bin/amigo/go.cgi?view=details&search_constraint=terms&depth=0&query=GO:0003160) |
| GO:0003339 | 0.013 | Inf | 0 | 1 | 1 | [regulation of mesenchymal to epithelial transition involved in metanephros morphogenesis](http://www.godatabase.org/cgi-bin/amigo/go.cgi?view=details&search_constraint=terms&depth=0&query=GO:0003339) |
| GO:0003340 | 0.013 | Inf | 0 | 1 | 1 | [negative regulation of mesenchymal to epithelial transition involved in metanephros morphogenesis](http://www.godatabase.org/cgi-bin/amigo/go.cgi?view=details&search_constraint=terms&depth=0&query=GO:0003340) |
| GO:0003348 | 0.013 | Inf | 0 | 1 | 1 | [cardiac endothelial cell differentiation](http://www.godatabase.org/cgi-bin/amigo/go.cgi?view=details&search_constraint=terms&depth=0&query=GO:0003348) |
| GO:0003413 | 0.013 | Inf | 0 | 1 | 1 | [chondrocyte differentiation involved in endochondral bone morphogenesis](http://www.godatabase.org/cgi-bin/amigo/go.cgi?view=details&search_constraint=terms&depth=0&query=GO:0003413) |
| GO:0003418 | 0.013 | Inf | 0 | 1 | 1 | [growth plate cartilage chondrocyte differentiation](http://www.godatabase.org/cgi-bin/amigo/go.cgi?view=details&search_constraint=terms&depth=0&query=GO:0003418) |
| GO:0003419 | 0.013 | Inf | 0 | 1 | 1 | [growth plate cartilage chondrocyte proliferation](http://www.godatabase.org/cgi-bin/amigo/go.cgi?view=details&search_constraint=terms&depth=0&query=GO:0003419) |
| GO:0007114 | 0.013 | Inf | 0 | 1 | 1 | [cell budding](http://www.godatabase.org/cgi-bin/amigo/go.cgi?view=details&search_constraint=terms&depth=0&query=GO:0007114) |
| GO:0010716 | 0.013 | Inf | 0 | 1 | 1 | [negative regulation of extracellular matrix disassembly](http://www.godatabase.org/cgi-bin/amigo/go.cgi?view=details&search_constraint=terms&depth=0&query=GO:0010716) |
| GO:0032505 | 0.013 | Inf | 0 | 1 | 1 | [reproduction of a single-celled organism](http://www.godatabase.org/cgi-bin/amigo/go.cgi?view=details&search_constraint=terms&depth=0&query=GO:0032505) |
| GO:0032805 | 0.013 | Inf | 0 | 1 | 1 | [positive regulation of low-density lipoprotein receptor catabolic process](http://www.godatabase.org/cgi-bin/amigo/go.cgi?view=details&search_constraint=terms&depth=0&query=GO:0032805) |
| GO:0033632 | 0.013 | Inf | 0 | 1 | 1 | [regulation of cell-cell adhesion mediated by integrin](http://www.godatabase.org/cgi-bin/amigo/go.cgi?view=details&search_constraint=terms&depth=0&query=GO:0033632) |
| GO:0051497 | 0.013 | Inf | 0 | 1 | 1 | [negative regulation of stress fiber assembly](http://www.godatabase.org/cgi-bin/amigo/go.cgi?view=details&search_constraint=terms&depth=0&query=GO:0051497) |
| GO:0060214 | 0.013 | Inf | 0 | 1 | 1 | [endocardium formation](http://www.godatabase.org/cgi-bin/amigo/go.cgi?view=details&search_constraint=terms&depth=0&query=GO:0060214) |
| GO:0060956 | 0.013 | Inf | 0 | 1 | 1 | [endocardial cell differentiation](http://www.godatabase.org/cgi-bin/amigo/go.cgi?view=details&search_constraint=terms&depth=0&query=GO:0060956) |
| GO:0071104 | 0.013 | Inf | 0 | 1 | 1 | [response to interleukin-9](http://www.godatabase.org/cgi-bin/amigo/go.cgi?view=details&search_constraint=terms&depth=0&query=GO:0071104) |
| GO:0071105 | 0.013 | Inf | 0 | 1 | 1 | [response to interleukin-11](http://www.godatabase.org/cgi-bin/amigo/go.cgi?view=details&search_constraint=terms&depth=0&query=GO:0071105) |
| GO:0071603 | 0.013 | Inf | 0 | 1 | 1 | [endothelial cell-cell adhesion](http://www.godatabase.org/cgi-bin/amigo/go.cgi?view=details&search_constraint=terms&depth=0&query=GO:0071603) |
| GO:0048869 | 0.014 | 2.11 | 11 | 18 | 833 | [cellular developmental process](http://www.godatabase.org/cgi-bin/amigo/go.cgi?view=details&search_constraint=terms&depth=0&query=GO:0048869) |
| GO:0001701 | 0.014 | 3.90 | 1 | 5 | 110 | [in utero embryonic development](http://www.godatabase.org/cgi-bin/amigo/go.cgi?view=details&search_constraint=terms&depth=0&query=GO:0001701) |
| GO:0051172 | 0.014 | 3.06 | 3 | 7 | 199 | [negative regulation of nitrogen compound metabolic process](http://www.godatabase.org/cgi-bin/amigo/go.cgi?view=details&search_constraint=terms&depth=0&query=GO:0051172) |
| GO:0006163 | 0.014 | 3.39 | 2 | 6 | 153 | [purine nucleotide metabolic process](http://www.godatabase.org/cgi-bin/amigo/go.cgi?view=details&search_constraint=terms&depth=0&query=GO:0006163) |
| GO:0048666 | 0.014 | 2.83 | 3 | 8 | 248 | [neuron development](http://www.godatabase.org/cgi-bin/amigo/go.cgi?view=details&search_constraint=terms&depth=0&query=GO:0048666) |
| GO:0048009 | 0.014 | 13.05 | 0 | 2 | 14 | [insulin-like growth factor receptor signaling pathway](http://www.godatabase.org/cgi-bin/amigo/go.cgi?view=details&search_constraint=terms&depth=0&query=GO:0048009) |
| GO:0070988 | 0.014 | 13.05 | 0 | 2 | 14 | [demethylation](http://www.godatabase.org/cgi-bin/amigo/go.cgi?view=details&search_constraint=terms&depth=0&query=GO:0070988) |
| GO:0032879 | 0.014 | 2.44 | 5 | 11 | 408 | [regulation of localization](http://www.godatabase.org/cgi-bin/amigo/go.cgi?view=details&search_constraint=terms&depth=0&query=GO:0032879) |
| GO:0006164 | 0.014 | 3.86 | 1 | 5 | 111 | [purine nucleotide biosynthetic process](http://www.godatabase.org/cgi-bin/amigo/go.cgi?view=details&search_constraint=terms&depth=0&query=GO:0006164) |
| GO:0008285 | 0.015 | 3.34 | 2 | 6 | 155 | [negative regulation of cell proliferation](http://www.godatabase.org/cgi-bin/amigo/go.cgi?view=details&search_constraint=terms&depth=0&query=GO:0008285) |
| GO:0009887 | 0.015 | 2.41 | 5 | 11 | 412 | [organ morphogenesis](http://www.godatabase.org/cgi-bin/amigo/go.cgi?view=details&search_constraint=terms&depth=0&query=GO:0009887) |
| GO:0001893 | 0.016 | 12.04 | 0 | 2 | 15 | [maternal placenta development](http://www.godatabase.org/cgi-bin/amigo/go.cgi?view=details&search_constraint=terms&depth=0&query=GO:0001893) |
| GO:0050730 | 0.016 | 6.28 | 1 | 3 | 41 | [regulation of peptidyl-tyrosine phosphorylation](http://www.godatabase.org/cgi-bin/amigo/go.cgi?view=details&search_constraint=terms&depth=0&query=GO:0050730) |
| GO:0019722 | 0.018 | 11.18 | 0 | 2 | 16 | [calcium-mediated signaling](http://www.godatabase.org/cgi-bin/amigo/go.cgi?view=details&search_constraint=terms&depth=0&query=GO:0019722) |
| GO:0042417 | 0.018 | 11.18 | 0 | 2 | 16 | [dopamine metabolic process](http://www.godatabase.org/cgi-bin/amigo/go.cgi?view=details&search_constraint=terms&depth=0&query=GO:0042417) |
| GO:0060711 | 0.018 | 11.18 | 0 | 2 | 16 | [labyrinthine layer development](http://www.godatabase.org/cgi-bin/amigo/go.cgi?view=details&search_constraint=terms&depth=0&query=GO:0060711) |
| GO:0009653 | 0.019 | 2.05 | 10 | 17 | 793 | [anatomical structure morphogenesis](http://www.godatabase.org/cgi-bin/amigo/go.cgi?view=details&search_constraint=terms&depth=0&query=GO:0009653) |
| GO:0009165 | 0.019 | 3.58 | 2 | 5 | 119 | [nucleotide biosynthetic process](http://www.godatabase.org/cgi-bin/amigo/go.cgi?view=details&search_constraint=terms&depth=0&query=GO:0009165) |
| GO:0007155 | 0.019 | 2.86 | 3 | 7 | 212 | [cell adhesion](http://www.godatabase.org/cgi-bin/amigo/go.cgi?view=details&search_constraint=terms&depth=0&query=GO:0007155) |
| GO:0022610 | 0.019 | 2.86 | 3 | 7 | 212 | [biological adhesion](http://www.godatabase.org/cgi-bin/amigo/go.cgi?view=details&search_constraint=terms&depth=0&query=GO:0022610) |
| GO:0031326 | 0.019 | 2.10 | 9 | 15 | 667 | [regulation of cellular biosynthetic process](http://www.godatabase.org/cgi-bin/amigo/go.cgi?view=details&search_constraint=terms&depth=0&query=GO:0031326) |
| GO:0048678 | 0.019 | 5.82 | 1 | 3 | 44 | [response to axon injury](http://www.godatabase.org/cgi-bin/amigo/go.cgi?view=details&search_constraint=terms&depth=0&query=GO:0048678) |
| GO:0010035 | 0.02 | 2.64 | 3 | 8 | 264 | [response to inorganic substance](http://www.godatabase.org/cgi-bin/amigo/go.cgi?view=details&search_constraint=terms&depth=0&query=GO:0010035) |
| GO:0051384 | 0.02 | 3.52 | 2 | 5 | 121 | [response to glucocorticoid stimulus](http://www.godatabase.org/cgi-bin/amigo/go.cgi?view=details&search_constraint=terms&depth=0&query=GO:0051384) |
| GO:0003158 | 0.02 | 10.43 | 0 | 2 | 17 | [endothelium development](http://www.godatabase.org/cgi-bin/amigo/go.cgi?view=details&search_constraint=terms&depth=0&query=GO:0003158) |
| GO:0045446 | 0.02 | 10.43 | 0 | 2 | 17 | [endothelial cell differentiation](http://www.godatabase.org/cgi-bin/amigo/go.cgi?view=details&search_constraint=terms&depth=0&query=GO:0045446) |
| GO:0007187 | 0.021 | 5.68 | 1 | 3 | 45 | [G-protein signaling, coupled to cyclic nucleotide second messenger](http://www.godatabase.org/cgi-bin/amigo/go.cgi?view=details&search_constraint=terms&depth=0&query=GO:0007187) |
| GO:0001525 | 0.021 | 3.49 | 2 | 5 | 122 | [angiogenesis](http://www.godatabase.org/cgi-bin/amigo/go.cgi?view=details&search_constraint=terms&depth=0&query=GO:0001525) |
| GO:0034404 | 0.021 | 3.46 | 2 | 5 | 123 | [nucleobase, nucleoside and nucleotide biosynthetic process](http://www.godatabase.org/cgi-bin/amigo/go.cgi?view=details&search_constraint=terms&depth=0&query=GO:0034404) |
| GO:0034654 | 0.021 | 3.46 | 2 | 5 | 123 | [nucleobase, nucleoside, nucleotide and nucleic acid biosynthetic process](http://www.godatabase.org/cgi-bin/amigo/go.cgi?view=details&search_constraint=terms&depth=0&query=GO:0034654) |
| GO:0019933 | 0.022 | 5.54 | 1 | 3 | 46 | [cAMP-mediated signaling](http://www.godatabase.org/cgi-bin/amigo/go.cgi?view=details&search_constraint=terms&depth=0&query=GO:0019933) |
| GO:0006874 | 0.022 | 3.43 | 2 | 5 | 124 | [cellular calcium ion homeostasis](http://www.godatabase.org/cgi-bin/amigo/go.cgi?view=details&search_constraint=terms&depth=0&query=GO:0006874) |
| GO:0045667 | 0.023 | 9.78 | 0 | 2 | 18 | [regulation of osteoblast differentiation](http://www.godatabase.org/cgi-bin/amigo/go.cgi?view=details&search_constraint=terms&depth=0&query=GO:0045667) |
| GO:0055074 | 0.023 | 3.40 | 2 | 5 | 125 | [calcium ion homeostasis](http://www.godatabase.org/cgi-bin/amigo/go.cgi?view=details&search_constraint=terms&depth=0&query=GO:0055074) |
| GO:0030198 | 0.023 | 5.42 | 1 | 3 | 47 | [extracellular matrix organization](http://www.godatabase.org/cgi-bin/amigo/go.cgi?view=details&search_constraint=terms&depth=0&query=GO:0030198) |
| GO:0080090 | 0.024 | 1.96 | 12 | 18 | 880 | [regulation of primary metabolic process](http://www.godatabase.org/cgi-bin/amigo/go.cgi?view=details&search_constraint=terms&depth=0&query=GO:0080090) |
| GO:0006928 | 0.024 | 2.40 | 4 | 9 | 328 | [cellular component movement](http://www.godatabase.org/cgi-bin/amigo/go.cgi?view=details&search_constraint=terms&depth=0&query=GO:0006928) |
| GO:0009890 | 0.025 | 2.70 | 3 | 7 | 223 | [negative regulation of biosynthetic process](http://www.godatabase.org/cgi-bin/amigo/go.cgi?view=details&search_constraint=terms&depth=0&query=GO:0009890) |
| GO:0017144 | 0.025 | 9.20 | 0 | 2 | 19 | [drug metabolic process](http://www.godatabase.org/cgi-bin/amigo/go.cgi?view=details&search_constraint=terms&depth=0&query=GO:0017144) |
| GO:0043534 | 0.025 | 9.20 | 0 | 2 | 19 | [blood vessel endothelial cell migration](http://www.godatabase.org/cgi-bin/amigo/go.cgi?view=details&search_constraint=terms&depth=0&query=GO:0043534) |
| GO:0009889 | 0.025 | 2.02 | 9 | 15 | 689 | [regulation of biosynthetic process](http://www.godatabase.org/cgi-bin/amigo/go.cgi?view=details&search_constraint=terms&depth=0&query=GO:0009889) |
| GO:0042327 | 0.025 | 3.93 | 1 | 4 | 86 | [positive regulation of phosphorylation](http://www.godatabase.org/cgi-bin/amigo/go.cgi?view=details&search_constraint=terms&depth=0&query=GO:0042327) |
| GO:0048468 | 0.026 | 2.14 | 7 | 12 | 505 | [cell development](http://www.godatabase.org/cgi-bin/amigo/go.cgi?view=details&search_constraint=terms&depth=0&query=GO:0048468) |
| GO:0003337 | 0.026 | 76.74 | 0 | 1 | 2 | [mesenchymal to epithelial transition involved in metanephros morphogenesis](http://www.godatabase.org/cgi-bin/amigo/go.cgi?view=details&search_constraint=terms&depth=0&query=GO:0003337) |
| GO:0007158 | 0.026 | 76.74 | 0 | 1 | 2 | [neuron cell-cell adhesion](http://www.godatabase.org/cgi-bin/amigo/go.cgi?view=details&search_constraint=terms&depth=0&query=GO:0007158) |
| GO:0010543 | 0.026 | 76.74 | 0 | 1 | 2 | [regulation of platelet activation](http://www.godatabase.org/cgi-bin/amigo/go.cgi?view=details&search_constraint=terms&depth=0&query=GO:0010543) |
| GO:0010544 | 0.026 | 76.74 | 0 | 1 | 2 | [negative regulation of platelet activation](http://www.godatabase.org/cgi-bin/amigo/go.cgi?view=details&search_constraint=terms&depth=0&query=GO:0010544) |
| GO:0010715 | 0.026 | 76.74 | 0 | 1 | 2 | [regulation of extracellular matrix disassembly](http://www.godatabase.org/cgi-bin/amigo/go.cgi?view=details&search_constraint=terms&depth=0&query=GO:0010715) |
| GO:0010771 | 0.026 | 76.74 | 0 | 1 | 2 | [negative regulation of cell morphogenesis involved in differentiation](http://www.godatabase.org/cgi-bin/amigo/go.cgi?view=details&search_constraint=terms&depth=0&query=GO:0010771) |
| GO:0021542 | 0.026 | 76.74 | 0 | 1 | 2 | [dentate gyrus development](http://www.godatabase.org/cgi-bin/amigo/go.cgi?view=details&search_constraint=terms&depth=0&query=GO:0021542) |
| GO:0032232 | 0.026 | 76.74 | 0 | 1 | 2 | [negative regulation of actin filament bundle assembly](http://www.godatabase.org/cgi-bin/amigo/go.cgi?view=details&search_constraint=terms&depth=0&query=GO:0032232) |
| GO:0032604 | 0.026 | 76.74 | 0 | 1 | 2 | [granulocyte macrophage colony-stimulating factor production](http://www.godatabase.org/cgi-bin/amigo/go.cgi?view=details&search_constraint=terms&depth=0&query=GO:0032604) |
| GO:0032645 | 0.026 | 76.74 | 0 | 1 | 2 | [regulation of granulocyte macrophage colony-stimulating factor production](http://www.godatabase.org/cgi-bin/amigo/go.cgi?view=details&search_constraint=terms&depth=0&query=GO:0032645) |
| GO:0032672 | 0.026 | 76.74 | 0 | 1 | 2 | [regulation of interleukin-3 production](http://www.godatabase.org/cgi-bin/amigo/go.cgi?view=details&search_constraint=terms&depth=0&query=GO:0032672) |
| GO:0032802 | 0.026 | 76.74 | 0 | 1 | 2 | [low-density lipoprotein receptor catabolic process](http://www.godatabase.org/cgi-bin/amigo/go.cgi?view=details&search_constraint=terms&depth=0&query=GO:0032802) |
| GO:0032803 | 0.026 | 76.74 | 0 | 1 | 2 | [regulation of low-density lipoprotein receptor catabolic process](http://www.godatabase.org/cgi-bin/amigo/go.cgi?view=details&search_constraint=terms&depth=0&query=GO:0032803) |
| GO:0042223 | 0.026 | 76.74 | 0 | 1 | 2 | [interleukin-3 biosynthetic process](http://www.godatabase.org/cgi-bin/amigo/go.cgi?view=details&search_constraint=terms&depth=0&query=GO:0042223) |
| GO:0042253 | 0.026 | 76.74 | 0 | 1 | 2 | [granulocyte macrophage colony-stimulating factor biosynthetic process](http://www.godatabase.org/cgi-bin/amigo/go.cgi?view=details&search_constraint=terms&depth=0&query=GO:0042253) |
| GO:0045399 | 0.026 | 76.74 | 0 | 1 | 2 | [regulation of interleukin-3 biosynthetic process](http://www.godatabase.org/cgi-bin/amigo/go.cgi?view=details&search_constraint=terms&depth=0&query=GO:0045399) |
| GO:0045401 | 0.026 | 76.74 | 0 | 1 | 2 | [positive regulation of interleukin-3 biosynthetic process](http://www.godatabase.org/cgi-bin/amigo/go.cgi?view=details&search_constraint=terms&depth=0&query=GO:0045401) |
| GO:0045423 | 0.026 | 76.74 | 0 | 1 | 2 | [regulation of granulocyte macrophage colony-stimulating factor biosynthetic process](http://www.godatabase.org/cgi-bin/amigo/go.cgi?view=details&search_constraint=terms&depth=0&query=GO:0045423) |
| GO:0045425 | 0.026 | 76.74 | 0 | 1 | 2 | [positive regulation of granulocyte macrophage colony-stimulating factor biosynthetic process](http://www.godatabase.org/cgi-bin/amigo/go.cgi?view=details&search_constraint=terms&depth=0&query=GO:0045425) |
| GO:0045541 | 0.026 | 76.74 | 0 | 1 | 2 | [negative regulation of cholesterol biosynthetic process](http://www.godatabase.org/cgi-bin/amigo/go.cgi?view=details&search_constraint=terms&depth=0&query=GO:0045541) |
| GO:0045835 | 0.026 | 76.74 | 0 | 1 | 2 | [negative regulation of meiosis](http://www.godatabase.org/cgi-bin/amigo/go.cgi?view=details&search_constraint=terms&depth=0&query=GO:0045835) |
| GO:0048341 | 0.026 | 76.74 | 0 | 1 | 2 | [paraxial mesoderm formation](http://www.godatabase.org/cgi-bin/amigo/go.cgi?view=details&search_constraint=terms&depth=0&query=GO:0048341) |
| GO:0048644 | 0.026 | 76.74 | 0 | 1 | 2 | [muscle organ morphogenesis](http://www.godatabase.org/cgi-bin/amigo/go.cgi?view=details&search_constraint=terms&depth=0&query=GO:0048644) |
| GO:0051451 | 0.026 | 76.74 | 0 | 1 | 2 | [myoblast migration](http://www.godatabase.org/cgi-bin/amigo/go.cgi?view=details&search_constraint=terms&depth=0&query=GO:0051451) |
| GO:0060598 | 0.026 | 76.74 | 0 | 1 | 2 | [dichotomous subdivision of terminal units involved in mammary gland duct morphogenesis](http://www.godatabase.org/cgi-bin/amigo/go.cgi?view=details&search_constraint=terms&depth=0&query=GO:0060598) |
| GO:0060708 | 0.026 | 76.74 | 0 | 1 | 2 | [spongiotrophoblast differentiation](http://www.godatabase.org/cgi-bin/amigo/go.cgi?view=details&search_constraint=terms&depth=0&query=GO:0060708) |
| GO:0060710 | 0.026 | 76.74 | 0 | 1 | 2 | [chorio-allantoic fusion](http://www.godatabase.org/cgi-bin/amigo/go.cgi?view=details&search_constraint=terms&depth=0&query=GO:0060710) |
| GO:0060836 | 0.026 | 76.74 | 0 | 1 | 2 | [lymphatic endothelial cell differentiation](http://www.godatabase.org/cgi-bin/amigo/go.cgi?view=details&search_constraint=terms&depth=0&query=GO:0060836) |
| GO:0061028 | 0.026 | 76.74 | 0 | 1 | 2 | [establishment of endothelial barrier](http://www.godatabase.org/cgi-bin/amigo/go.cgi?view=details&search_constraint=terms&depth=0&query=GO:0061028) |
| GO:0070373 | 0.026 | 76.74 | 0 | 1 | 2 | [negative regulation of ERK1 and ERK2 cascade](http://www.godatabase.org/cgi-bin/amigo/go.cgi?view=details&search_constraint=terms&depth=0&query=GO:0070373) |
| GO:0071107 | 0.026 | 76.74 | 0 | 1 | 2 | [response to parathyroid hormone stimulus](http://www.godatabase.org/cgi-bin/amigo/go.cgi?view=details&search_constraint=terms&depth=0&query=GO:0071107) |
| GO:0071277 | 0.026 | 76.74 | 0 | 1 | 2 | [cellular response to calcium ion](http://www.godatabase.org/cgi-bin/amigo/go.cgi?view=details&search_constraint=terms&depth=0&query=GO:0071277) |
| GO:0072009 | 0.026 | 76.74 | 0 | 1 | 2 | [nephron epithelium development](http://www.godatabase.org/cgi-bin/amigo/go.cgi?view=details&search_constraint=terms&depth=0&query=GO:0072009) |
| GO:0072073 | 0.026 | 76.74 | 0 | 1 | 2 | [kidney epithelium development](http://www.godatabase.org/cgi-bin/amigo/go.cgi?view=details&search_constraint=terms&depth=0&query=GO:0072073) |
| GO:0072077 | 0.026 | 76.74 | 0 | 1 | 2 | [renal vesicle morphogenesis](http://www.godatabase.org/cgi-bin/amigo/go.cgi?view=details&search_constraint=terms&depth=0&query=GO:0072077) |
| GO:0072087 | 0.026 | 76.74 | 0 | 1 | 2 | [renal vesicle development](http://www.godatabase.org/cgi-bin/amigo/go.cgi?view=details&search_constraint=terms&depth=0&query=GO:0072087) |
| GO:0072088 | 0.026 | 76.74 | 0 | 1 | 2 | [nephron epithelium morphogenesis](http://www.godatabase.org/cgi-bin/amigo/go.cgi?view=details&search_constraint=terms&depth=0&query=GO:0072088) |
| GO:0072210 | 0.026 | 76.74 | 0 | 1 | 2 | [metanephric nephron development](http://www.godatabase.org/cgi-bin/amigo/go.cgi?view=details&search_constraint=terms&depth=0&query=GO:0072210) |
| GO:0072215 | 0.026 | 76.74 | 0 | 1 | 2 | [regulation of metanephros development](http://www.godatabase.org/cgi-bin/amigo/go.cgi?view=details&search_constraint=terms&depth=0&query=GO:0072215) |
| GO:0072273 | 0.026 | 76.74 | 0 | 1 | 2 | [metanephric nephron morphogenesis](http://www.godatabase.org/cgi-bin/amigo/go.cgi?view=details&search_constraint=terms&depth=0&query=GO:0072273) |
| GO:0072283 | 0.026 | 76.74 | 0 | 1 | 2 | [metanephric renal vesicle morphogenesis](http://www.godatabase.org/cgi-bin/amigo/go.cgi?view=details&search_constraint=terms&depth=0&query=GO:0072283) |
| GO:0090022 | 0.026 | 76.74 | 0 | 1 | 2 | [regulation of neutrophil chemotaxis](http://www.godatabase.org/cgi-bin/amigo/go.cgi?view=details&search_constraint=terms&depth=0&query=GO:0090022) |
| GO:0090023 | 0.026 | 76.74 | 0 | 1 | 2 | [positive regulation of neutrophil chemotaxis](http://www.godatabase.org/cgi-bin/amigo/go.cgi?view=details&search_constraint=terms&depth=0&query=GO:0090023) |
| GO:0090136 | 0.026 | 76.74 | 0 | 1 | 2 | [epithelial cell-cell adhesion](http://www.godatabase.org/cgi-bin/amigo/go.cgi?view=details&search_constraint=terms&depth=0&query=GO:0090136) |
| GO:0090206 | 0.026 | 76.74 | 0 | 1 | 2 | [negative regulation of cholesterol metabolic process](http://www.godatabase.org/cgi-bin/amigo/go.cgi?view=details&search_constraint=terms&depth=0&query=GO:0090206) |
| GO:0045471 | 0.026 | 3.88 | 1 | 4 | 87 | [response to ethanol](http://www.godatabase.org/cgi-bin/amigo/go.cgi?view=details&search_constraint=terms&depth=0&query=GO:0045471) |
| GO:0010562 | 0.027 | 3.83 | 1 | 4 | 88 | [positive regulation of phosphorus metabolic process](http://www.godatabase.org/cgi-bin/amigo/go.cgi?view=details&search_constraint=terms&depth=0&query=GO:0010562) |
| GO:0045937 | 0.027 | 3.83 | 1 | 4 | 88 | [positive regulation of phosphate metabolic process](http://www.godatabase.org/cgi-bin/amigo/go.cgi?view=details&search_constraint=terms&depth=0&query=GO:0045937) |
| GO:0060429 | 0.027 | 2.64 | 3 | 7 | 228 | [epithelium development](http://www.godatabase.org/cgi-bin/amigo/go.cgi?view=details&search_constraint=terms&depth=0&query=GO:0060429) |
| GO:0002064 | 0.028 | 8.69 | 0 | 2 | 20 | [epithelial cell development](http://www.godatabase.org/cgi-bin/amigo/go.cgi?view=details&search_constraint=terms&depth=0&query=GO:0002064) |
| GO:0007566 | 0.028 | 8.69 | 0 | 2 | 20 | [embryo implantation](http://www.godatabase.org/cgi-bin/amigo/go.cgi?view=details&search_constraint=terms&depth=0&query=GO:0007566) |
| GO:0000902 | 0.028 | 2.62 | 3 | 7 | 229 | [cell morphogenesis](http://www.godatabase.org/cgi-bin/amigo/go.cgi?view=details&search_constraint=terms&depth=0&query=GO:0000902) |
| GO:0003001 | 0.028 | 3.20 | 2 | 5 | 132 | [generation of a signal involved in cell-cell signaling](http://www.godatabase.org/cgi-bin/amigo/go.cgi?view=details&search_constraint=terms&depth=0&query=GO:0003001) |
| GO:0023061 | 0.028 | 3.20 | 2 | 5 | 132 | [signal release](http://www.godatabase.org/cgi-bin/amigo/go.cgi?view=details&search_constraint=terms&depth=0&query=GO:0023061) |
| GO:0031960 | 0.028 | 3.20 | 2 | 5 | 132 | [response to corticosteroid stimulus](http://www.godatabase.org/cgi-bin/amigo/go.cgi?view=details&search_constraint=terms&depth=0&query=GO:0031960) |
| GO:0006875 | 0.03 | 3.15 | 2 | 5 | 134 | [cellular metal ion homeostasis](http://www.godatabase.org/cgi-bin/amigo/go.cgi?view=details&search_constraint=terms&depth=0&query=GO:0006875) |
| GO:0007186 | 0.031 | 2.79 | 2 | 6 | 183 | [G-protein coupled receptor protein signaling pathway](http://www.godatabase.org/cgi-bin/amigo/go.cgi?view=details&search_constraint=terms&depth=0&query=GO:0007186) |
| GO:0051591 | 0.032 | 4.76 | 1 | 3 | 53 | [response to cAMP](http://www.godatabase.org/cgi-bin/amigo/go.cgi?view=details&search_constraint=terms&depth=0&query=GO:0051591) |
| GO:0055065 | 0.032 | 3.08 | 2 | 5 | 137 | [metal ion homeostasis](http://www.godatabase.org/cgi-bin/amigo/go.cgi?view=details&search_constraint=terms&depth=0&query=GO:0055065) |
| GO:0007200 | 0.033 | 7.81 | 0 | 2 | 22 | [activation of phospholipase C activity by G-protein coupled receptor protein signaling pathway coupled to IP3 second messenger](http://www.godatabase.org/cgi-bin/amigo/go.cgi?view=details&search_constraint=terms&depth=0&query=GO:0007200) |
| GO:0048589 | 0.033 | 3.05 | 2 | 5 | 138 | [developmental growth](http://www.godatabase.org/cgi-bin/amigo/go.cgi?view=details&search_constraint=terms&depth=0&query=GO:0048589) |
| GO:0009892 | 0.033 | 2.25 | 5 | 9 | 347 | [negative regulation of metabolic process](http://www.godatabase.org/cgi-bin/amigo/go.cgi?view=details&search_constraint=terms&depth=0&query=GO:0009892) |
| GO:0051171 | 0.034 | 1.96 | 9 | 14 | 649 | [regulation of nitrogen compound metabolic process](http://www.godatabase.org/cgi-bin/amigo/go.cgi?view=details&search_constraint=terms&depth=0&query=GO:0051171) |
| GO:0050793 | 0.034 | 2.16 | 5 | 10 | 405 | [regulation of developmental process](http://www.godatabase.org/cgi-bin/amigo/go.cgi?view=details&search_constraint=terms&depth=0&query=GO:0050793) |
| GO:0042221 | 0.034 | 1.84 | 14 | 20 | 1049 | [response to chemical stimulus](http://www.godatabase.org/cgi-bin/amigo/go.cgi?view=details&search_constraint=terms&depth=0&query=GO:0042221) |
| GO:0008284 | 0.036 | 2.48 | 3 | 7 | 241 | [positive regulation of cell proliferation](http://www.godatabase.org/cgi-bin/amigo/go.cgi?view=details&search_constraint=terms&depth=0&query=GO:0008284) |
| GO:0033280 | 0.036 | 7.44 | 0 | 2 | 23 | [response to vitamin D](http://www.godatabase.org/cgi-bin/amigo/go.cgi?view=details&search_constraint=terms&depth=0&query=GO:0033280) |
| GO:0045934 | 0.037 | 2.66 | 3 | 6 | 191 | [negative regulation of nucleobase, nucleoside, nucleotide and nucleic acid metabolic process](http://www.godatabase.org/cgi-bin/amigo/go.cgi?view=details&search_constraint=terms&depth=0&query=GO:0045934) |
| GO:0010557 | 0.038 | 2.31 | 4 | 8 | 298 | [positive regulation of macromolecule biosynthetic process](http://www.godatabase.org/cgi-bin/amigo/go.cgi?view=details&search_constraint=terms&depth=0&query=GO:0010557) |
| GO:0023034 | 0.038 | 2.05 | 6 | 11 | 473 | [intracellular signaling pathway](http://www.godatabase.org/cgi-bin/amigo/go.cgi?view=details&search_constraint=terms&depth=0&query=GO:0023034) |
| GO:0003008 | 0.038 | 2.00 | 7 | 12 | 534 | [system process](http://www.godatabase.org/cgi-bin/amigo/go.cgi?view=details&search_constraint=terms&depth=0&query=GO:0003008) |
| GO:0010604 | 0.039 | 2.11 | 5 | 10 | 414 | [positive regulation of macromolecule metabolic process](http://www.godatabase.org/cgi-bin/amigo/go.cgi?view=details&search_constraint=terms&depth=0&query=GO:0010604) |
| GO:0006584 | 0.039 | 7.10 | 0 | 2 | 24 | [catecholamine metabolic process](http://www.godatabase.org/cgi-bin/amigo/go.cgi?view=details&search_constraint=terms&depth=0&query=GO:0006584) |
| GO:0009712 | 0.039 | 7.10 | 0 | 2 | 24 | [catechol metabolic process](http://www.godatabase.org/cgi-bin/amigo/go.cgi?view=details&search_constraint=terms&depth=0&query=GO:0009712) |
| GO:0014812 | 0.039 | 7.10 | 0 | 2 | 24 | [muscle cell migration](http://www.godatabase.org/cgi-bin/amigo/go.cgi?view=details&search_constraint=terms&depth=0&query=GO:0014812) |
| GO:0018958 | 0.039 | 7.10 | 0 | 2 | 24 | [phenol metabolic process](http://www.godatabase.org/cgi-bin/amigo/go.cgi?view=details&search_constraint=terms&depth=0&query=GO:0018958) |
| GO:0034311 | 0.039 | 7.10 | 0 | 2 | 24 | [diol metabolic process](http://www.godatabase.org/cgi-bin/amigo/go.cgi?view=details&search_constraint=terms&depth=0&query=GO:0034311) |
| GO:0050921 | 0.039 | 7.10 | 0 | 2 | 24 | [positive regulation of chemotaxis](http://www.godatabase.org/cgi-bin/amigo/go.cgi?view=details&search_constraint=terms&depth=0&query=GO:0050921) |
| GO:0060349 | 0.039 | 7.10 | 0 | 2 | 24 | [bone morphogenesis](http://www.godatabase.org/cgi-bin/amigo/go.cgi?view=details&search_constraint=terms&depth=0&query=GO:0060349) |
| GO:0001810 | 0.039 | 38.36 | 0 | 1 | 3 | [regulation of type I hypersensitivity](http://www.godatabase.org/cgi-bin/amigo/go.cgi?view=details&search_constraint=terms&depth=0&query=GO:0001810) |
| GO:0001812 | 0.039 | 38.36 | 0 | 1 | 3 | [positive regulation of type I hypersensitivity](http://www.godatabase.org/cgi-bin/amigo/go.cgi?view=details&search_constraint=terms&depth=0&query=GO:0001812) |
| GO:0002063 | 0.039 | 38.36 | 0 | 1 | 3 | [chondrocyte development](http://www.godatabase.org/cgi-bin/amigo/go.cgi?view=details&search_constraint=terms&depth=0&query=GO:0002063) |
| GO:0003338 | 0.039 | 38.36 | 0 | 1 | 3 | [metanephros morphogenesis](http://www.godatabase.org/cgi-bin/amigo/go.cgi?view=details&search_constraint=terms&depth=0&query=GO:0003338) |
| GO:0006378 | 0.039 | 38.36 | 0 | 1 | 3 | [mRNA polyadenylation](http://www.godatabase.org/cgi-bin/amigo/go.cgi?view=details&search_constraint=terms&depth=0&query=GO:0006378) |
| GO:0007208 | 0.039 | 38.36 | 0 | 1 | 3 | [activation of phospholipase C activity by serotonin receptor signaling pathway](http://www.godatabase.org/cgi-bin/amigo/go.cgi?view=details&search_constraint=terms&depth=0&query=GO:0007208) |
| GO:0016068 | 0.039 | 38.36 | 0 | 1 | 3 | [type I hypersensitivity](http://www.godatabase.org/cgi-bin/amigo/go.cgi?view=details&search_constraint=terms&depth=0&query=GO:0016068) |
| GO:0022617 | 0.039 | 38.36 | 0 | 1 | 3 | [extracellular matrix disassembly](http://www.godatabase.org/cgi-bin/amigo/go.cgi?view=details&search_constraint=terms&depth=0&query=GO:0022617) |
| GO:0032488 | 0.039 | 38.36 | 0 | 1 | 3 | [Cdc42 protein signal transduction](http://www.godatabase.org/cgi-bin/amigo/go.cgi?view=details&search_constraint=terms&depth=0&query=GO:0032488) |
| GO:0033631 | 0.039 | 38.36 | 0 | 1 | 3 | [cell-cell adhesion mediated by integrin](http://www.godatabase.org/cgi-bin/amigo/go.cgi?view=details&search_constraint=terms&depth=0&query=GO:0033631) |
| GO:0034382 | 0.039 | 38.36 | 0 | 1 | 3 | [chylomicron remnant clearance](http://www.godatabase.org/cgi-bin/amigo/go.cgi?view=details&search_constraint=terms&depth=0&query=GO:0034382) |
| GO:0040019 | 0.039 | 38.36 | 0 | 1 | 3 | [positive regulation of embryonic development](http://www.godatabase.org/cgi-bin/amigo/go.cgi?view=details&search_constraint=terms&depth=0&query=GO:0040019) |
| GO:0042438 | 0.039 | 38.36 | 0 | 1 | 3 | [melanin biosynthetic process](http://www.godatabase.org/cgi-bin/amigo/go.cgi?view=details&search_constraint=terms&depth=0&query=GO:0042438) |
| GO:0042695 | 0.039 | 38.36 | 0 | 1 | 3 | [thelarche](http://www.godatabase.org/cgi-bin/amigo/go.cgi?view=details&search_constraint=terms&depth=0&query=GO:0042695) |
| GO:0045668 | 0.039 | 38.36 | 0 | 1 | 3 | [negative regulation of osteoblast differentiation](http://www.godatabase.org/cgi-bin/amigo/go.cgi?view=details&search_constraint=terms&depth=0&query=GO:0045668) |
| GO:0051000 | 0.039 | 38.36 | 0 | 1 | 3 | [positive regulation of nitric-oxide synthase activity](http://www.godatabase.org/cgi-bin/amigo/go.cgi?view=details&search_constraint=terms&depth=0&query=GO:0051000) |
| GO:0051005 | 0.039 | 38.36 | 0 | 1 | 3 | [negative regulation of lipoprotein lipase activity](http://www.godatabase.org/cgi-bin/amigo/go.cgi?view=details&search_constraint=terms&depth=0&query=GO:0051005) |
| GO:0051044 | 0.039 | 38.36 | 0 | 1 | 3 | [positive regulation of membrane protein ectodomain proteolysis](http://www.godatabase.org/cgi-bin/amigo/go.cgi?view=details&search_constraint=terms&depth=0&query=GO:0051044) |
| GO:0060231 | 0.039 | 38.36 | 0 | 1 | 3 | [mesenchymal to epithelial transition](http://www.godatabase.org/cgi-bin/amigo/go.cgi?view=details&search_constraint=terms&depth=0&query=GO:0060231) |
| GO:0060426 | 0.039 | 38.36 | 0 | 1 | 3 | [lung vasculature development](http://www.godatabase.org/cgi-bin/amigo/go.cgi?view=details&search_constraint=terms&depth=0&query=GO:0060426) |
| GO:0060600 | 0.039 | 38.36 | 0 | 1 | 3 | [dichotomous subdivision of an epithelial terminal unit](http://www.godatabase.org/cgi-bin/amigo/go.cgi?view=details&search_constraint=terms&depth=0&query=GO:0060600) |
| GO:0060744 | 0.039 | 38.36 | 0 | 1 | 3 | [mammary gland branching involved in thelarche](http://www.godatabase.org/cgi-bin/amigo/go.cgi?view=details&search_constraint=terms&depth=0&query=GO:0060744) |
| GO:0060750 | 0.039 | 38.36 | 0 | 1 | 3 | [epithelial cell proliferation involved in mammary gland duct elongation](http://www.godatabase.org/cgi-bin/amigo/go.cgi?view=details&search_constraint=terms&depth=0&query=GO:0060750) |
| GO:0070669 | 0.039 | 38.36 | 0 | 1 | 3 | [response to interleukin-2](http://www.godatabase.org/cgi-bin/amigo/go.cgi?view=details&search_constraint=terms&depth=0&query=GO:0070669) |
| GO:0072028 | 0.039 | 38.36 | 0 | 1 | 3 | [nephron morphogenesis](http://www.godatabase.org/cgi-bin/amigo/go.cgi?view=details&search_constraint=terms&depth=0&query=GO:0072028) |
| GO:0050789 | 0.039 | 1.86 | 25 | 31 | 1887 | [regulation of biological process](http://www.godatabase.org/cgi-bin/amigo/go.cgi?view=details&search_constraint=terms&depth=0&query=GO:0050789) |
| GO:0050880 | 0.04 | 4.32 | 1 | 3 | 58 | [regulation of blood vessel size](http://www.godatabase.org/cgi-bin/amigo/go.cgi?view=details&search_constraint=terms&depth=0&query=GO:0050880) |
| GO:0006753 | 0.041 | 2.59 | 3 | 6 | 196 | [nucleoside phosphate metabolic process](http://www.godatabase.org/cgi-bin/amigo/go.cgi?view=details&search_constraint=terms&depth=0&query=GO:0006753) |
| GO:0009117 | 0.041 | 2.59 | 3 | 6 | 196 | [nucleotide metabolic process](http://www.godatabase.org/cgi-bin/amigo/go.cgi?view=details&search_constraint=terms&depth=0&query=GO:0009117) |
| GO:0043009 | 0.041 | 2.59 | 3 | 6 | 196 | [chordate embryonic development](http://www.godatabase.org/cgi-bin/amigo/go.cgi?view=details&search_constraint=terms&depth=0&query=GO:0043009) |
| GO:0060173 | 0.041 | 4.24 | 1 | 3 | 59 | [limb development](http://www.godatabase.org/cgi-bin/amigo/go.cgi?view=details&search_constraint=terms&depth=0&query=GO:0060173) |
| GO:0007417 | 0.042 | 2.39 | 3 | 7 | 249 | [central nervous system development](http://www.godatabase.org/cgi-bin/amigo/go.cgi?view=details&search_constraint=terms&depth=0&query=GO:0007417) |
| GO:0060348 | 0.042 | 6.79 | 0 | 2 | 25 | [bone development](http://www.godatabase.org/cgi-bin/amigo/go.cgi?view=details&search_constraint=terms&depth=0&query=GO:0060348) |
| GO:0007568 | 0.043 | 2.56 | 3 | 6 | 198 | [aging](http://www.godatabase.org/cgi-bin/amigo/go.cgi?view=details&search_constraint=terms&depth=0&query=GO:0007568) |
| GO:0014070 | 0.043 | 2.56 | 3 | 6 | 198 | [response to organic cyclic substance](http://www.godatabase.org/cgi-bin/amigo/go.cgi?view=details&search_constraint=terms&depth=0&query=GO:0014070) |
| GO:0030005 | 0.043 | 2.83 | 2 | 5 | 148 | [cellular di-, tri-valent inorganic cation homeostasis](http://www.godatabase.org/cgi-bin/amigo/go.cgi?view=details&search_constraint=terms&depth=0&query=GO:0030005) |
| GO:0018108 | 0.043 | 4.16 | 1 | 3 | 60 | [peptidyl-tyrosine phosphorylation](http://www.godatabase.org/cgi-bin/amigo/go.cgi?view=details&search_constraint=terms&depth=0&query=GO:0018108) |
| GO:0018212 | 0.043 | 4.16 | 1 | 3 | 60 | [peptidyl-tyrosine modification](http://www.godatabase.org/cgi-bin/amigo/go.cgi?view=details&search_constraint=terms&depth=0&query=GO:0018212) |
| GO:0007202 | 0.045 | 6.50 | 0 | 2 | 26 | [activation of phospholipase C activity](http://www.godatabase.org/cgi-bin/amigo/go.cgi?view=details&search_constraint=terms&depth=0&query=GO:0007202) |
| GO:0010518 | 0.045 | 6.50 | 0 | 2 | 26 | [positive regulation of phospholipase activity](http://www.godatabase.org/cgi-bin/amigo/go.cgi?view=details&search_constraint=terms&depth=0&query=GO:0010518) |
| GO:0010863 | 0.045 | 6.50 | 0 | 2 | 26 | [positive regulation of phospholipase C activity](http://www.godatabase.org/cgi-bin/amigo/go.cgi?view=details&search_constraint=terms&depth=0&query=GO:0010863) |
| GO:0035150 | 0.045 | 4.09 | 1 | 3 | 61 | [regulation of tube size](http://www.godatabase.org/cgi-bin/amigo/go.cgi?view=details&search_constraint=terms&depth=0&query=GO:0035150) |
| GO:0061138 | 0.045 | 4.09 | 1 | 3 | 61 | [morphogenesis of a branching epithelium](http://www.godatabase.org/cgi-bin/amigo/go.cgi?view=details&search_constraint=terms&depth=0&query=GO:0061138) |
| GO:0031324 | 0.046 | 2.21 | 4 | 8 | 310 | [negative regulation of cellular metabolic process](http://www.godatabase.org/cgi-bin/amigo/go.cgi?view=details&search_constraint=terms&depth=0&query=GO:0031324) |
| GO:0055066 | 0.047 | 2.75 | 2 | 5 | 152 | [di-, tri-valent inorganic cation homeostasis](http://www.godatabase.org/cgi-bin/amigo/go.cgi?view=details&search_constraint=terms&depth=0&query=GO:0055066) |
| GO:0010517 | 0.048 | 6.24 | 0 | 2 | 27 | [regulation of phospholipase activity](http://www.godatabase.org/cgi-bin/amigo/go.cgi?view=details&search_constraint=terms&depth=0&query=GO:0010517) |
| GO:0050920 | 0.048 | 6.24 | 0 | 2 | 27 | [regulation of chemotaxis](http://www.godatabase.org/cgi-bin/amigo/go.cgi?view=details&search_constraint=terms&depth=0&query=GO:0050920) |
