# Supplementary material for: Sex-Related Differences in Gene Expression by Porcine Aortic Valvular Interstitial Cells
Source: PLoS One. 2012 Jul 10;7(7):e39980. doi: 10.1371/journal.pone.0039980 (PMC3393722; doi:10.1371/journal.pone.0039980)
Supplement: Table S4 — Summary of significant biological processes determined by GO enrichment analysis, categorized according to disease-related pathway grouping. Expected count (Exp Count) refers to the number of differentially expressed genes that are predicted within the GO term tested, Count refers to the number of differentially expressed genes that were found within the GO term tested, and Size refers to the number of genes in the microarray that are listed within the GO term tested. (DOC) [file pone.0039980.s008.doc]

**Table S4**. Summary of significant biological processes determined by GO enrichment analysis, categorized according to disease-related pathway grouping. Expected count (Exp Count) refers to the number of differentially expressed genes that are predicted within the GO term tested, Count refers to the number of differentially expressed genes that were found within the GO term tested, and Size refers to the number of genes in the microarray that are listed within the GO term tested.

| **GO BP ID** | **Pathway Description** | **P value** | **Odds ratio** | **Exp Count** | **Count** | **Size** |
| --- | --- | --- | --- | --- | --- | --- |
| *Calcification* | | | | | | |
| GO:0060349 | bone morphogenesis | 0.039 | 7.10 | 0 | 2 | 24 |
| GO:0001649 | osteoblast differentiation | 0.012 | 7.03 | 0 | 3 | 37 |
| GO:0045668 | negative regulation of osteoblast differentiation | 0.039 | 38.36 | 0 | 1 | 3 |
| GO:0001503 | ossification | 0.001 | 5.65 | 1 | 7 | 114 |
| GO:0060348 | bone development | 0.042 | 6.79 | 0 | 2 | 25 |
| GO:0030278 | regulation of ossification | 0.003 | 7.76 | 1 | 4 | 46 |
| GO:0003416 | endochondral bone growth | 0.001 | 157.14 | 0 | 2 | 3 |
| GO:0060351 | cartilage development involved in endochondral bone morphogenesis | 0.002 | 39.25 | 0 | 2 | 6 |
| GO:0060350 | endochondral bone morphogenesis | 0.012 | 14.24 | 0 | 2 | 13 |
| GO:0045667 | regulation of osteoblast differentiation | 0.023 | 9.78 | 0 | 2 | 18 |
| GO:0071107 | response to parathyroid hormone stimulus | 0.026 | 76.74 | 0 | 1 | 2 |
|  | | | | | | |
| *Cell proliferation* | | | | | | |
| GO:0042127 | regulation of cell proliferation | 0.004 | 2.87 | 5 | 12 | 393 |
| GO:0008283 | cell proliferation | 0.008 | 2.50 | 6 | 13 | 488 |
| GO:0008284 | positive regulation of cell proliferation | 0.036 | 2.48 | 3 | 7 | 241 |
| GO:0008285 | negative regulation of cell proliferation | 0.012 | 14.24 | 0 | 2 | 13 |
| GO:0033002 | muscle cell proliferation | 0 | 7.51 | 1 | 6 | 74 |
| GO:0048659 | smooth muscle cell proliferation | 0.001 | 8.01 | 1 | 5 | 57 |
| GO:0048660 | regulation of smooth muscle cell proliferation | 0.001 | 8.01 | 1 | 5 | 57 |
|  | | | | | | |
| *Angiogenesis* | | | | | | |
| GO:0001944 | vasculature development | 0.003 | 3.72 | 3 | 8 | 194 |
| GO:0001568 | blood vessel development | 0.01 | 3.30 | 2 | 7 | 186 |
| GO:0048514 | blood vessel morphogenesis | 0.004 | 3.89 | 2 | 7 | 160 |
| GO:0001525 | angiogenesis | 0.021 | 3.49 | 2 | 5 | 122 |
| GO:0048844 | artery morphogenesis | 0.01 | 15.67 | 0 | 2 | 12 |
| GO:0060840 | artery development | 0.012 | 14.24 | 0 | 2 | 13 |
| GO:0016525 | negative regulation of angiogenesis | 0.012 | 14.24 | 0 | 2 | 13 |
|  | | | | | | |
| *ECM remodeling* | | | | | | |
| GO:0010716 | negative regulation of extracellular matrix disassembly | 0.013 | Inf | 0 | 1 | 1 |
| GO:0030198 | extracellular matrix organization | 0.023 | 5.42 | 1 | 3 | 47 |
| GO:0022617 | extracellular matrix disassembly | 0.039 | 38.36 | 0 | 1 | 3 |
|  | | | | | | |
| *Adhesion* | | | | | | |
| GO:0016337 | cell-cell adhesion | 0.008 | 4.57 | 1 | 5 | 95 |
| GO:0033632 | regulation of cell-cell adhesion mediated by integrin | 0.013 | Inf | 0 | 1 | 1 |
| GO:0007155 | cell adhesion | 0.019 | 2.86 | 3 | 7 | 212 |
| GO:0022610 | biological adhesion | 0.019 | 2.86 | 3 | 7 | 212 |
|  | | | | | | |
| *Migration* | | | | | | |
| GO:0030336 | negative regulation of cell migration | 0 | 15.62 | 0 | 4 | 25 |
| GO:0040012 | regulation of locomotion | 0.002 | 4.69 | 2 | 7 | 135 |
| GO:0030334 | regulation of cell migration | 0.004 | 4.62 | 2 | 6 | 115 |
| GO:0040011 | locomotion | 0.007 | 2.72 | 5 | 11 | 372 |
| GO:0016477 | cell migration | 0.009 | 2.90 | 4 | 9 | 278 |
| GO:0048870 | cell motility | 0.01 | 2.83 | 4 | 9 | 284 |
| GO:0051451 | myoblast migration | 0.026 | 76.74 | 0 | 1 | 2 |
| GO:0014812 | muscle cell migration | 0.039 | 7.10 | 0 | 2 | 24 |
| GO:0050921 | positive regulation of chemotaxis | 0.039 | 7.10 | 0 | 2 | 24 |
| GO:0050920 | regulation of chemotaxis | 0.048 | 6.24 | 0 | 2 | 27 |
|  | | | | | | |
| *Inflammation* | | | | | | |
| GO:0071105 | response to interleukin-11 | 0.013 | Inf | 0 | 1 | 1 |
| GO:0032604 | granulocyte macrophage colony-stimulating factor production | 0.026 | 76.74 | 0 | 1 | 2 |
| GO:0032645 | regulation of granulocyte macrophage colony-stimulating factor production | 0.026 | 76.74 | 0 | 1 | 2 |
| GO:0032672 | regulation of interleukin-3 production | 0.026 | 76.74 | 0 | 1 | 2 |
| GO:0042223 | interleukin-3 biosynthetic process | 0.026 | 76.74 | 0 | 1 | 2 |
| GO:0042253 | granulocyte macrophage colony-stimulating factor biosynthetic process | 0.026 | 76.74 | 0 | 1 | 2 |
| GO:0045399 | regulation of interleukin-3 biosynthetic process | 0.026 | 76.74 | 0 | 1 | 2 |
| GO:0045401 | positive regulation of interleukin-3 biosynthetic process | 0.026 | 76.74 | 0 | 1 | 2 |
| GO:0045423 | regulation of granulocyte macrophage colony-stimulating factor biosynthetic process | 0.026 | 76.74 | 0 | 1 | 2 |
| GO:0045425 | positive regulation of granulocyte macrophage colony-stimulating factor biosynthetic process | 0.026 | 76.74 | 0 | 1 | 2 |
