# Supplementary material for: Sex-Related Differences in Gene Expression by Porcine Aortic Valvular Interstitial Cells
Source: PLoS One. 2012 Jul 10;7(7):e39980. doi: 10.1371/journal.pone.0039980 (PMC3393722; doi:10.1371/journal.pone.0039980)
Supplement: Data Supplement Text S1 — Expanded description of Materials and Methods. (DOC) [file pone.0039980.s009.doc]

**SUPPLEMENTAL MATERIAL**

|  |  |
| --- | --- |

**METHODS**

All chemicals were obtained from Sigma-Aldrich, St. Louis, MO unless otherwise noted.

**RNA isolation**

Aortic valve leaflets were surgically removed using sterile techniques from hearts obtained from 5 month old female and castrated male pigs (Hoesly’s Meats, New Glarus, WI) weighing approximately 90 kg, and the endothelial cells were manually denuded with a scalpel. The leaflets were then rinsed twice in basal Medium 199 solution supplemented with 200 U/ml penicillin and 200 μg/ml streptomycin and stored in RNAlater until processing. The leaflets were then homogenized using sterile stainless steel beads and a BulletBlender tube agitator (Next Advance, Averill Park, NY) for 5 minutes following manufacturer’s instructions. RNA was isolated from the tissue using the RNeasy fibrous tissue spin-column kit (Qiagen, Valencia, CA) according to the manufacturer’s instructions. Briefly, the homogenized sample was incubated with proteinase K, combined with 100% ethanol, and the supernatant was added to the spin column system. The RNA bound to the column was washed and treated with DNAse I included in the kit to remove any genomic DNA contamination. Final RNA was eluted into RNAse-free water.

Successful endothelial denudation of leaflets was confirmed on a parallel set of samples via immunohistochemical detection of von Willebrand factor (vWf) using standard techniques. Briefly, leaflets were fixed in 10% buffered formalin, embedded in tissue freezing medium, cryosectioned into 7 μm slices, then washed and blocked with 5% bovine serum albumin (BSA) in PBS prior to overnight incubation with anti-vWf (rabbit polyclonal, 8 μg/ml, Abcam, Cambridge, MA) diluted in 1% BSA in PBS at 4°C. Goat anti-rabbit AlexaFluor 488 (Invitrogen, 2 μg/mL) was applied for 1 hour at room temperature and counterstaining with DAPI (20 μg/mL). Fluorescence images were captured of each section using an Olympus IX51 fluorescence microscope (Olympus, Center Valley, PA).

**Microarray hybridization**

RNA samples from three male and three female pigs were selected for microarray hybridization analysis based on sample purity and quality control analysis. RNA sample integrity was determined by the identification of 28S and 18S bands of ribosomal RNA on agarose gel electrophoresis. Affymetrix GeneChip Porcine Genome Arrays (Affymetrix, Santa Clara, CA) containing 23,937 probesets that interrogate 23,256 transcripts from 20,201 *S. scrofa* genes were selected for use in this study. The samples were labeled, and the arrays were processed at the University of Wisconsin-Madison Gene Expression Center (Madison, WI) following all appropriate manufacturer’s instructions. Briefly, 400 ng of total RNA was labeled using the Ambion MessageAmp™ Premier IVT kit (Ambion, Austin, TX). An 8 hour IVT was carried out at 40°C, followed by fragmentation of the aRNA. One microgram of the fragmented and unfragmented aRNA was run on a 2% agarose gel to verify the product sizes. A hybridization cocktail was prepared according to protocols and procedures in the AFX Expression Analysis Technical Manual (P/N 702232 Rev.3) with specific protocols for using the GeneChip Hybridization, Wash, and Stain kit (Affymetrix). Ten micrograms of aRNA was applied to each AFX Porcine array. The arrays were hybridized for 16 hours at 45°C in an AFX Hybridization Oven 640. Post processing of the Porcine GeneChips was performed on the AFX 450 Fluidics Station according to all AFX protocols and procedures defined for the porcine array and scanned on a GC3000 G7 scanner. Data were extracted from images using the AFX Expression Console v1.1 software.

**Microarray data analysis**

The microarray data were processed using the open source statistical language R v2.12.0 and the libraries included in the Bioconductor Project [^1^](#_ENREF_11). The *affy* and *simpleaffy* packages [^2^](#_ENREF_12) from the Bioconductor project were used to assess the quality of RNA sample and hybridization. Raw expression values from *.CEL files were background-corrected and normalized using the Robust Multi-array Analysis (RMA) method [^3^](#_ENREF_13), and filtered based on the coefficient of variance (standard deviation divided by the mean) to exclude gene expression values that did not have sufficient similarity across the biological replicates. The mean expression of each probe set in the male samples was then compared with that of the female samples. The differential gene expression between male and female samples was determined and a significant genes list was generated using the Empirical Bayes *t*-test statistic from the *limma* package [^4^](#_ENREF_14), where the significance threshold was set to a false discovery rate (FDR) of 0.05 and a minimum fold change of 2. Gene ontology (GO) enrichment analysis was performed on the significant genes list using the GOstats package [^5^](#_ENREF_15). The significant genes list was annotated using the chip annotations provided by both Affymetrix (NetAffx) and Tsai et al.[^6^](#_ENREF_16). The data discussed in this publication have been deposited in NCBI's Gene Expression Omnibus and are accessible through GEO Series accession number GSE33654 (http://www.ncbi.nlm.nih.gov/geo/query/acc.cgi?acc=GSE33654).

Extended pathway analysis was performed on the significant genes list using Ingenuity Pathway Analysis (IPA) Software v9.0 and services (Ingenuity Systems, Redwood City, CA). The significant genes list was divided into genes upregulated in either male or female samples and functional analysis was performed on each list in IPA using default parameters to identify biological functions and/or diseases that were enriched most significantly different between males and females. A p value was calculated for each gene group mapped to certain functions using the Fisher’s exact right-tailed test to determine the probability that the association between the genes and each biological function was not explained by chance alone. In addition, significant genes from the dataset that met filter criteria and had at least one published piece of evidence linking it to one other molecule in the Ingenuity Knowledge Base were named focus genes to form a global molecular network. Other genes that were not from the dataset but were indispensable to the networks were supplemented based on their connectivity as defined by literature references. The IPA program computed a p-value-based score for each network according to the fit of the network to the set of composing genes. Networks were graphically depicted for visualization of gene relationships.

**Validation of microarray via qRT-PCR**

The differential expression of a group of genes identified by microarray analysis was validated by quantitative real-time quantitative polymerase chain reaction (qRT-PCR) using the same six animal samples used in the microarray. RNA was reversed transcribed using a High Capacity cDNA Reverse Transcription kit (Applied Biosystems, Carlsbad, CA). The reaction included 20 μL reverse transcription mixture containing: 2 μl 10x Buffer, 1 μl RNase inhibitor, 4 mM dNTPs, 1 μL random primers , 50 units of MultiScribe Reverse Transcriptase and 250 ng of RNA. Each sample was incubated for 10 minutes at 25ºC, then 120 minutes at 37ºC and finally 85ºC for 5 minutes. Samples were stored at 4ºC until further processing.

qRT-PCR amplification was performed using TaqMan Gene Expression Assays (Applied Biosystems). Each reaction consisted of 10 μL TaqMan master mix, 7 μL nuclease-free water, 1 μL gene-specific assay and 2 μL of sample cDNA. After initial 50ºC incubation for 2 minutes and 95ºC incubation for 10 minutes, the amplification cycles were 15 seconds at 95ºC and 1 minute at 60ºC. The cycle amplification was repeated for 40 cycles on a StepOnePlus real-time PCR thermocycler (Applied Biosystems). Target genes were normalized to GAPDH, and Ct values were analyzed using the comparative ΔΔCt method relative to female samples [^7^](#_ENREF_17). Statistical analysis of qRT-PCR data was performed using one-way analysis of variance (ANOVA) with Tukey’s HSD post-test. P-values less than or equal to 0.05 were considered statistically significant.

**In vitro culture and analysis of sex-separated VIC populations**

VICs were isolated from female and castrated male porcine aortic valves (Hoesly’s Meats, New Glarus, WI) within 5 hours of death using the collagenase digestion protocol described previously ^8^. Following enzyme treatment, the VICs were removed from the supernatant and suspended in growth medium (Medium 199, 15% fetal bovine serum (FBS)) supplemented with 2 mmol/L L-glutamine, 100 U/mL penicillin, and 100 μg/mL streptomycin at 37°C, 5% CO_2_ and cultured for 2 passages on tissue culture-treated polystyrene (TCPS) plates. Following trypsinization, passage 2 VICs were seeded on 24-well TCPS plates at a density of 50,000 cells/cm^2^ in Medium 199 supplemented with 1% charcoal/dextran treated FBS (Thermo Fisher Scientific, Waltham, MA), 100 U/mL penicillin, 100 ug/mL streptomycin and 2 mM L-glutamine and refed every 48 hours with serum-free Medium 199 supplemented with the same concentration of penicillin/streptomycin for the duration of the experiments. Male and female VIC populations were verified using Taqman-based polymerase chain reaction (PCR) for the SRY gene present on the Y-chromosome following methods described in an earlier section.

Proliferation was quantified using the Click-iT EdU Alexa Fluor 488 Imaging Assay (Invitrogen, Carlsbad, CA) and normalized to 4',6-diamidino-2-phenylindole (DAPI) fluorescence. Briefly, cells were seeded in media supplemented with 1% charcoal/dextran treated FBS and then refed on day 1 without serum. VICs were refed 24 hours later and cultured for 24 hours before beginning the Click-iT EdU assay. Following manufacturer’s instructions, EdU was added to the culture media to achieve a final concentration of 10 μM and the Click-iT reaction cocktail was added 18 hours after the addition of EdU. After 25 minutes incubation at room temperature, wells were washed with blocking buffer and the antibody reaction solution was added for a 30-minute incubation. Fluorescent proliferating cells were counted using ImageJ software (NIH) with fluorescent microscope images (Olympus IX51, Olympus, Center Valley, PA) and normalized to the number of total cells indicated by DAPI fluorescence.

Apoptosis was quantified on day 5 of culture using the Caspase-Glo 3/7 Assay System (Promega, Madison, WI) per manufacturer’s instructions and normalized to DNA measured using the PicoGreen dsDNA assay (Invitrogen) in each culture well. Briefly, the Caspase-Glo buffer was added to the kit substrate and equal volume of this reagent was then added to each well of a white 96-well TCPS plate containing VICs. After 2.5 hours incubation at room temperature, luminescence was quantified on a Veritas microplate luminometer (Promega, Madison, WI) and normalized to DNA measured with PicoGreen according to manufacturer’s instructions.

**REFERENCES**

1. Gentleman RC, Carey VJ, Bates DM, Bolstad B, Dettling M, Dudoit S, Ellis B, Gautier L, Ge Y, Gentry J, Hornik K, Hothorn T, Huber W, Iacus S, Irizarry R, Leisch F, Li C, Maechler M, Rossini AJ, Sawitzki G, Smith C, Smyth G, Tierney L, Yang JY, Zhang J. Bioconductor: Open software development for computational biology and bioinformatics. *Genome Biol*. 2004;5:R80

2. Wilson CL, Miller CJ. Simpleaffy: A bioconductor package for affymetrix quality control and data analysis. *Bioinformatics*. 2005;21:3683-3685

3. Shakya K, Ruskin HJ, Kerr G, Crane M, Becker J. Comparison of microarray preprocessing methods. *Adv. Exp. Med. Biol.*;680:139-147

4. Smyth GK. Linear models and empirical bayes methods for assessing differential expression in microarray experiments. *Stat Appl Genet Mol Biol*. 2004;3:Article3

5. Falcon S, Gentleman R. Using gostats to test gene lists for GO term association. *Bioinformatics*. 2007;23:257-258

6. Shengdar Tsai JPC, Bradley A. Feking, Daniel J. Nonneman, Gary A. Rohrer, Jorge A. Piedrahita. Annotation of affymetrix porcine genome microarray chip. *Anim. Genet.* 2006;37:423-424

7. Livak KJ, Schmittgen TD. Analysis of relative gene expression data using real-time quantitative PCR and the 2(-delta delta C(t)) method. *Methods*. 2001;25:402-408

8. Johnson CM, Hanson MN, Helgeson SC. Porcine cardiac valvular subendothelial cells in culture: Cell isolation and growth characteristics. *J. Mol. Cell. Cardiol.* 1987;19:1185-1193
